# Exploring novel nitrofuranyl sulfonohydrazides as anti‐*Leishmania* and anti‐cancer agents: Synthesis, in vitro efficacy and hit identification

**DOI:** 10.1111/cbdd.14097

**Published:** 2022-06-21

**Authors:** Christina Kannigadu, Janine Aucamp, David D. N'Da

**Affiliations:** ^1^ Centre of Excellence for Pharmaceutical Sciences North‐West University Potchefstroom South Africa

**Keywords:** leishmaniasis, leukemia, melanoma, nitrofuran, sulfonohydrazide

## Abstract

Leishmaniasis and cancer are two deadly diseases that plague the human population. There are a limited number of drugs available for the treatment of these diseases; however, their overuse has resulted in pathogenic resistance. Recent studies have indicated the repurposing of nitro‐containing compounds to be a new avenue into finding new treatments. In this study, new nitrofuranyl sulfonohydrazide derivatives were synthesized and evaluated for their in vitro antileishmanial and anticancer activities. The analogue **2h**, featuring biphenyl moiety exhibited selective (SI > 10) submicromolar activity (IC_50_ 0.97 μM) against acute promyelocytic leukemia cells hence was identified anticancer hit. This study revealed no antileishmanial hit. However, several promising analogues were uncovered and are worthy of further structural modifications to improve their toxicity and bioactivity profiles.

## Graphical abstract

Series of nitrofuranyl sulfonohydrazides were synthesized and evaluated in vitro for antileishmanial and anti‐cancer activities. Analogue **2h** (IC_50_ 0.97 μM; SI 17) was identified as anticancer hit against acute promyelocytic leukemia. No antileishmanial hit was uncovered.

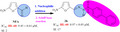

**Abbreviations**
ACS: American cancer society
AMB: Amphotericin B
CL: Cutaneous leishmaniasis
cNFs: clinical nitrofurans
DTIC: Dacarbazine
EM: emetine
FZD: furazolidone
MCL: mucocutaneous leishmaniasis
NFA: 5‐nitro‐2‐furaldehyde
NFT: nitrofurantoin
NFX: Nifuroxazide
NFZ: nitrofurazone
PN: parthenolide
VL: visceral leishmaniasis
WHO: World Health Organization

## 1 INTRODUCTION

Leishmaniasis and cancer are both deadly diseases that pose a major threat to humans, causing significant morbidity and mortality worldwide (Waseem et al., 2017). Their overlapping prevalence in low‐ to middle‐income populations (WHO, 2022a) as well as the carcinogenic risks of *Leishmania* infection (Al‐Kamel, 2017; Schwing et al., 2019) also promotes incidences of comorbidity. Causal associations have consequently been identified between these diseases (Al‐Kamel, 2017; Kopterides et al., 2007). Accordingly, this study focuses on the discovery of compounds with dual activities against leishmaniasis and cancer for the potential treatment of leishmaniasis‐cancer comorbidity.

Leishmaniasis is a high‐priority disease that mainly affects poor people and is accordingly generally associated with malnutrition, population displacement, poor housing, a weak immune system, and a lack of resources (Pervez et al., 2014). It is prevalent in tropical and sub‐tropical developing countries and is endemic to Asia, Africa, the Americas, and the Mediterranean region (WHO, 2022a). Leishmaniasis is caused by the intramacrophage protozoa of the genus *Leishmania*. It is transmitted by the bite of an infected phlebotomine sand fly and can be observed in three different clinical manifestations: cutaneous leishmaniasis (CL), mucocutaneous leishmaniasis (MCL), and visceral leishmaniasis (VL). CL is the most common form, resulting in long‐term skin lesions and debilitating scars, while VL is the most severe form, causing systemic infection and imminent death if left untreated (Pervez et al., 2014; WHO, 2022a). The World Health Organization (WHO) reported 50,000 to 90,000 new cases of VL and 700,000 to 1 million cases of CL worldwide in 2021 (WHO, 2022a).

The treatment of leishmaniasis relies solely on chemotherapy. Sodium stibogluconate and meglumine antimoniate are first line drugs against all forms of leishmaniasis (Ghorbani & Farhoudi, 2018; Kannigadu et al., 2021). In severe cases, the second line drugs, miltefosine, pentamidine, paromomycin, and liposomal amphotericin B, are also used either individually or in combination to treat leishmaniasis (Kannigadu et al., 2021). However, these drugs are all toxic and, apart from miltefosine, all have a poor oral bioavailability hence must be administered intravenously. Miltefosine is the only clinically available oral drug against leishmaniasis; however, it can only treat certain forms of leishmaniasis, which limits its use (Kannigadu et al., 2021). The lack of effective measures to control both the parasite and the sand fly vectors are major factors for the spread of disease. Current therapies are inadequate to manage leishmaniasis due to the development of resistance and the overuse of these limited drugs; hence, it has become increasingly important that new, effective, and cost‐efficient therapies are developed (Deep et al., 2017).

Furthermore, low‐ to middle‐income populations are also significantly susceptible to cancer, a disease in which abnormal cells divide rapidly, spread to other parts of the body, and destroy body tissue. According to estimates from the WHO, cancer is the second leading cause of death globally and it was reported to cause nearly 10 million deaths in 2020, of which 70% occurred in low‐ and middle‐income countries (WHO, 2022b). There are more than a hundred types of cancer; however, in this study, skin cancer and leukemia were investigated. This is due to their overlap with leishmaniasis pathophysiology (skin lesion formation and immune cell infection) (Al‐Kamel, 2017; Morsy, 2013; Schwing et al., 2019). Instances of skin cancer and leukemia comorbidity with leishmaniasis have also been reported (Camillo‐Larco et al., 2019; Moniot et al., 2018).

**FIGURE 1:**
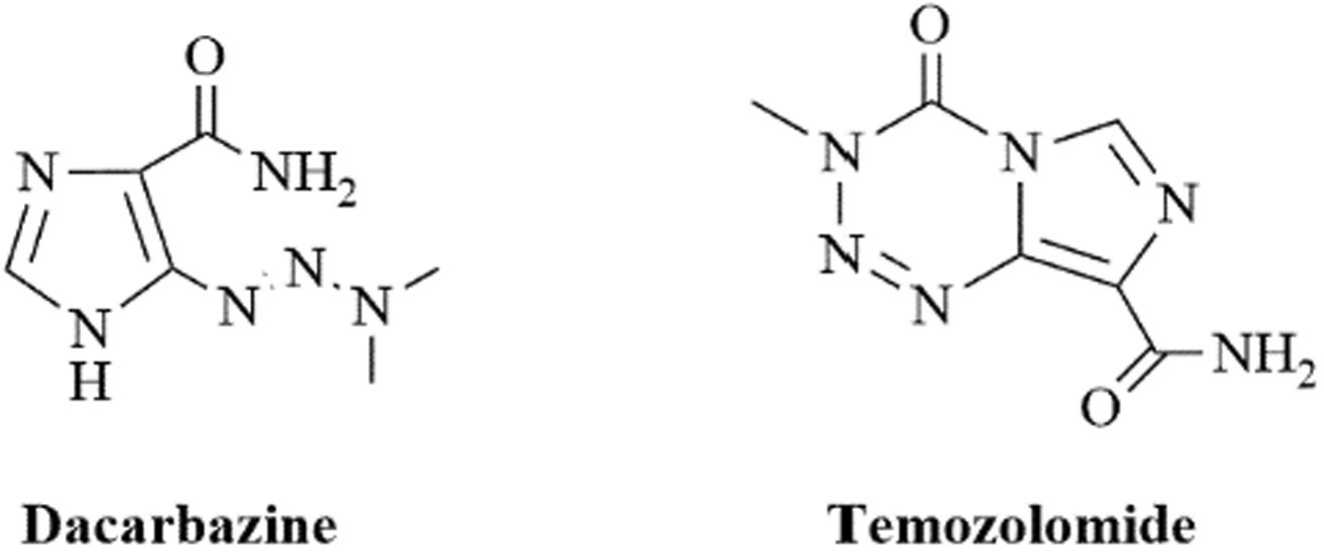
Antimelanoma drugs, dacarbazine and temozolomide

Skin cancer consists of two main types, namely non‐melanoma skin cancers and malignant melanomas (WHO, 2022b). Non‐melanoma skin cancers are the most common, consisting of basal cell and squamous cell carcinomas, whereas malignant melanoma is less prevalent, but leads to most skin cancer‐related deaths. In 2020, 1.20 million new cases of non‐melanoma skin cancers (excluding basal cell carcinoma) were reported globally (WHO, 2022b) with the related deaths of 64,000 according to estimates from Global Cancer (Globocan, 2022). Ultraviolet (UV) radiation plays a role in the development of both cancer types, but genetic and personal characteristics also affect the risks for melanoma (ACS, 2021b; WHO, 2022b). In most cases, skin cancers can be surgically removed; however, surgical treatment may be painful and disfiguring. Severe forms of melanoma can, however, be treated with drugs such as dacarbazine (DTIC) and temozolomide (Figure 1; Ugurel et al., 2013).

**FIGURE 2:**
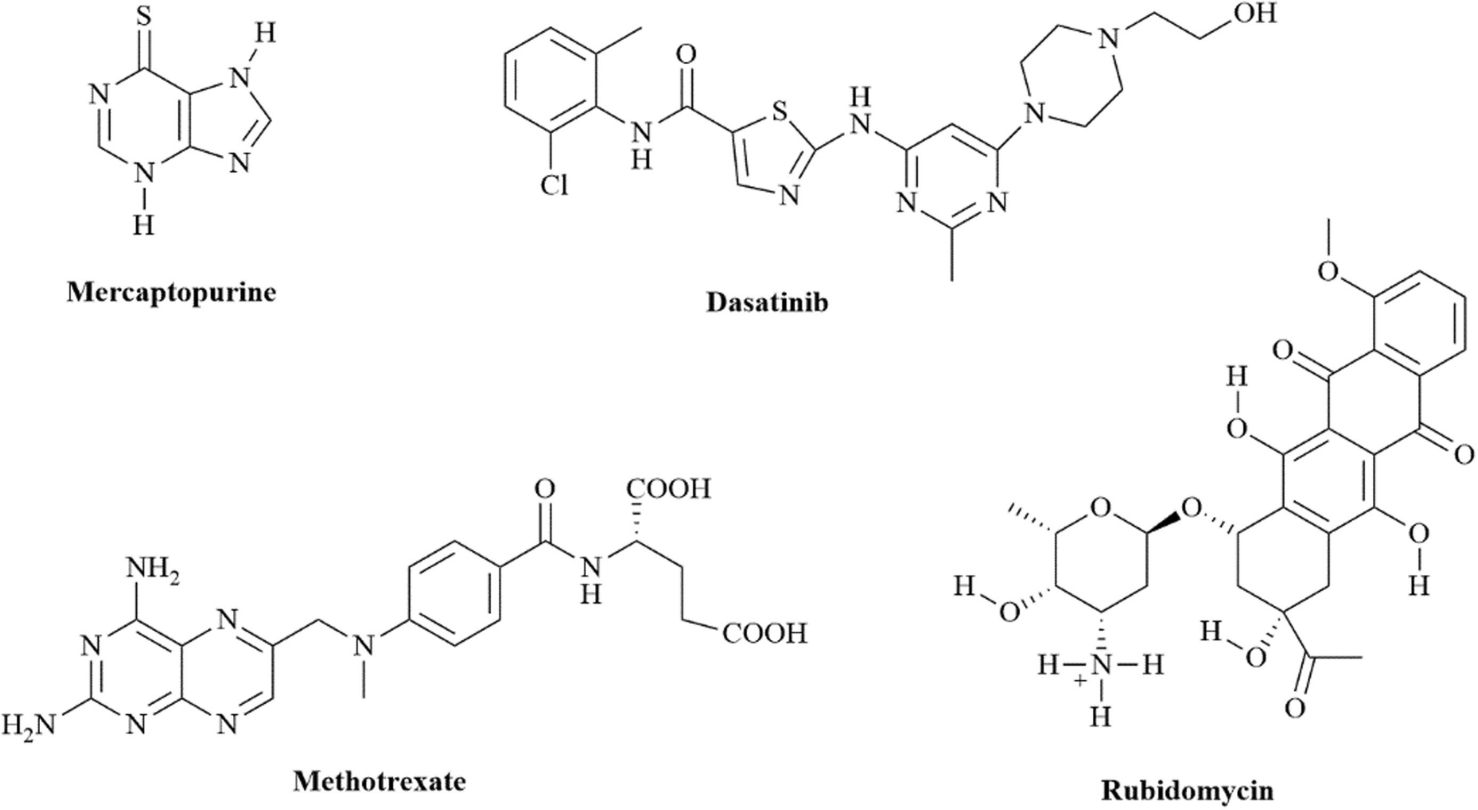
Antileukemic drugs

Leukemia refers to cancer of the blood‐forming cells of the bone marrow and is caused by abnormalities in hematopoietic stem cell and/or progenitor maturation, proliferation, and mortality (ACS, 2018, 2021a). The type of leukemia depends on which hematopoietic cell (myeloid or lymphocytic) and whether premature or mature leukocytes (acute or chronic leukemia, respectively) are involved (ACS, 2018). In 2021, over 61,090 new cases and 23,660 related deaths were reported in the United States (ACS, 2021a). The exact cause of this cancer is still unknown, and the treatment thereof is highly variable. For slow‐growing leukemia, treatment may involve monitoring, whereas for aggressive leukemia, treatment includes chemotherapy with drugs depicted in Figure 2, that is sometimes followed by radiation and stem‐cell transplant (Terwilliger & Abdul‐Hay, 2017).

Both leishmaniasis and cancer may promote the exacerbation of disease progression during comorbidity (Camillo‐Larco et al., 2019; Moniot et al., 2018; Schwing et al., 2019). For example, the development and persistence of acute myeloid leukemia (AML), the most common adult leukemia, is significantly dependent on myeloid cell leukemia 1 (MCL‐1), a critical survival factor for cancer that can directly affect cell death pathways (Kadia et al., 2019). *L. donovani* has been found to exploit MCL‐1 to promote parasite survival by preventing the death of host cells (Giri et al., 2016). This shared biochemical characteristic, i.e. MCL‐1 by both cancer and Leishmania may provide significant therapeutic advantages if targeted in new drugs design. Thus, an antileishmanial drug targeting MCL‐1 may potentially act as an anti‐leukemic and vice versa. Hence, this promotes the potential of drug repurposing, an emerging field in drug discovery used for fast‐tracking the development of new therapies via the use of existing drugs (Jain & Sharma, 2017). This involves the use of de‐risked compounds, with potentially lower overall development costs, shorter development timelines, and minimum risk of failure (Jain & Sharma, 2017). Several numbers of successes have been achieved by this strategy such as the use of thalidomide for leprosy and multiple myeloma (Pushpakom et al., 2019). Furthermore, shared biochemical characteristics also foster the development of multifunctional treatments that can successfully and safely manage comorbidities. Indeed, leishmaniasis and cancer share several potential therapeutic targets that supports the development of multifunctional drugs (Rashidi et al., 2021).

Nitro‐containing drugs, such as the clinical nitrofurans (cNFs), have been well‐documented in literature for their ability in treating a broad range of infectious diseases (Kannigadu et al., 2021; Zuma et al., 2019). These drugs possess several mechanisms of antimicrobial action, which are thought to contribute toward the absence of any pathogenic resistance against them. cNFs possess an array of biological activities that have been attributed to its redox‐active nitro group (NO_2_), which induces oxidative stress under aerobic and anaerobic conditions resulting in microorganism death (Kalia & Raines, 2008). Additionally, these drugs also host a second pharmacophore, the hydrazone moiety, that also possesses intrinsic biological activity, thus making them good candidates for drug repurposing (Ryan, 2017). This is further corroborated by a recent literature (Bailly, 2019) which reports that nitrofuran derivatives that are direct analogues of nifuroxazide (NFX) revealed anticancer properties against cancers such as leukemia and melanoma.

Moreover, sulfonohydrazide derivatives have been reported to elicit antileishmanial (Zafar et al., 2021) and anticancer (Korcz et al., 2018; Zhang et al., 2014) potencies. Their antileishmanial mechanism is not known; however, their anticancer activity is believed to originate from the inhibition of P13 cancer cell kinase p110α resulting in both decrease cellular proliferation and increased cellular death (Hayakawa et al., 2007; Zhang et al., 2014).

Based on this evidence, a series of new nitrofuranyl sulfonohydrazide derivatives were synthesized and their antileishmanial and anticancer activities were examined in vitro to identify potential hits. We herein report the synthesis and biological activities of these compounds.

## 2 EXPERIMENTAL

### 2.1 Chemistry

#### 2.1.1 General

All chemicals and reagents were purchased from various suppliers, and all routinely used procedures, such as spectroscopic techniques, are reported in the Supinfo S1.

#### 2.1.2 General procedure for the synthesis of sulfonyl hydrazide derivatives (**1a–l**)

A literature method was adopted from Karaman et al. (2016) and modified to synthesize these analaogues. The detailed synthesis, data, and spectra are reported in Supinfo S1 for each intermediate.

#### 2.1.3 General procedure for the synthesis of nitrofuranyl sulfonoydrazide derivatives (**2a–l**)

These compounds were synthesized according to an established method adopted from the literature (Elizondo‐Jimenez et al., 2017). Substituted sulfonyl hydrazide (**1a‐1l**; 26.9 mmol, 0.5 g, 1 equiv) was dissolved in ethanol (4 ml). To this mixture, 5‐nitro‐2‐furaldehyde (26.4 mmol, 0.38 g, 1 equiv) and three drops of catalytic hydrochloric acid were added. The reaction was left to stir at room temperature for 12 h and monitored by TLC. Upon completion of the reaction, water was added to the reaction and the precipitate was filtered off. The precipitate was recrystallized in ethyl acetate to afford the analogues as solids. The structures of the synthesized compounds were verified by nuclear magnetic resonance (NMR) spectroscopy. Compounds **2b** (Chao et al., 2016), **2c** (Alsaeedi et al., 2015), and **2g** (Nguyen et al., 1978) have previously been reported but were resynthesized according to this method. The characterization data of synthesized compounds are reported in the Supinfo S1.

### 2.2 In vitro biological assays

#### 2.2.1 Antipromastigote assay

The antipromastigote activity of synthesized compounds was evaluated as described previously by Mangwegape et al. (2021) using three strains of *L. donovani* (1S (MHOM/SD/62/1S) and 9515 (MHOM/IN/95/9515)) and *L. major* (IR‐173 [MHOM/IR/−173]). The assay method is available in the Supinfo S1. All compounds were first screened for growth inhibition at 10 μM (Siqueira‐Neto et al., 2010), and qualifying compounds with growth inhibition >70% were selected for further IC_50_ determination.

#### 2.2.2 Anti‐amastigote assay

The activities of synthesized compounds against the intramacrophage parasites of the three *Leishmania* strains were evaluated using a modified, resazurin‐based method (Jain et al., 2012; Njanpa et al., 2021). Suspension cultures of human acute monocytic leukemia (THP‐1) cells were maintained in RPMI‐1640 medium (Sigma Aldrich) supplemented with 10% FBS and 1% penicillin–streptomycin, at 37°C and 5% CO_2_ in a humidified atmosphere. Duplicate 96‐well plates (for respective anti‐amastigote and cytotoxicity assays) were seeded with 200 μl of a 2.5 × 10^5^ cells/ml suspension treated with 25 ng/ml phorbol 12‐myristate 13‐acetate (PMA), followed by 48‐h incubation to promote differentiation into adherent macrophages.

For the anti‐amastigote assay, differentiated plates were carefully washed with PBS, followed by the addition of 200 μl of stationary phase promastigotes in RPMI 1640 medium with 2% FBS. An MOI of 30:1 was used for all three *Leishmania* strains. The parasite‐treated plates were incubated for 24 h at 32°C (*L. major*) or 37°C (*L. donovani*) and 5% CO_2_ to promote infection of the macrophages. The wells were then washed four times with PBS to remove extracellular parasites, followed by treatment with 200 μl of (i) amphotericin B (Sigma Aldrich; positive control); (ii) growth medium and solvent (negative control to compensate for possible solvent effects); (iii) 10 μM of compound for activity screening; (iv) growth medium with seven twofold dilution concentrations of 10 μM compounds for IC_50_ determination. Blanks were represented by growth medium without cells, as well as parasite‐free THP‐1 cells. The treated plates were incubated for 72 h.

After incubation, the plates were gently washed three times with PBS to remove any remaining extracellular parasites. The wells were then treated with 20 μl of 0.05% sodium dodecyl sulfate in PBS for 30 s to lyse the host macrophages. Lysis was terminated by adding 180 μl promastigote growth medium with 10% FBS. To initiate the resazurin assay, 10 μl of resazurin solution (0.025% in PBS) was added to all wells and the plates were incubated for 24 h at 32°C (*L. major*) or 37°C (*L. donovani*) and 5% CO_2_. Absorbance measurements, calculations, and IC_50_ determinations were performed as described for the antipromastigote assay (Supinfo S1). All compounds were first screened for growth inhibition >60% at 10 μM (De Muylder et al., 2011) and those demonstrating inhibition >60% qualified for IC_50_ determinations.

#### 2.2.3 Cytotoxicity assay

African green monkey kidney epithelial (Vero) cells (Cellonex, South Africa) were cultured and the basal cytotoxicity of the synthesized compounds with antileishmanial and/or anticancer activity was evaluated using the resazurin assay, as previously described (Mangwegape et al., 2021). The assay method is available in the Supinfo S1.

#### 2.2.4 Anticancer assay

The anticancer activities of synthesized compounds were evaluated using a resazurin‐based assay (Czekanska, 2011). Accordingly, a screening method similar to that of the cytotoxicity assay was used to screen for anticancer activity, using human melanoma (A375), promyelocytic leukemia (Clone 15 HL‐60), lung cancer (A549), and breast cancer (MCF7) cells. The culture and assay methods are provided in the Supinfo S1. Potential anticancer hits are considered to have IC_50_ < 10 μM and SI > 10 (Cancer_Research, 2014). Accordingly, all compounds were first screened for growth inhibition >50% at 10 μM and compounds that qualified were further used for IC_50_ determination.

#### 2.2.5 Statistical analysis

In vitro antileishmanial activities and cytotoxicity, indicated as IC_50_ values, were derived from non‐linear regression analysis. Results were represented as the mean ± the standard deviation (SD) from the triplicate biological experiments. Statistical analysis was performed, using SkanIt 4.0 Research Edition software (Thermofisher Scientific) and Prism V5 software (GraphPad). All reported data were significant at *p* < .05.

## 3 RESULTS AND DISCUSSION

### 3.1 Chemistry

**SCHEME 1:**
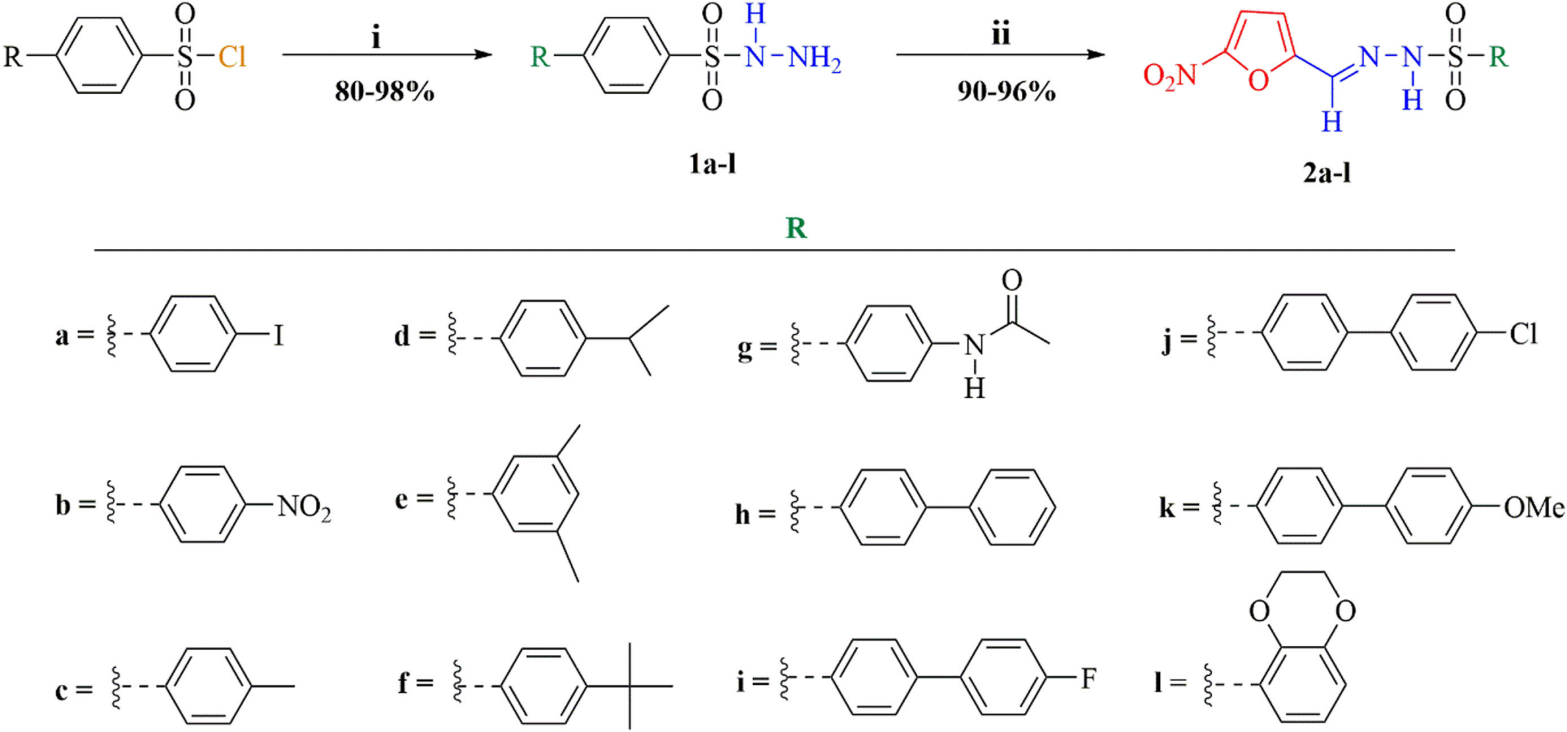
Two‐step synthesis of nitrofuranyl sulfonohydrazide derivatives *reagents and conditions*: (i) NH_2_NH_2_ (10 equiv), TEA (1.5 equiv), DCM, rt, 12 h; (ii) NFA (1 equiv.), HCl (cat.), EtOH, rt, 12 h. [Colour figure can be viewed at wileyonlinelibrary.com]

The nitrofuranyl sulfonohydrazide derivatives (**2a‐l**) were synthesized following a two‐step process starting with commercial sulfonyl chloride (Scheme 1). In the first step, the sulfonyl chlorides were reacted with hydrazine hydrate in dichloromethane in basic medium provided by triethylamine (TEA) to form the desired sulfonyl hydrazide intermediates (**1a–l**; Karaman et al., 2016) in good yields (80%–98%). This was then followed by acid‐catalyzed (HCl) Schiff base reaction of the sulfonyl hydrazides (**1a–l**) with an equimolar 5‐nitro‐2‐furaldehyde (NFA) in ethanolic medium (Elizondo‐Jimenez et al., 2017), and, upon elimination of water, the targeted nitrofuranyl sulfonohydrazide derivatives (**2a–l**) were formed and isolated in excellent yields (90%–96%) after recrystallization from ethyl acetate.

In this series, it should be noted that all intermediates **1a–1** (Terent'ev et al., 2016), and analogues **2b** (Chao et al., 2016), **2c** (Alsaeedi et al., 2015), and **2g** (Nguyen et al., 1978) have previously been reported; hence, they are not novel per se to this work. However, none of these compounds has been investigated for its antileishmanial effect and hence their inclusion in our series. Analogue **2b** was synthesized in a series of Ag(I)‐induced three component annulation reaction of fullerene with sulfonylhydrazones (Chao et al., 2016). Compound **2c** was prepared and investigated for its antimicrobial activity against two Gram‐positive bacteria (*Staphylococcus aureus* (ATCC 25923); *Bacillus subtilis* (ATCC 6633); three Gram‐negative bacteria (*Escherichia coli* (ATCC 25922); *Salmonella susi* (ATTC 13070); *Pseudomonas aeruginosa* (ATCC 27853)) and one fungus *Candida albicans* (ATCC 10231) by determining its inhibition zone values using a diffusion method. The inhibition zone of *Salmonella susi* of compound **2c** was determined to be 20 mm, and no activity was observed for any other of the strains tested. No further tests were done to determine its IC_50_’s (Alsaeedi et al., 2015). Lastly, a very old literature reported the synthesis of compound **2g** as part of a 5‐nitrofurfural sulfonylhydrazone series (Nguyen et al., 1978).

The structures of all compounds were confirmed by routine molecular characterization techniques, NMR (^1^H and ^13^C), high‐resolution spectrometry (HRMS), and Fourier transformed infrared spectroscopy. For example in derivative **1a** (the 4‐iodo derivative), a characteristic singlet at ca. *δ* 8.45 ppm was observed, and this peak was assigned to the resonance of the iminyl acidic proton H‐2, and the singlet at ca. *δ* 4.17 ppm was supportive of the presence of H‐1 proton of the amino NH_2_ group. Similarly, this was observed in all the spectra of the intermediates **1a–l**. The disappearance of H‐1 peak from the spectra of **2a** and the shift of H‐2 peak to ca. *δ* 12.27 ppm further validated the success of the Schiff base reaction. The ^1^H spectra of all analogues possess a singlet ca. *δ* 7.88 ppm which was assigned to the vinylic proton H‐3′ of the hydrazone bond, whereas the furanyl protons H‐6′ and H‐7′ were represented by two doublets (*d*) with *J* = 3.9 Hz at *δ* 7.72 and *δ* 7.15 ppm, respectively. The deshielding of H‐6′ was due to the nitro group that withdraws electron density away from it, while the unsaturated hydrazone bond enhanced the electron density of H‐7′. The phenyl ring attached to the sulfonyl showed two aromatic resonances that appeared as a pair of coupled doublets at *δ* 8.03 and *δ* 7.61 ppm (^3^ *J* = 8.4 Hz), which were attributed to the aromatic protons H‐2 and H‐3, respectively. The **2i** derivative presented with an aromatic resonance represented by a doublet of doublets (dd) in the *δ* 7.79–7.33 ppm region with *J*_H‐H_ = 8.8 and ^3^ *J*_H‐F_ = 5.4 Hz assigned to resonance of proton H‐7, with the later doublet resulting from the coupling of H‐7 with adjacent F atom. In summary, all the protons that were expected for each analogue were accounted for in the NMR spectra.

The ^13^C spectra of all analogues showed the singlet at *δ* 135.27 ppm assignable to the vinylic carbon (C‐3′) of the hydrazone moiety, whereas the four aromatic carbon of the furan ring appeared as singlets at δ 151.85 (C‐5′), δ 150.53 (C‐4′), *δ* 115.57 (C‐7′), and δ 114.37 (C‐6′). The aromatic carbons of the phenyl ring appeared as singlets at *δ* 138.39 (C‐3) and *δ* 128.71 (C‐2). In **2i**, the carbon C‐8 resonated as a doublet at *δ* 162.53 ppm resulting from the coupling with adjacent fluorine with ^1^ *J*_C‐F_ = 245.4 Hz. The carbon C‐7 expressed a weaker coupling with the fluorine (2 bonds away) showing a doublet at *δ* 115.99 ppm with ^2^ *J*_C‐F_ = 21.7 Hz and C‐6 demonstrated an even weaker coupling the fluorine (three bonds further) evidenced by the doublet at *δ* 139.30 ppm with ^3^ *J*_C ‐F_ = 7.7 Hz.

IR analysis further confirmed the success of the Schiff base reaction (**2a–l**) by the appearance of characteristic absorption of C=N stretching (1600–1560 cm^−1^), N‐O stretching (1550–1500 and 1350–1300 cm^−1^), and the S=O stretching (1410–1380 and 1370–1335 cm^−1^).

Furthermore, HRMS using atmospheric pressure chemical ionization source confirmed the integrity of the synthesized analogues as in all cases, the molecular ions determined experimentally were in agreement with the calculated values based on the suggested chemical formulae.

Moreover, in these analogues, all substituents on the imine group (RR′C=N‐R″) were found to be in the *E* configuration. The Z_C=N_ configuration is not observed because of steric hinderance and the non‐planar conformation of C=N‐NH does not exist because it would violate the n‐π‐conjugation. This was determined by running a selective 1D NOESY and confirmed by the literature report in Syakaev et al. (2006).

Furthermore, the chemical shifts for NH signals of Z_C=N_ isomers of N‐acylhydrazones by extension N‐sulfonylhydrazones, the latter being more electron withdrawing inductively, are reported around 14 ppm (Reis et al., 2013); no signal in this region was observed for the synthesized compounds.

### 3.2 Predicted physicochemical and pharmacokinetic properties

Oral administration is the preferred route of drug delivery due to several advantages it offers, such as safety, convenience, good patient compliance, ease of ingestion, pain avoidance, and versatility to accommodate various types of drugs (Sastry et al., 2000). The physicochemical properties such as lipophilicity and aqueous solubility influence oral delivery of a drug hence were herein predicted for all the synthesized compounds using the SwissADME web tool. The data are shown in Table 1. Lipinski's rule of five (Lipinski et al., 2001) was then used to predict the druglikeness and oral activity of the derivatives in humans.

**TABLE 1:** Physicochemical and ADME data of synthesized nitrofuran sulfonyl hydrazine derivatives and standard nitrofuran drugs as predicted by SwissADME web tool, http://www.swissadme.ch [date of access: 11/01/2022]

Cpd.	MW (g/Mol)	Log *P*_o/w_ ^a^	RB^b^	Log *S* ^c^		TPSA (Å^2^)^f^	HBD^g^	HBA^h^	Lipinski's violation	GI absorption	Leadlike‐ness^i^	Druglikeness^j^
				ESOL^d^	Ali^e^							
**NFA**	141.08	0.36	2	−1.59	−2.20	76.03	4	2	0	High	No	Yes
**1a**	298.10	0.93	2	−2.25	−1.77	80.57	2	4	0	High	Yes	Yes
**1b**	217.20	−0.70	3	−1.11	−1.88	126.39	2	6	0	High	No	Yes
**1c**	186.23	0.56	2	−1.37	−1.47	80.57	2	4	0	High	No	Yes
**1d**	214.28	1.19	3	−1.91	−2.27	80.57	2	4	0	High	No	Yes
**1e**	200.26	0.96	2	−1.84	−2.15	80.57	2	4	0	High	No	Yes
**1f**	228.31	1.44	3	−2.32	−2.83	80.57	2	4	0	High	No	Yes
**1 g**	229.26	−0.20	4	−1.02	−1.40	109.67	3	5	0	High	No	Yes
**1h**	248.30	1.66	3	−2.64	−2.79	80.57	2	4	0	High	No	Yes
**1i**	266.29	1.97	3	−2.79	−2.89	80.57	2	5	0	High	Yes	Yes
**1j**	282.75	2.19	3	−3.22	−3.43	80.57	2	4	0	High	Yes	Yes
**1k**	278.33	1.71	4	−2.87	−3.24	89.80	2	5	0	High	Yes	Yes
**1l**	230.24	0.10	2	−1.34	−1.49	99.03	2	6	0	High	No	Yes
**2a**	421.17	1.67	5	−4.23	−5.03	125.87	1	6	0	High	No	Yes
**2b**	340.27	0.33	6	−3.11	−5.14	171.69	1	8	1	Low	Yes	Yes
**2c**	309.30	1.36	5	−3.36	−4.74	125.87	1	6	0	High	Yes	Yes
**2d**	337.35	2.00	6	−3.91	−5.53	125.87	1	6	0	High	Yes	Yes
**2e**	323.32	1.69	5	−3.65	−5.11	125.87	1	6	0	High	Yes	Yes
**2f**	351.38	2.16	6	−4.32	−6.09	125.87	1	6	0	High	No	Yes
**2 g**	352.32	0.65	7	−2.70	−4.11	154.97	2	7	0	Low	No	Yes
**2h**	371.37	2.41	6	−4.57	−6.04	125.87	1	6	0	Low	No	Yes
**2i**	389.36	2.65	6	−4.72	−6.15	125.87	1	7	0	Low	No	Yes
**2j**	405.81	2.93	6	−5.15	−6.69	125.87	1	6	0	Low	No	Yes
**2k**	401.39	2.44	7	−4.63	−6.21	135.10	1	7	0	Low	No	Yes
**2l**	353.31	0.83	5	−3.17	−4.45	144.33	1	8	0	Low	No	Yes
**NFX**	275.22	0.90	5	−2.95	−4.27	120.65	2	6	0	High	Yes	Yes
**FZD**	225.16	0.32	3	−1.24	−1.62	100.86	0	6	0	High	No	Yes
**NFZ**	198.14	−0.59	4	−1.21	−2.45	126.44	2	5	0	High	No	Yes
**NFT**	238.16	−0.50	3	−1.04	−1.60	120.73	1	6	0	High	No	Yes

^a^ Calculated log*P* (consensus log *P*).

^b^ Number of rotatable bonds.

^c^ Predicted aqueous solubility, where log *S* is the logarithm of the amount of compound (in moles) able to dissolve a liter of water.

^d^ ESOL = estimated aqueous solubility, calculated using a topological method (Delaney, 2004).

^e^ Calculated using a topological method (Ali et al., 2012) with log *S* scale: insoluble < −10 < poorly < −6 < moderately < −4 < soluble < −2 very soluble < 0 highly <.

^f^ Topological polar surface area, RB ≤ 10 and TPSA ≤ 140 Å^2^ – good oral bioavailability (Veber et al., 2002).

^g^ Number of hydrogen bond donors (NH and OH groups).

^h^ Number of hydrogen bond acceptors (nitrogen and oxygen atoms).

^i^ According to Teague et al., 250 ≤ MW ≤ 350, XLOGP ≤ 3.5 and RB ≤ 7 (Teague et al., 1999).

^j^ Determined with reference to Lipinski's rule of five: MW ≤500 g/mol; Log*P* ≤5; HBD ≤ 5; HBA ≤ 10; no more than one violation allowed (Lipinski et al., 2001).All values in this table were calculated using SwissADME web tool, http://www.swissadme.ch. (Daina et al., 2017). NFX: nifuroxaxide; NFA: 5‐nitro‐2‐furaldehyde; FZD: furazolidone; NFZ: nitrofurazone; NFT: nitrofurantoin.

All of the analogues complied with Lipinski's rules and had physicochemical properties well within the target ranges (Lipinski et al., 2001). Most of the analogues were also predicted to be highly absorbed in the GI tract through passive diffusion, were expected to be druglike in nature and suitable for oral administration (except **2b** and **2g**; Veber et al., 2002), and had potential leads in compliance with criteria defined by Teague et al. (1999).

### 3.3 Pharmacology

The development of new drugs faces several challenges (e.g., scientific) which lengthens the process. To fast‐track it, experts in drug discovery have devised several strategies. For instance, at basic research level, criteria have been defined to quickly identify hit and lead compounds. Regarding infectious diseases, such as those occurring in developing countries, with focus on leishmaniasis, Katsuno et al. (2015) established that a validated hit compound should possess inter alia. Cellular potency IC_50_ < 10 μM and selectivity index, SI > 10 for parasite in the presence of mammalian (e.g., Vero, HepG2) cells while a validated lead should be credited with IC_50_ < 1 μM and selectivity index, SI > 100.

Moreover, Cancer Research UK (Cancer_Research, 2014) established that an anticancer hit among other criteria should demonstrated IC_50_ < 10 μM and selectivity index, SI > 10 for the undesired targets, that is, cancer cells, in biochemical assay. Based on these criteria, literature reported biochemical assays were used to determine the activity of the compounds.

#### 3.3.1 Antileishmanial activity

The synthesized analogues were evaluated for their in vitro antileishmanial activity against three strains of *Leishmania*. Amphotericin B (AMB) was used as the standard antileishmanial drug, whereas the clinical antibiotics, NFX, furazolidone, nitrofurazone, and nitrofurantoin were used as reference drugs alongside 5‐nitro‐2‐furaldehyde, the precursor of the analogues. The *Leishmania* used were *L. donovani* strains (1S and 9515) and *L. major* strain IR‐173, which were selected to determine the specificity of the synthesized compounds against *L. major* parasites that cause CL, and *L. donovani* parasites that communicate the more serious and devastating VL to humans (Siqueira‐Neto et al., 2010).

Furthermore, *Leishmania* presents with two developmental forms, promastigote in the vector and amastigote in the mammal host (e.g., human). The latter is responsible for the infection's symptoms and progression to the disease hence is consensually agreed upon as the most clinically relevant form in the process of antileishmanial new therapies development. Nevertheless, the synthesized compounds were tested against both forms *Leishmania* parasite.

The intermediates, analogues, and reference drugs were first screened at a single point 10 μM concentration. Those resulting in 70% and 60% growth inhibition against the promastigotes (Siqueira‐Neto et al., 2010) and amastigotes (De Muylder et al., 2011), respectively, were selected for further antileishmanial activity assessment through IC_50_ determination. The intermediates **1a‐l** exhibited no growth inhibitory activity thus were not reported. On contrary, analogues **2a‐l** provided several cases of antipromastigote activity with 70% growth inhibition and anti‐amastigote growth inhibition values within the 60% cutoff.

All qualifying analogues were subjected to IC_50_ determination. These analogues were also evaluated for their systemic/basal cytotoxicity using Vero cells and specific toxicity using THP‐1 macrophages. All data are shown in Tables 2 and 3.

**TABLE 2:** Antileishmanial results of synthesized nitrofuranyl sulfonohydrazide derivatives and other nitrofuran antibiotics against the *L. major* strain IR‐173

Compd.	Cytotoxicity, IC_50_ (μM) ± SD		Antileishmanial activity, IC_50_ (μM) ± SD		Specificity index^a^ (SpI_1_^b^)	Selectivity index	
	Vero	THP‐1	Promastigote	Amastigote		SI_1_^c^	SI_2_^d^
**NFA**	23.15 ± 2.62	80.92 ± 6.37	7.28 ± 0.10	8.35 ± 0.89	0.87	3	10
**2a**	–	–	>10	>10	–	–	–
**2b**	–	–	>10	>10	–	–	–
**2c**	–	–	>10	>10	–	–	–
**2d**	22.18 ± 0.80	93.07 ± 3.51	4.71 ± 0.33	8.30 ± 0.78	0.57	3	11
**2e**	39.80 ± 5.89	> 100	7.09 ± 0.74	>10	–	–	–
**2f**	29.18 ± 5.86	84.59 ± 2.16	6.93 ± 0.83	>10	–	–	–
**2g**	‐	‐	>10	>10	–	–	–
**2h**	16.35 ± 5.80	50.36 ± 2.26	2.49 ± 0.51	>10	–	–	–
**2i**	29.15 ± 5.41	–	>10	>10	–	–	–
**2j**	–	–	>10	>10	–	–	–
**2k**	–	–	>10	>10	–	–	–
**2l**	41.23 ± 3.30	>100	8.10 ± 0.03	>10	–	–	–
**NFX**	>100	>100	>10	>10	–	–	–
**FZD**	>100	>100	0.34 ± 0.03	2.80 ± 0.50	–	36	36
**NFZ**	>100	>100	1.85 ± 0.06	5.75 ± 0.75	0.32	17	17
**NFT**	>100	>100	>10	>10	–	–	–
**AMB**	57.77 ± 3.22	14.86 ± 0.09	0.03 ± 0.006	0.03 ± 0.00	1.00	1925	495
**EM**	0.08 ± 0.009	–	–	–	–	–	–

**TABLE 3:** Antileishmanial results of synthesized nitrofuranyl sulfonohydrazide derivatives and other nitrofuran antibiotics against the *L. donovani* strains 1S and 9515

Compd	1S IC_50_ (μM) ± SD		9515 IC_50_ (μM) ± SD		Specificity index^a^		Selectivity index			
	Promastigote	Amastigote	Promastigote	Amastigote	SpI_2_ ^e^	SpI_3_ ^f^	SI_3_ ^g^	SI_4_ ^h^	SI_5_ ^i^	SI_6_ ^j^
**NFA**	6.39 ± 0.26	>10	>10	8.56 ± 0.65	–	–	–	–	3	9
**2a**	>10	>10	>10	>10	–	–	–	–	–	–
**2b**	>10	>10	>10	>10	–	–	–	–	–	–
**2c**	>10	>10	>10	>10	–	–	–	–	–	–
**2d**	5.00 ± 1.32	>10	3.62 ± 0.65	>10	–	–	–	–	–	–
**2e**	3.04 ± 0.04	>10	3.97 ± 1.08	6.14 ± 0.83	–	0.65	–	–	6	16
**2f**	5.29 ± 0.69	6.36 ± 0.00	3.50 ± 0.58	9.62 ± 1.15	0.83	0.36	5	13	3	9
**2g**	>10	>10	–	–	–	–	–	–	–	–
**2h**	1.45 ± 0.16	2.94 ± 0.30	1.53 ± 0.14	6.29 ± 0.62	0.49	0.24	6	17	3	8
**2i**	9.38 ± 0.82	>10	>10	>10	–	–	–	–	–	–
**2j**	>10	>10	>10	>10	–	–	–	–	–	–
**2k**	>10	>10	>10	>10	–	–	–	–	–	–
**2l**	9.24 ± 1.32	6.36 ± 0.00	7.25 ± 0.11	7.85 ± 0.21	1.45	0.92	6	16	5	13
**NFX**	>10	>10	>10	7.29 ± 0.39	–	–	–	–	14	14
**FZD**	0.32 ± 0.00	1.94 ± 0.00	0.28 ± 0.04	4.11 ± 0.78	0.16	0.07	52	52	24	24
**NFZ**	6.54 ± 0.93	2.50 ± 0.40	1.85 ± 0.14	4.94 ± 0.61	2.62	0.37	40	40	20	20
**NFT**	>10	>10	>10	8.56 ± 1.95	–	–	–	–	–	–
**AMB**	0.02 ± 0.00	0.04 ± 0.00	0.02 ± 0.003	0.05 ± 0.00	0.50	0.40	1444	372	1155	297

^a^ Specificity index (SpI) < 0.4 indicates more antipromastigote activity, 0.4 < SpI < 2.0 indicates activity against both forms, SpI > 2.0 indicates more anti‐amastigote activity (De Muylder et al., 2011).

^b^Specificity index of *L. major* IR‐173: SpI_1_ = IC_50_ promastigote/IC_50_ amastigote.

^c^Selectivity Index of *L. major*: SI_1_ = IC_50_ Vero/IC_50_ amastigote.

^d^ Selectivity Index of *L. major*: SI_2_ = IC_50_ THP‐1/IC_50_ amastigote.

^e^ Specificity index of *L. donovani* 1S: SpI_2_ = IC_50_ promastigote/IC_50_ amastigote.

^f^ Specificity index of *L. donovani* 9515: SpI_3_ = IC_50_ promastigote/IC_50_ amastigote.

^g^ Selectivity Index of *L. donovani* 1S: SI_3_ = IC_50_ Vero/IC_50_ amastigote.

^h^ Selectivity Index of *L. donovani* 1S: SI_4_ = IC_50_ THP‐1/IC_50_ amastigote.

^i^ Selectivity Index of *L. donovani* 9515: SI_5_ = IC_50_ Vero/IC_50_ amastigote.

^j^ Selectivity Index of *L. donovani* 9515: SI_6_ = IC_50_ THP‐1/IC_50_ amastigote; Vero: African green monkey kidney epithelial cells; THP‐1: Human acute monocytic leukemia; AMB: amphotericin B; EM: Emetine; Blue = SI above 10; Red = compounds qualifying as antileishmanial hits (Katsuno et al., 2015). All data reported in Tables 2 and 3 were significant at *p* < .05.

The basal cytotoxicity data indicated that the analogues exhibited moderate toxicity to mammalian Vero cells (IC_50_ < 40 μM). Consequently, the low selectivity indices of most analogues were indicative of their non‐intrinsic antileishmanial activity.

Only one analogue, **2d**, presented with anti‐amastigote activity with IC_50_ < 10 μM against *L. major*, suggesting that this nitrofuranyl sulfonohydrazide had its activity directed toward both parasitic forms (SpI 0.57; De Muylder et al., 2011) with a slight preference for the promastigote form and thus could serve as potential antileishmanial hit (IC_50_ < 10 μM, SI_2_ > 10; Katsuno et al., 2015) relative to the host THP‐1 cells. However, its moderate basal cytotoxicity on Vero cells (SI_1_ < 10) disqualified it. Nonetheless, this analogue may stand as a suitable candidate for further structural modification to improve its cytotoxic liability.

Moreover, analogues **2e**, **2f**, **2h**, and **2l** demonstrated micromolar anti‐amastigote activity against the *L. donovani* strains (1S and 9515) with high selectivity indices (SI > 10) relative to the host THP‐1 cells. However, their moderate basal toxicities toward Vero cells (SI < 10) disqualified them as potential hits. These analogues possessed between twofold and 23‐fold superior antipromastigote potencies with similar anti‐amastigote potencies, in comparison with the parent NFA and the clinical antibiotics (except FZD). The overall best performer was **2h** with IC_50_ 1.45 ± 0.16 μM and 1.53 ± 0.14 μM against *L. donovani* (1S and 9515) promastigotes, respectively, as well as 2.94 ± 0.30 μM and 6.29 ± 0.62 μM against *L. donovani* (1S and 9515) amastigotes, respectively. However, these potencies were not bona fide as evidenced by the toxicities on Vero (SI_3_ and SI_4_ < 10) and THP‐1 (SI_6_ < 10) cells in the presence of *L. donovani* parasites.

Overall, no antileishmanial hit was uncovered among the synthesized nitrofuranyl sulfonohydrazide derivatives while the cNF antibiotics, NFZ and FZD were confirmed as antileishmanial hits against all three strains. The standard antileishmanial drug AMB proved to be an uncontestable lead also against all the strains while NFX was revealed as hit only against the antimonial‐resistant strain *L. donovani* 9515 (Potvin et al., 2021). NFT was not identified as hit against any of the strains.

#### 3.3.2 Anticancer activity

Due to reports of skin cancer and leukemia comorbidity with leishmaniasis (Camillo‐Larco et al., 2019; Moniot et al., 2018), anticancer screenings were also included in this study. A375 cells were used to screen for activity against malignant melanoma, the most dangerous skin cancer form (WHO, 2022b), whereas Clone 15 HL‐60 cells were used to screen for activity against promyelocytic leukemia, a subtype of the most common form of adult leukemia, AML (Kadia et al., 2019). Potential anticancer hits are expected to have an IC_50_ < 10 μM and SI > 10 (Cancer_Research, 2014). Preliminary screenings, using a cutoff value of 50% growth inhibition at 10 μM for further anticancer IC_50_ determination, indicated that only compound **2h** qualified for further testing against A375 cells while compounds **2b**, **2c**, **2h**, and **2e** qualified for further testing against Clone 15 HL‐60. These results are shown in Table 4.

**TABLE 4:** Anticancer results of synthesized nitrofuranyl sulfonohydrazide derivatives

Compd	Anticancer activity, IC_50_ ± SD (μM) (*n* = 3)			
	Clone 15 HL‐60	SI_1_ ^a^	A375	SI_2_ ^b^
**NFA**	9.45 ± 0.61	2	>10	–
**2a**	>10	–	>10	–
**2b**	9.32 ± 0.90	3	>10	–
**2c**	>10	–	>10	–
**2d**	7.85 ± 0.38	3	>10	–
**2e**	>10	–	>10	–
**2f**	4.99 ± 0.08	6	>10	–
**2g**	–	–	>10	–
**2h**	**0.97 ± 0.03**	17	8.05 ± 0.57	2
**2i**	>10	–	>10	–
**2j**	>10	–	>10	–
**2k**	>10	–	>10	–
**2l**	>10	–	>10	–
**NFX**	8.97 ± 0.47	11	>10	–
**NFT**	>10	–	>10	–
**PN**	1.85 ± 0.15	–	5.13 ± 0.81	–

^a^ Selectivity Index: SI_1_ = IC_50_ Vero (Table 2)/IC_50_ Clone 15 HL‐60.

^b^ Selectivity index: SI_2_ = IC_50_ Vero (Table 2)/IC_50_ A375; Clone 15 HL‐60: Human promyelocytic leukemia cells; A375: malignant melanoma cells; PN: Parthenolide. All reported anticancer activity IC_50_s data were significant at *p* < .05.

The synthesized intermediates **1a‐1l** exhibited no qualifying growth inhibition, hence and were not shown in Table 4. Similarly, most analogues apart from **2h** did not show appreciable growth inhibition of A375 cells. Compound **2h** had an IC_50_ of 8.05 ± 0.57 μM, but its moderate cytotoxicity resulted in a SI of 2, indicative of its non‐intrinsic activity, disqualifying it as potential antimelanoma hit. However, this analogue was identified a possible hit compound against acute promyelocytic leukemia as it exhibited IC_50_ values below 10 μM paired with the recommended selectivity in the appropriate range (SI > 10). Analogue **2h** demonstrated a marginal twofold and a significant ninefold higher potency than the standard partenolide and NFX, respectively. The latter nitrofuran antibiotic was also confirmed as antileukemic hit. Compounds **2b**, **2d**, and **2f** alongside the precursor NFA had IC_50_ values below 10 μM against HL‐60; however, their moderate toxicity resulted in poor SI values.

Overall, the disparity in biological activities among the analogues did not allow for the deduction of structure–activity relationship (SAR). Possible contributions to this disparity may include compound solubility and/or membrane permeability issues. Furthermore, choice of host cell used during anti‐amastigote cultures may also contribute to disparities and/or low activities. As illustrated by Franco et al. (2019), the choice of cell line as host cell can significantly affect the infectivity and drug responses of intracellular parasite assays. Moreover, they have shown that THP1 cells, in particular, provide lower drug responses compared to other cell types and this may be attributed to the hostile cellular physiology of a macrophage interfering with the pharmacodynamics of compounds.

## 4 CONCLUSION

A series of nitrofuranyl sulfonohydrazide analogues of 5‐nitro‐2‐furaldehyde were synthesized in good yields in a two‐step process resulting in sulfonohydrazide intermediates and final Schiff bases. These compounds were found to be moderately toxic with disparate activity profiles; hence, no outright SAR could be deduced from the study. Analogues **2d** and **2e**, **2f**, **2h**, and **2l** presented with micromolar anti‐amastigote activities against *L. major* and *L. donovani* species. These compounds had good selectivity indices (SI > 10) relative to the host macrophages, but were found to possess moderate general cytotoxicity, which disqualified them as potential antileishmanial hits. Hence, this study did not unravel any antileishmanial hit. However, it uncovered analogue **2h** as hit against acute promyelocytic leukemia cancer owing to its good activity (IC_50_ < 10 μM) and selectivity profile (SI > 10). Hence, this research revealed no dual active hit with potential to treat leishmaniasis‐cancer comorbidities.

## CONFLICT OF INTEREST

The authors declare that they have no conflict of interest.

## Supporting information


Appendix S1

## Data Availability

All data generated or analyzed during this study are included in this article and the attached supplementary information file.
