# Supplementary material for: Exploring novel nitrofuranyl sulfonohydrazides as anti‐Leishmania and anti‐cancer agents: Synthesis, in vitro efficacy and hit identification
Source: Chem Biol Drug Des. 2022 Jun 21;100(2):267–79. doi: 10.1111/cbdd.14097 (PMC9546217; doi:10.1111/cbdd.14097)
Supplement: Supplementary file 1 — Appendix S1 [file CBDD-100-267-s001.docx]

**Supplementary Information**

**Exploring novel nitrofuranyl sulfonohydrazides as anti-*Leishmania* and - cancer agents: Synthesis, *in vitro* efficacy and hit identification**

Christina Kannigadu^1^, Janine Aucamp^1^, David D. N'Da^1^*

1 Centre of Excellence for Pharmaceutical Sciences, North-West University, Potchefstroom 2520, South Africa

*Correspondence:

Prof David D. N’Da, Centre of Excellence for Pharmaceutical Sciences, North-West University, Potchefstroom 2520, South Africa

Email: David.nda@nwu.ac.za; ORCID ID: 0000-0002-2327-0551

|  | **Page no.** |
| --- | --- |
| Chemistry………………………………………………………………………………... | 3 |
| 4-Iodobenzenesulfonohydrazide (**1a**), ^1^H NMR, ^13^C NMR, IR…………………………. | 4-5 |
| 4-Nitrobenzenesulfonohydrazide **(1b)**, ^1^H NMR, ^13^C NMR, IR………………………… | 6-7 |
| 4-Methylbenzenesulfonohydrazide (**1c**), ^1^H NMR, ^13^C NMR, IR………………………. | 8-9 |
| 4-Isopropylbenzenesulfonohydrazide **(1d)**, ^1^H NMR, ^13^C NMR, IR……………………. | 10-11 |
| 2,4,6-Trimethylbenzenesulfonohydrazide (**1e**), ^1^H NMR, ^13^C NMR, IR………………... | 12-13 |
| 4-(*tert*-Butyl)benzenesulfonohydrazide **(1f)**, ^1^H NMR, ^13^C NMR, IR…………………... | 14-15 |
| *N*-[4-(Hydrazinylsulfonyl)phenyl]acetamide **(1g)**, ^1^H NMR, ^13^C NMR, IR…………….. | 16-17 |
| (1,1'-Biphenyl)-4-sulfonohydrazide **(1h)**, ^1^H NMR, ^13^C NMR, IR……………………… | 18-19 |
| 4-Fluoro-(1,1'-biphenyl)-4-sulfonohydrazide **(1i)**, ^1^H NMR, ^13^C NMR, IR…………….. | 20-21 |
| 4-Chloro-(1,1'-biphenyl)-4-sulfonohydrazide **(1j)**, ^1^H NMR, ^13^C NMR, IR……………. | 22-23 |
| 4-Methoxy-(1,1'-biphenyl)-4-sulfonohydrazide **(1k)**, ^1^H NMR, ^13^C NMR, IR…………. | 24-25 |
| 2,3-Dihydrobenzo[*b*][1,4]dioxine-5-sulfonohydrazide **(1l)**, ^1^H NMR, ^13^C NMR, IR…... | 26-27 |
| *(E,Z)*-4-Iodo-*N'*-[(5-nitrofuran-2-yl)methylene]benzenesulfonohydrazide (**2a**), ^1^H NMR, ^13^C NMR, IR, HRMS…………………………………………………………….. | 28-30 |
| (*E,Z*)-4-Nitro-*N'*-[(5-nitrofuran-2-yl)methylene]benzenesulfonohydrazide **(2b)**, ^1^H NMR, ^13^C NMR, IR, HRMS…………………………………………………………….. | 31-33 |
| (*E,Z*)-4-Methyl-*N'*-[(5-nitrofuran-2-yl)methylene]benzenesulfonohydrazide **(2c)**, ^1^H NMR, ^13^C NMR, IR, HRMS…………………………………………………………….. | 34-36 |
| (*E,Z*)-4-Isopropyl-*N'*-[(5-nitrofuran-2-yl)methylene]benzenesulfonohydrazide (**2d**), ^1^H NMR, ^13^C NMR, IR, HRMS…………………………………………………………….. | 37-39 |
| (*E,Z*)-2,4,6-Trimethyl-*N*'-[(5-nitrofuran-2-yl)methylene]benzenesulfonohydrazide **(2e),** ^1^H NMR, ^13^C NMR, IR, HRMS ………………………………………………………… | 40-42 |
| *E,Z*)-4-(*tert*-Butyl)-*N'*-[(5-nitrofuran-2-yl)methylene]benzenesulfonohydrazide **(2f)**, ^1^H NMR, ^13^C NMR, IR, HRMS……………………………………………………………. | 43-45 |
| (*E,Z*)-*N*-{4-{{2-[(5-nitrofuran-2-yl)methylene]hydrazinyl}sulfonyl}phenyl}acetamide **(2g)**, ^1^H NMR, ^13^C NMR, IR, HRMS…………………………………………………… | 46-48 |
| (*E,Z*)-*N'*-[(5-Nitrofuran-2-yl)methylene]-(1,1'-biphenyl)-4-sulfonohydrazide **(2h)**, ^1^H NMR, ^13^C NMR, IR, HRMS……………………………………………………………. | 49-51 |
| *(E,Z)*-4-Fluoro-*N'*-([5-nitrofuran-2-yl]methylene)-(1,1'-biphenyl)-4-sulfonohydrazide **(2i)**, ^1^H NMR, ^13^C NMR, IR, HRMS……………………………………………………. | 52-54 |
| (*E,Z*)-4-Chloro-*N'*-[(5-nitrofuran-2-yl)methylene]-(1,1'-biphenyl)-4-sulfonohydrazide **(2j)**, ^1^H NMR, ^13^C NMR, IR, HRMS…………………………………………………… | 55-57 |
| (*E,Z*)-4-Methoxy-*N'*-[(5-nitrofuran-2-yl)methylene]-(1,1'-biphenyl)-4-sulfonohydrazide **(2k)**, ^1^H NMR, ^13^C NMR, IR, HRMS……………………………………………………. | 58-60 |
| (*E,Z*)-*N'*-[(5-Nitrofuran-2-yl)methylene]-2,3-dihydrobenzo[*b*][1,4]dioxine-5-sulfonohydrazide **(2l)**, ^1^H NMR, ^13^C NMR, IR, HRMS…………………………………. | 61-62 |
| *In vitro* biological assays………………………………………………………………… | 64-67 |
| References………………………………………………………………………………... | 67 |

**Table of Contents**

**Chemistry**

***Materials and general procedures***

All reagents were obtained from Sigma Aldrich (South Africa) and solvents were purchased from Associated Chemical Enterprises, ACE (South Africa). All chemicals and reagents were reagent/analytical grade and were used without further purification.

The ^1^H, and ^13^C nuclear magnetic resonance (NMR) spectra were recorded on a Bruker Advance™ III 600 spectrometer at a frequency of 600 and 150.913 MHz, respectively in DMSO-*d_6_*. Chemical shifts are reported in parts per million δ (ppm), with the residual protons of the solvent as reference. The splitting pattern abbreviations are as follows: singlet (s), doublet (d), doublet of doublet (dd), doublet of triplets (dt), triplet (t), triplet of doublets (td), triplet of triplets (tt), quartet of doublets (qd) and multiplet (m).

High resolution mass spectrometry (HRMS) was recorded on a Bruker MicroTOF Q II mass spectrometer, equipped with an APCI or ESI source, set at 200 or 180 °C, respectively, using Bruker Compass Data Analysis 4.0 software. A full scan from 50-1500 m/z was performed at a capillary voltage of 4500 V, an end plate offset voltage of -500 V, with the nebulizer set at 1.6 and 0.4 Bar, respectively, and a collision cell RF voltage of 100 Vpp.

Fourier Transformed Infrared (FTIR) spectroscopy spectra were recorded on a Bruker Alpha-P FTIR instrument. Thin layer chromatography (TLC) was performed, using silica gel plates (60F254) obtained from Merck (Johannesburg, South Africa), to monitor the progress of reactions and visualization of spots was done using UV-light (254 nm) and iodine.

Melting points (mp) were determined with a Büchi melting point B-545 instrument and were uncorrected.

**General procedure for the synthesis of sulfonyl hydrazide derivatives (1a-l)**

Substituted sulfonyl chloride (52.0 mmol; 1.0 g, 1 eq.) was dissolved in dichloromethane (4 mL). To the reaction mixture, triethylamine (58.4 mmol, 0.83 mL, 1.5 eq.) was slowly added dropwise over a 15-minute period. Thereafter, hydrazine hydrate (60% solution) (0.27 mL, 10 eq.) was added dropwise. The reaction was stirred at room temperature for 2 hours and monitored with TLC. Upon completion of the reaction, water was added to the reaction and the precipitate was filtered off. The precipitate was recrystallized in ethyl acetate/hexane (1:9, v/v) to afford a white solid. The structures of the synthesized compounds were verified by NMR spectroscopy. The data and spectra are reported in Supplementary Information for each intermediate.

**COMPOUNDS**

**4-Iodobenzenesulfonohydrazide (1a)**

**^1^H NMR in DMSO**


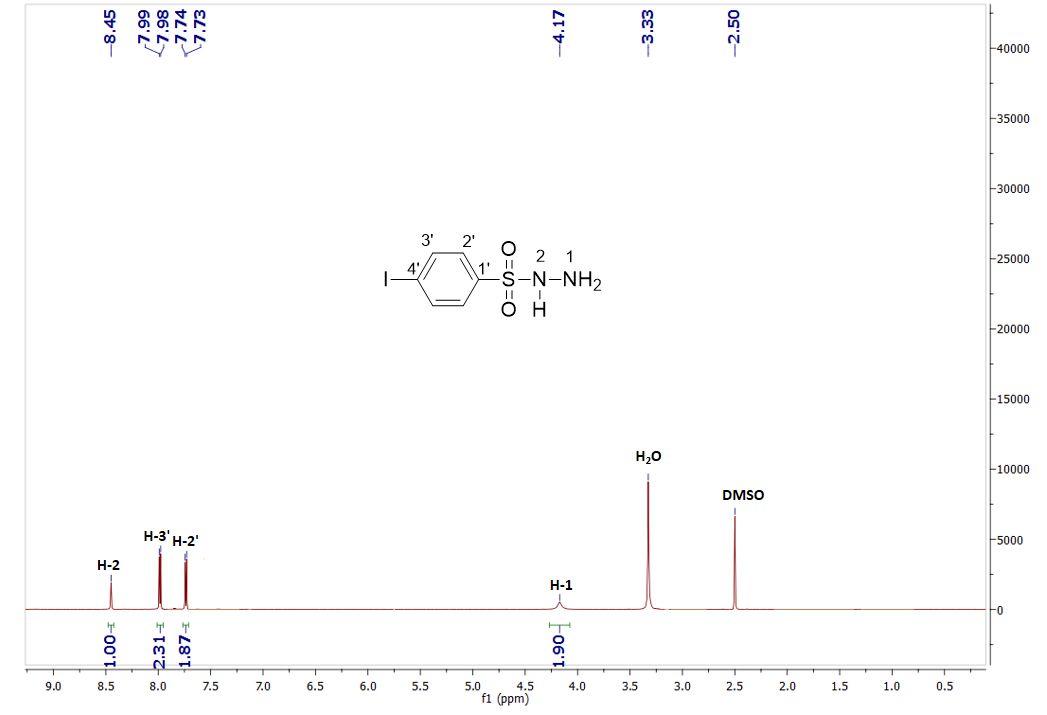


**^13^C NMR in DMSO**

**IR Spectrum**


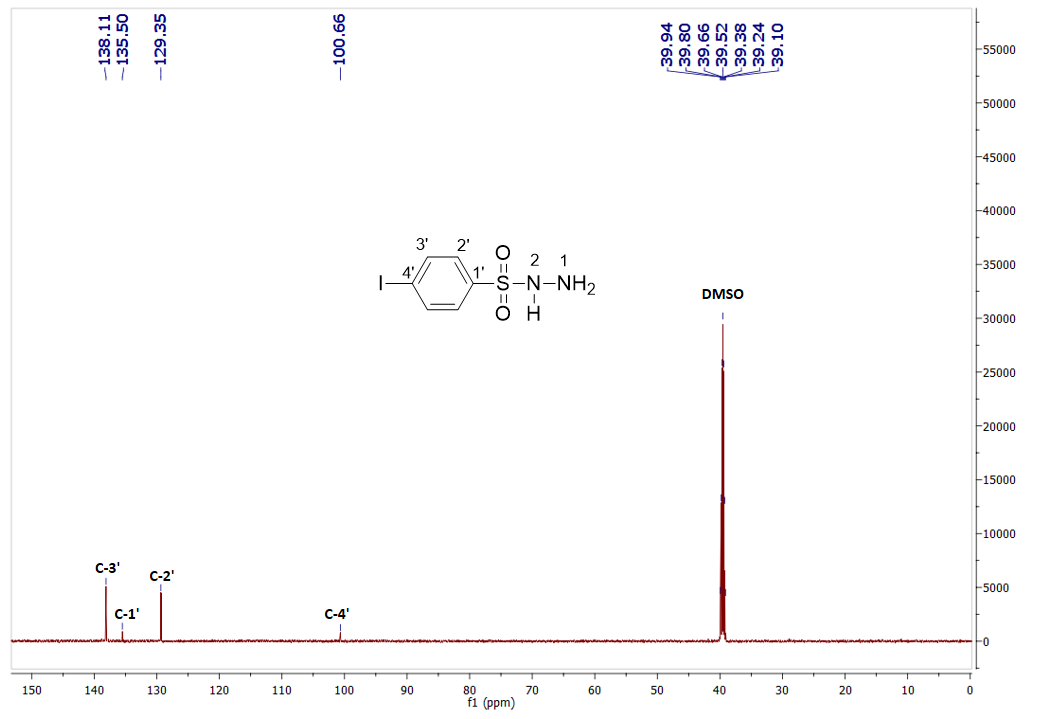


**
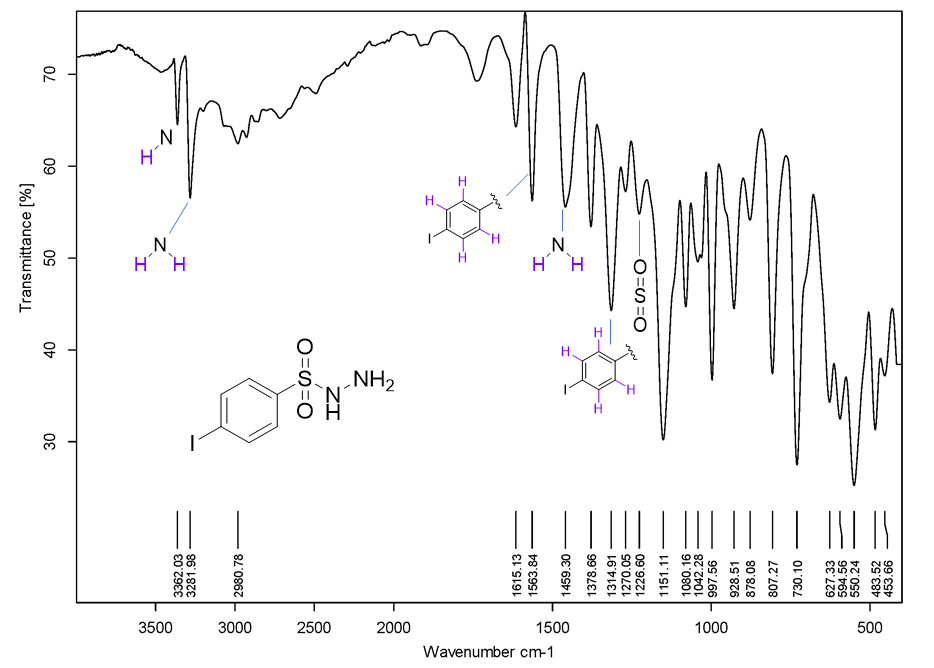
**

White solid, yield: 90%; mp: 160-162 °C, R*_f_* = 0.40 (EtOAc:*n*-Hex, 1:1, v/v), IR υ_max_ (cm^-1^): 3362 (N-H), 3281 (NH_2_), 1563 (Ar-H), 1459 (NH_2_), 1314 (Ar-H), 1226 (S=O). ^1^H NMR (600 MHz, DMSO) δ 8.45 (s, 1H, H-2), 7.98 (d, *J* = 8.5 Hz, 2H, H-3'), 7.74 (d, *J* = 8.5 Hz, 2H, H-2'), 4.17 (s, 2H, H-1). ^13^C NMR (151 MHz, DMSO) δ 138.11 (C-3'), 135.50 (C-1'), 129.35 (C-2'), 100.66 (C-4').

**4-Nitrobenzenesulfonohydrazide (1b)**

**^1^H NMR in DMSO**


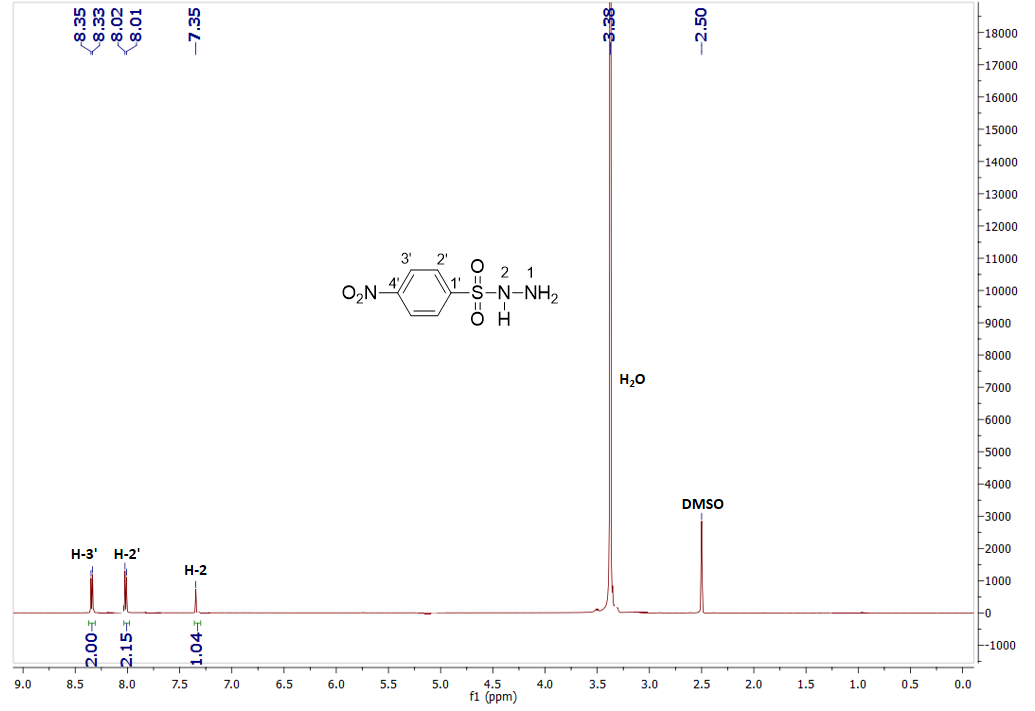


**^13^C NMR in DMSO**


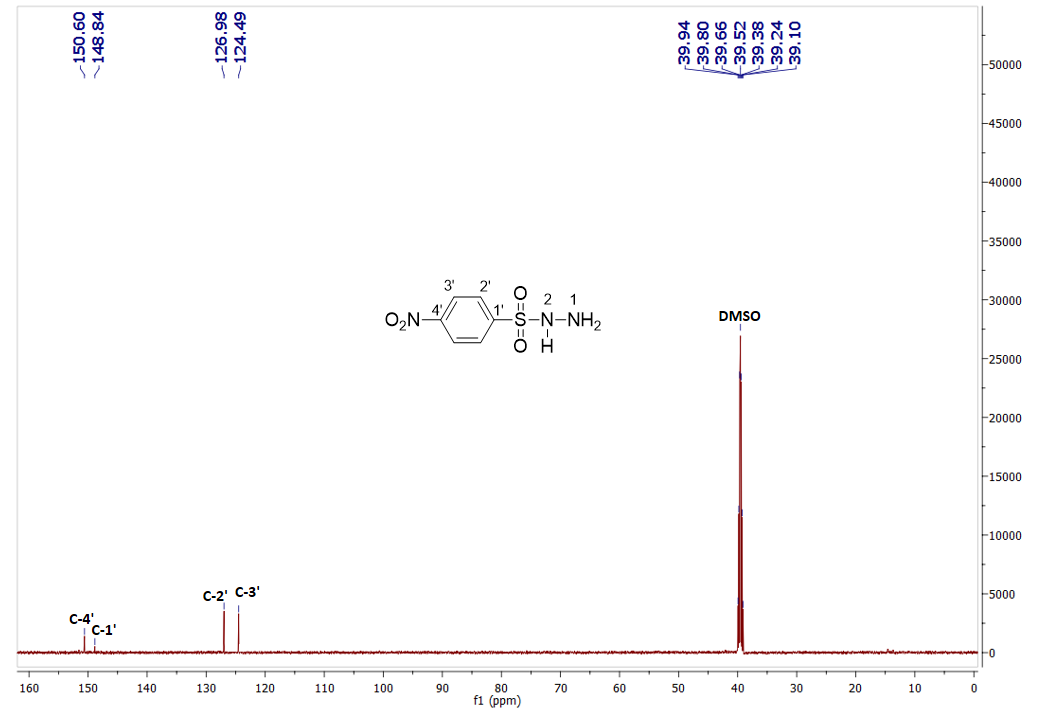


**IR Spectrum**


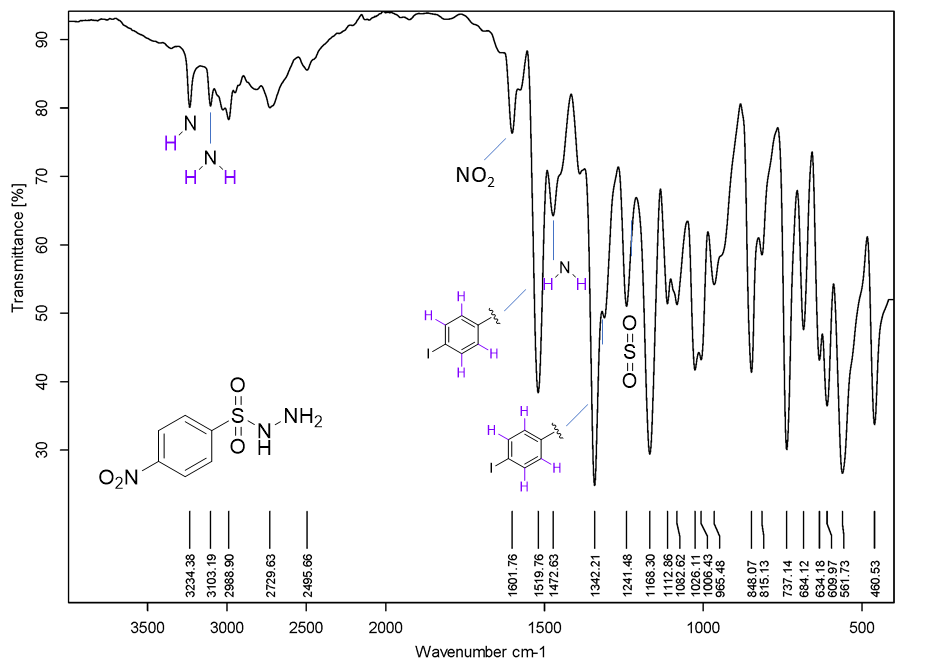


Orange solid, yield: 80%, mp: 149-152 °C, R*_f_* = 0.50 (EtOAc:*n*-Hex, 1:1, v/v), IR υ_max_ (cm^-1^): 3234 (N-H), 3103 (NH_2_), 1601 (NO_2_), 1519 (Ar-H), 1472 (NH_2_), 1342 (Ar-H), 1241 (S=O). ^1^H NMR (600 MHz, DMSO) δ 8.34 (d, *J* = 8.8 Hz, 1H, H-3'), 8.02 (d, *J* = 8.9 Hz, 1H, H-2'), 7.35 (s, 1H, H-2), *4.10 (s, 2H, H-1). ^13^C NMR (151 MHz, DMSO) δ 150.60 (C-4'), 148.84 (C-1'), 126.98 (C-2'), 124.49 (C-3').

*, signal could not be detected

**4-Methylbenzenesulfonohydrazide (1c)**

**^1^H NMR in DMSO**


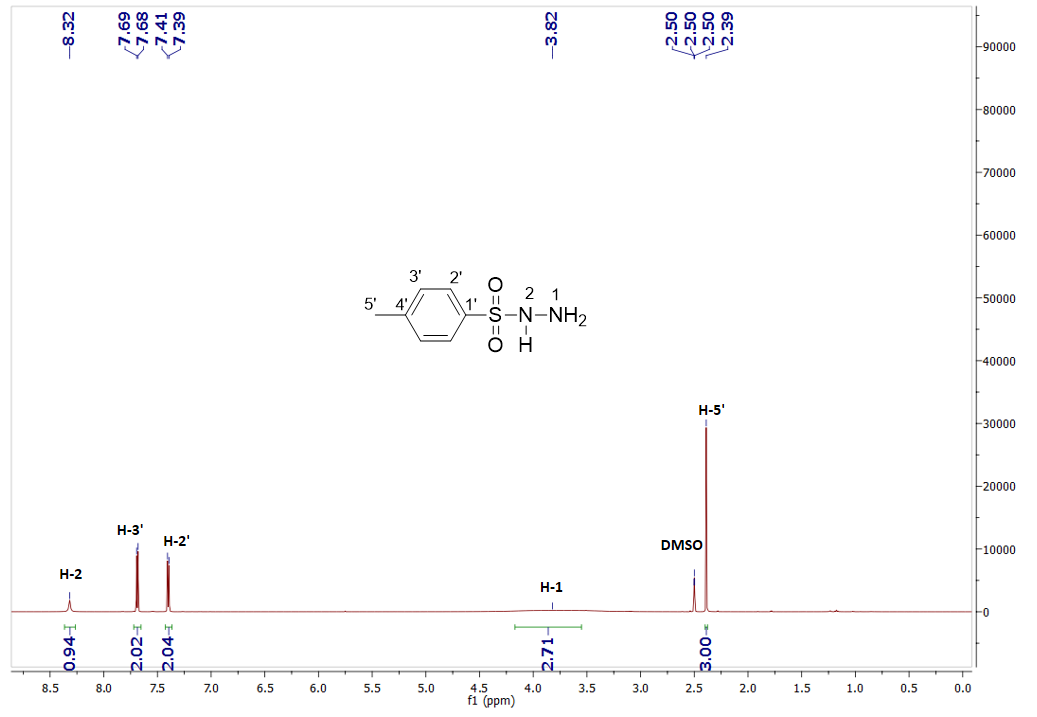


**^13^C NMR in DMSO**


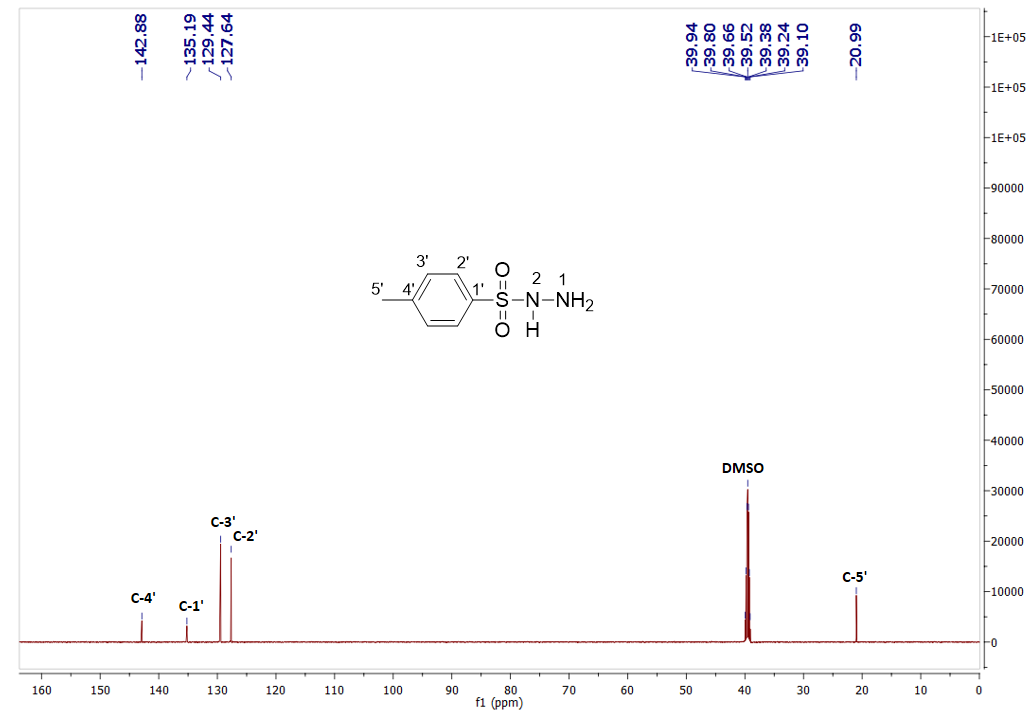


**IR Spectrum**


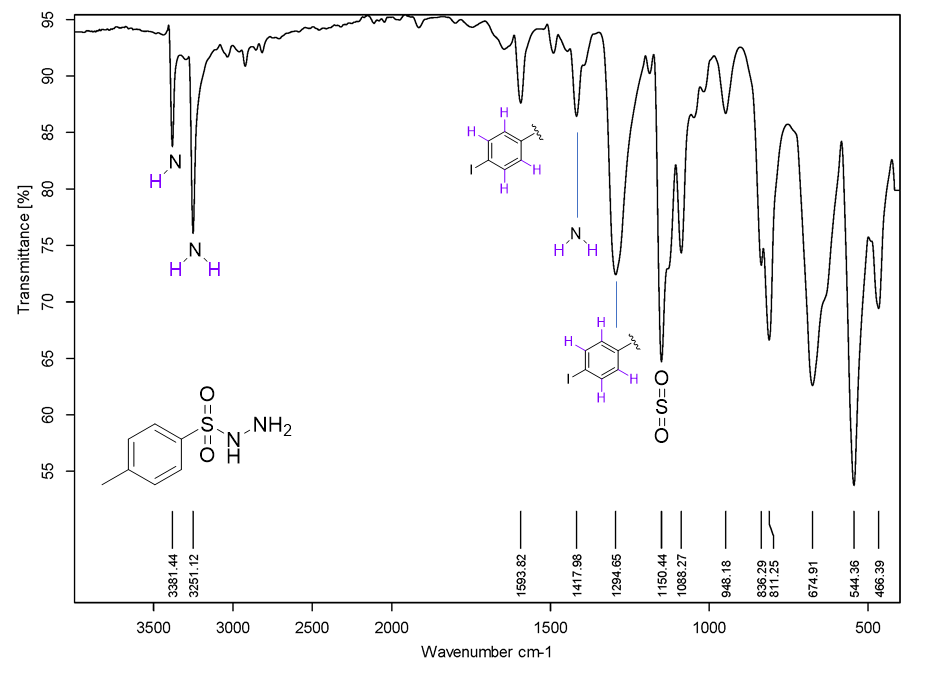


White solid, yield: 92%, mp: 170-173 °C, R*_f_* = 0.30 (EtOAc:*n*-Hex, 1:1, v/v), IR υ_max_ (cm^-1^): 3381 (N-H), 3251 (NH_2_), 1593 (Ar-H), 1417 (NH_2_), 1294 (Ar-H), 1150 (S=O). ^1^H NMR (600 MHz, DMSO) δ 8.32 (s, 1H, H-2), 7.69 (d, *J* = 8.2 Hz, 2H, H-3'), 7.40 (d, *J* = 8.2 Hz, 2H, H-2'), 3.82 (s, 2H, H-1), 2.39 (s, 3H, H-5'). ^13^C NMR (151 MHz, DMSO) δ 142.88 (C-4'), 135.19 (C-1'), 129.44 (C-3'), 127.64 (C-2'), 20.99 (C-5').

**4-Isopropylbenzenesulfonohydrazide (1d)**

**^1^H NMR in DMSO**


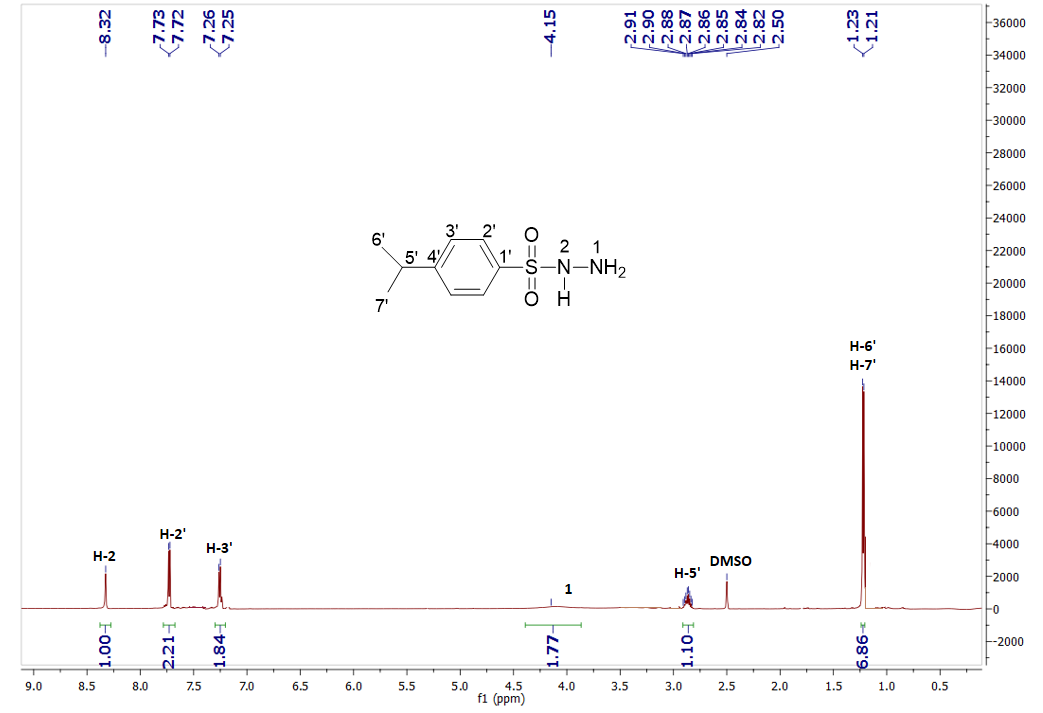


**^13^C NMR in DMSO**


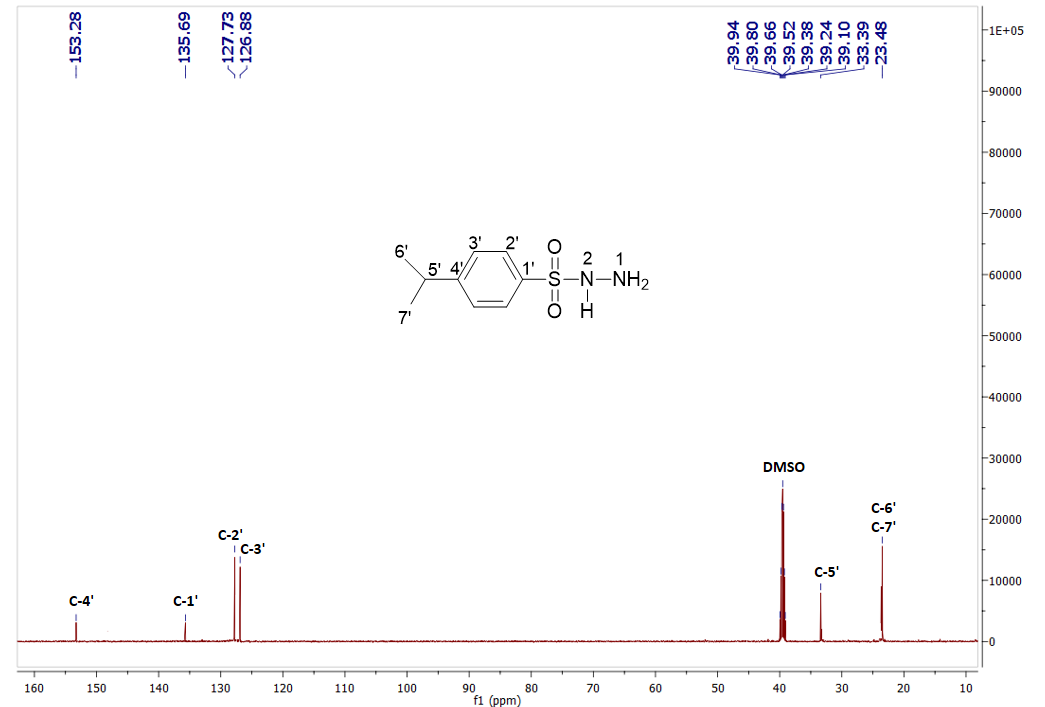


**IR Spectrum**


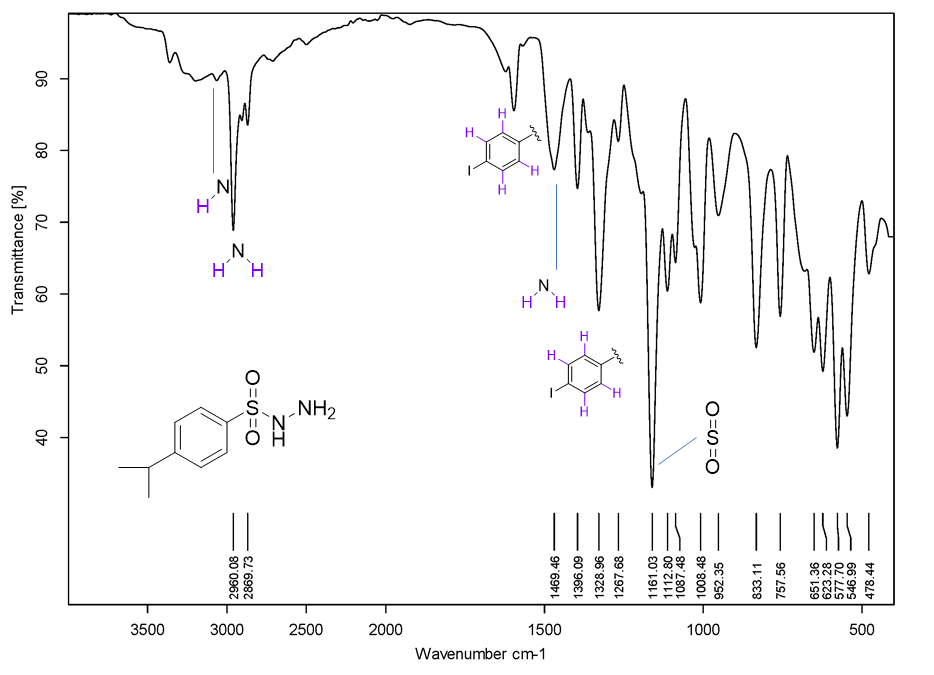


White solid, yield: 88%, R*_f_* = 0.44 (EtOAc:*n*-Hex, 1:1, v/v), IR υ_max_ (cm^-1^): 3251 (N-H), 2960 (NH_2_), 1580 (Ar-H), 1469 (NH_2_), 1328 (Ar-H), 1161 (S=O). ^1^H NMR (600 MHz, DMSO) δ 8.32 (s, 1H, H-2), 7.73 (d, *J* = 8.0 Hz, 2H, H-2'), 7.26 (d, *J* = 8.0 Hz, 2H, H-3'), 4.10 (s, 2H, H-1), 2.87 (tq, *J* = 14.0, 7.1 Hz, 1H, H-5'), 1.22 (d, *J* = 6.9 Hz, 6H, H-6'/7'). ^13^C NMR (151 MHz, DMSO) δ 153.28 (C-4'), 135.69 (C-1'), 127.73 (C-2'), 126.88 (C-3'), 33.39 (C-5'), 23.48 (C-6'/7').

**2,4,6-Trimethylbenzenesulfonohydrazide (1e)**

**^1^H NMR in DMSO**


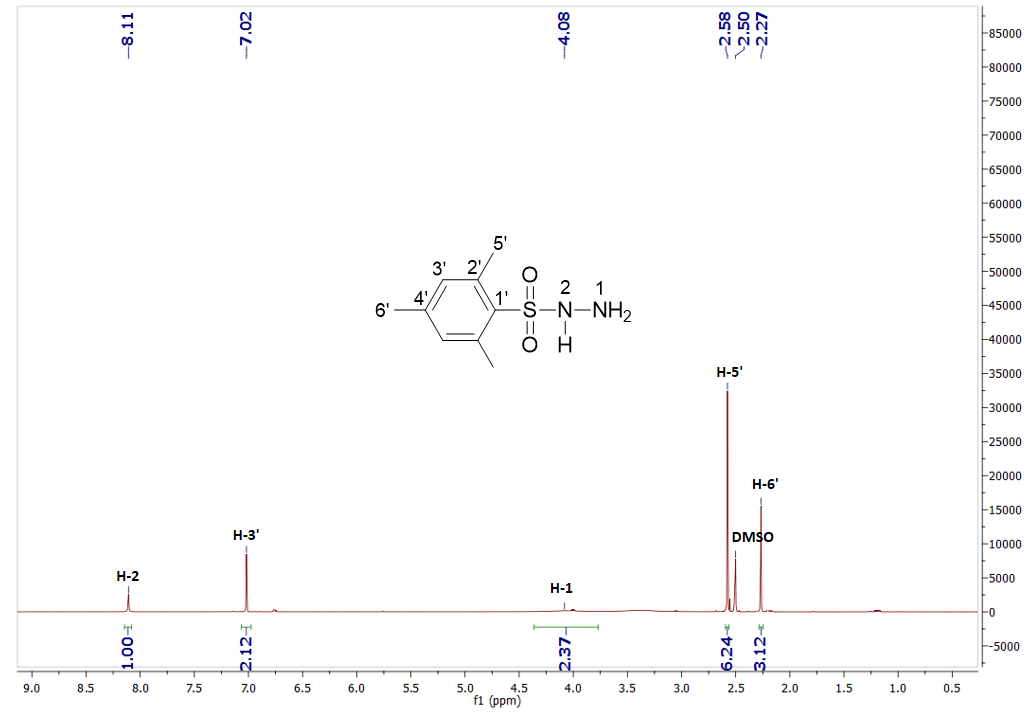


**^13^C NMR in DMSO**


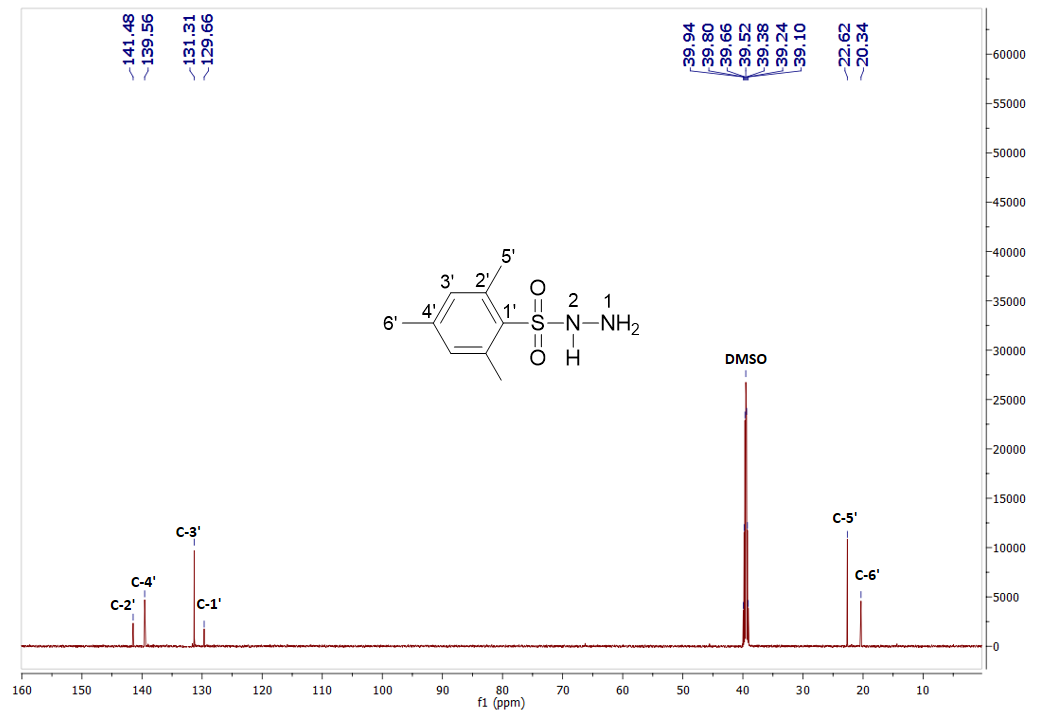


**IR Spectrum**

**
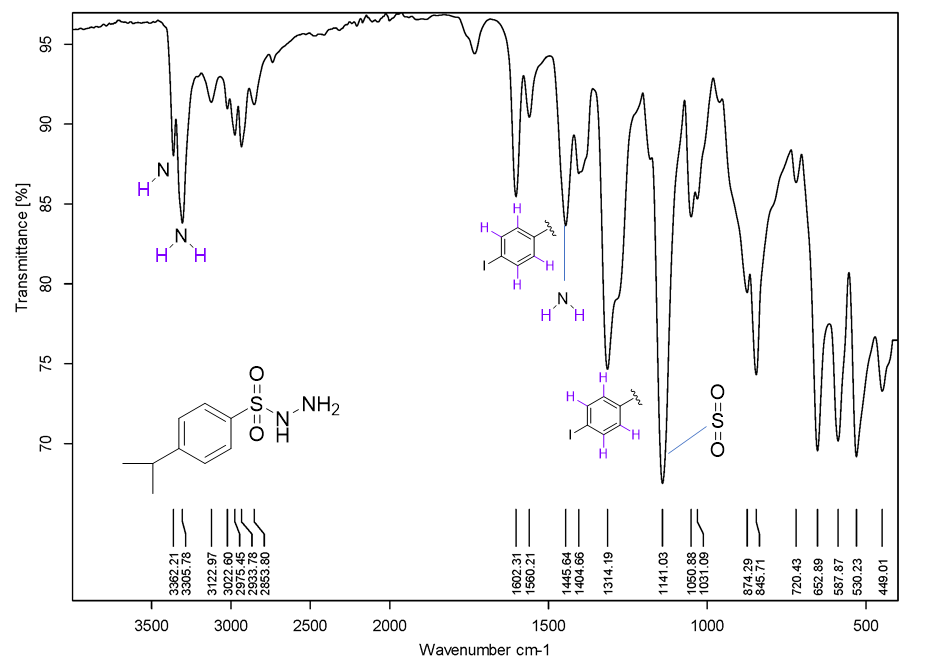
**

White solid, yield: 95%, mp: 110-113 °C, R*_f_* = 0.54 (EtOAc:*n*-Hex, 1:1, v/v), IR υ_max_ (cm^-1^): 3362 (N-H), 3305 (NH_2_), 1602 (Ar-H), 1445 (NH_2_), 1314 (Ar-H), 1141 (S=O).^1^H NMR (600 MHz, DMSO) δ 8.11 (s, 1H, H-2), 7.02 (s, 2H, H-3'), 4.08 (s, 2H, H-1), 2.58 (s, 6H, H-5'), 2.27 (s, 3H, H-6'). ^13^C NMR (151 MHz, DMSO) δ 141.48 (C-2'), 139.56 (C-4'), 131.31 (C-3'), 129.66 (C-1'), 22.62 (C-5'), 20.34 (C-6').

**4-(*tert*-Butyl)benzenesulfonohydrazide (1f)**

**^1^H NMR in DMSO**


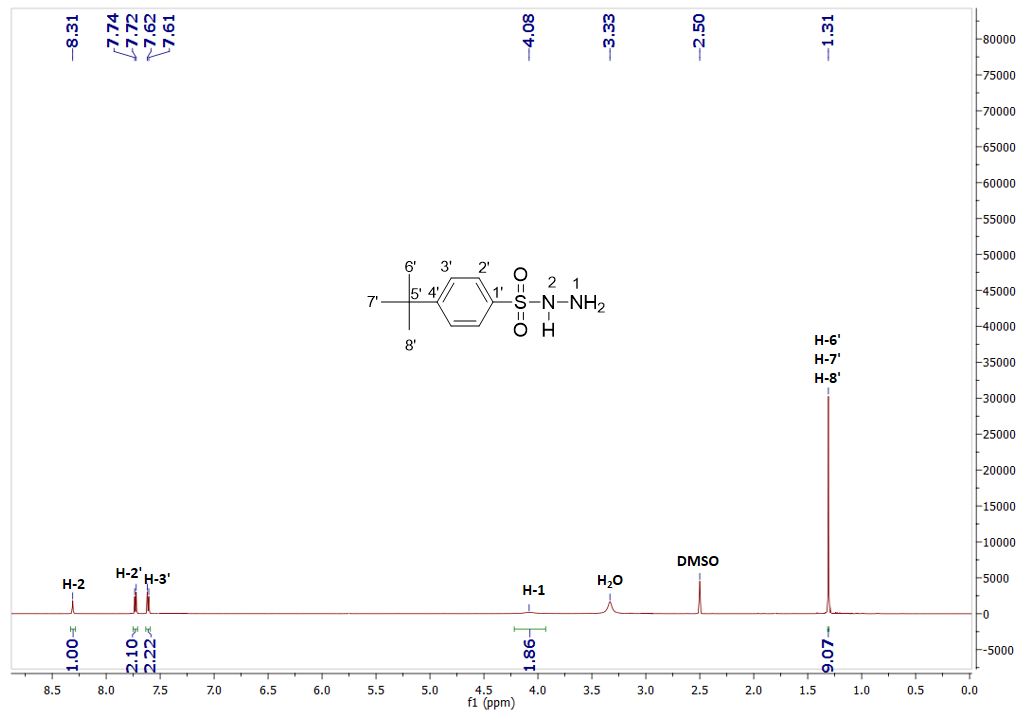


**^13^C NMR in DMSO**


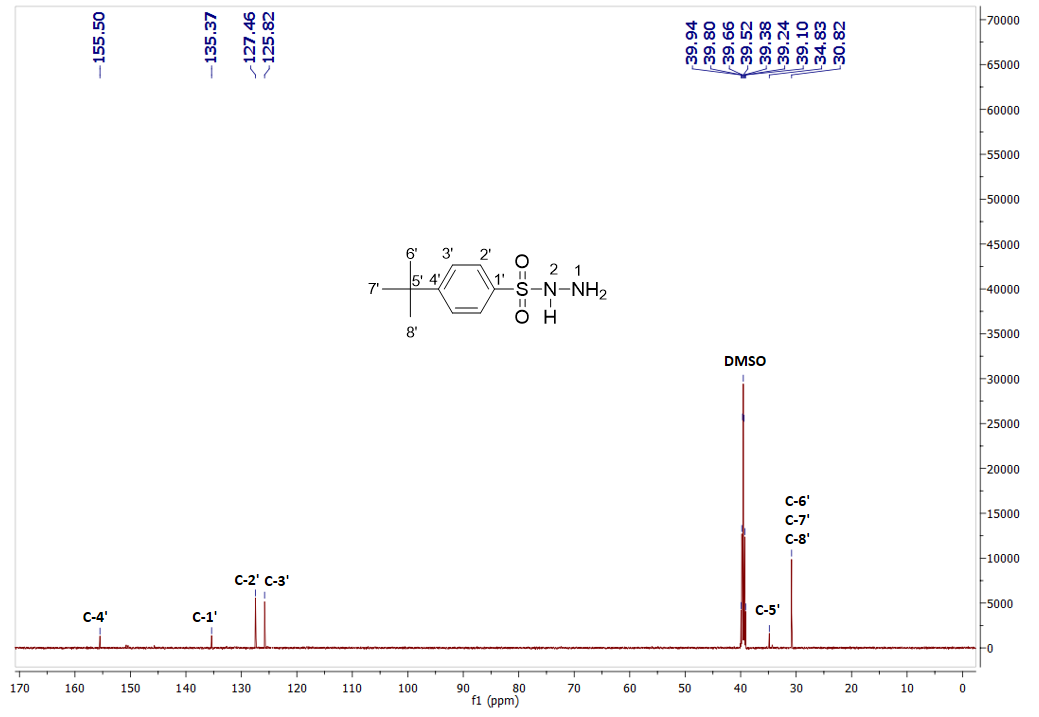


**IR Spectrum**

**
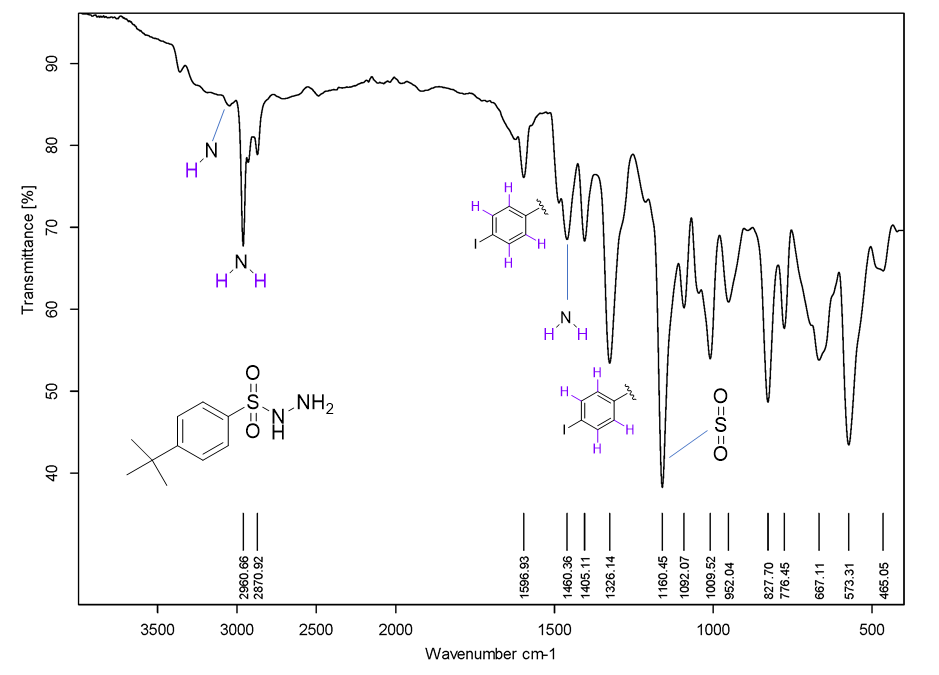
**

White solid, yield: 90%, IR υ_max_ (cm^-1^): 3369 (N-H), 2960 (NH_2_), 1596 (Ar-H), 1460 (NH_2_), 1326 (Ar-H), 1160 (S=O). ^1^H NMR (600 MHz, DMSO) δ 8.31 (s, 1H, H-2), 7.73 (d, *J* = 8.4 Hz, 2H, H-2'), 7.61 (d, *J* = 8.4 Hz, 2H, H-3'), 4.08 (s, 1H, H-1), 1.31 (s, 9H, H-6'/7'/8'). ^13^C NMR (151 MHz, DMSO) δ 155.50 (C-4'), 135.37 (C-1'), 127.46 (C-2'), 125.82 (C-3'), 34.83 (C-5'), 30.82 (C-6'/7'/8').

***N*-[4-(Hydrazinylsulfonyl)phenyl]acetamide (1g)**

**^1^H NMR in DMSO**


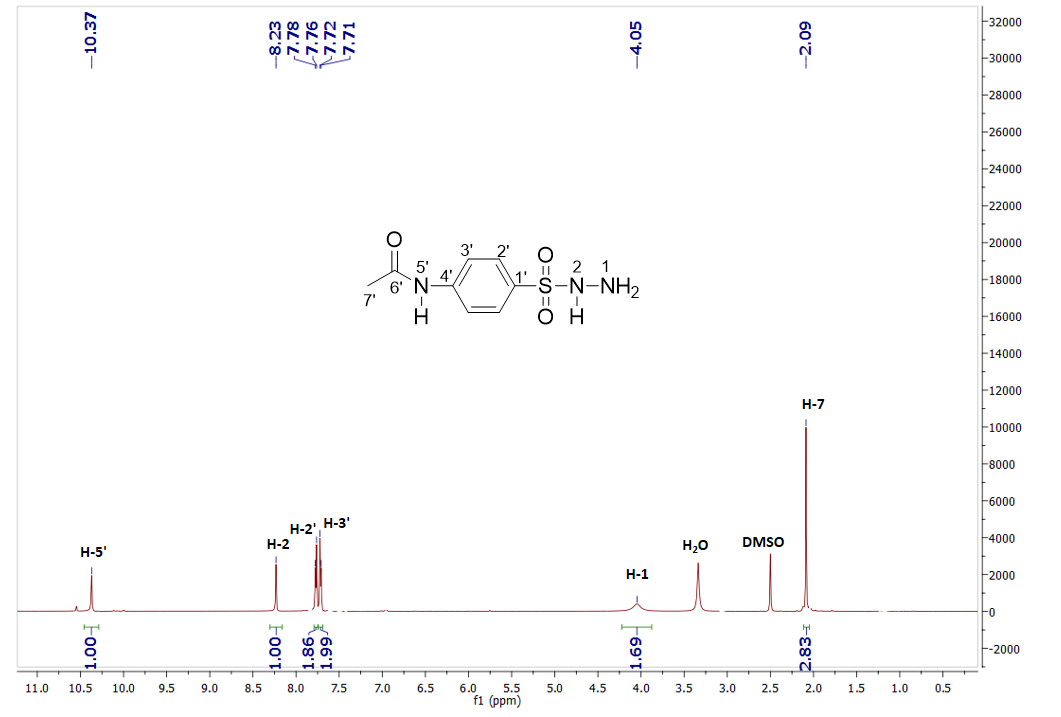


**^13^C NMR in DMSO**


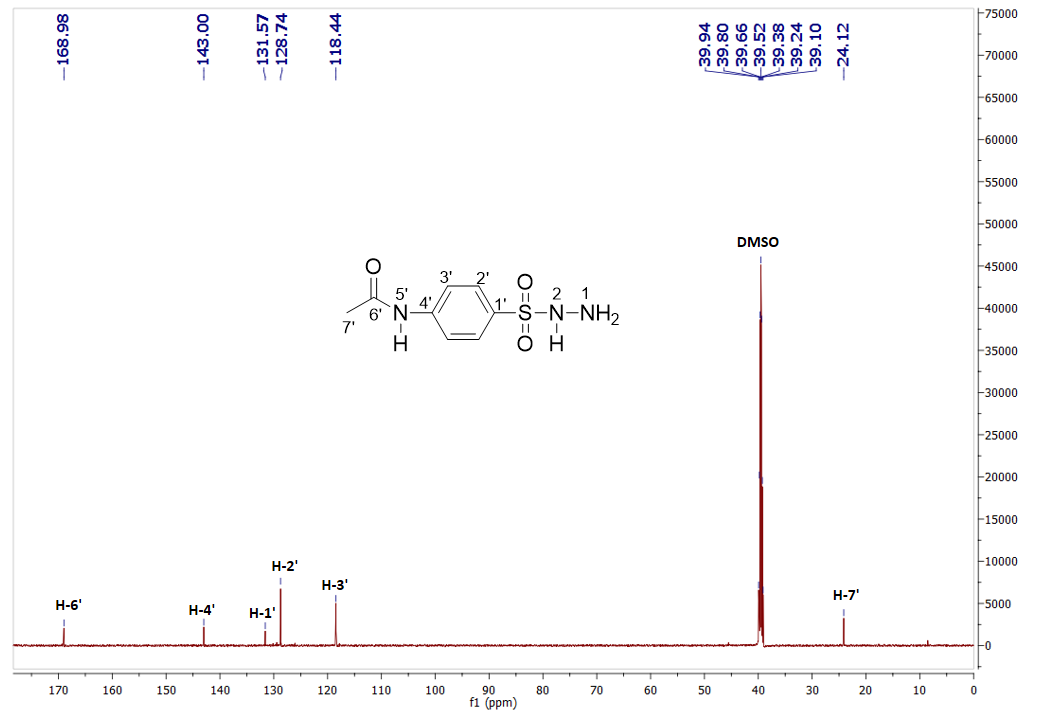


**IR Spectrum**

**
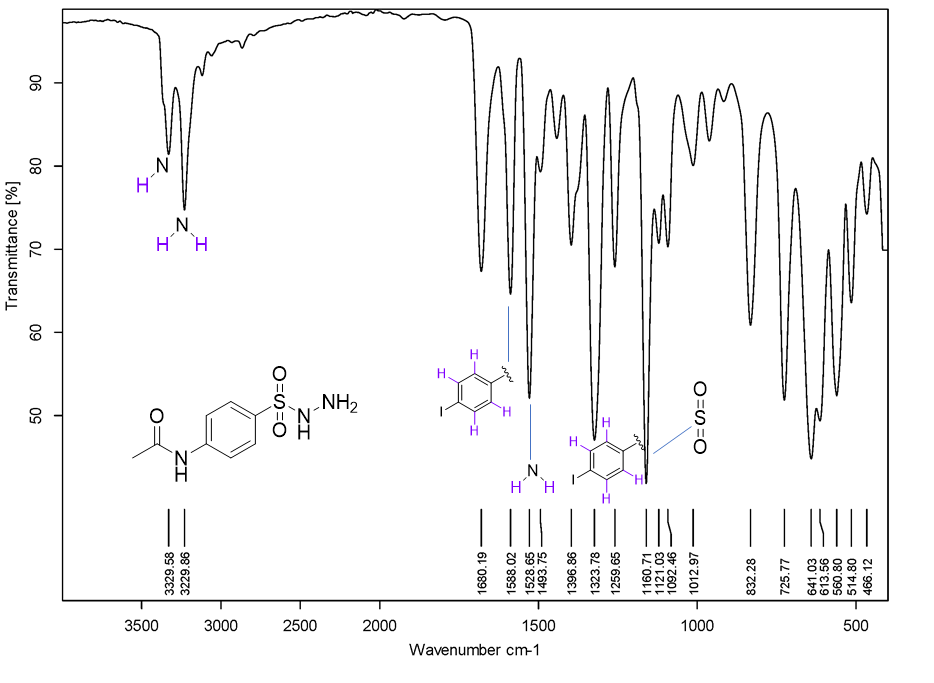
**

White solid, yield: 95%, mp:175-178 °C, R*_f_* = 0.50 (EtOAc:*n*-Hex, 1:1, v/v), IR υ_max_ (cm^-1^): 3329 (N-H), 3229 (NH_2_), 1588 (Ar-H), 1528 (NH_2_), 1396 (Ar-H), 1160 (S=O). ^1^H NMR (600 MHz, DMSO) δ 10.37 (s, 1H, H-5'), 8.23 (s, 1H, H-2), 7.77 (d, *J* = 8.5 Hz, 2H, H-2'), 7.72 (d, *J* = 8.5 Hz, 2H, H-3'), 4.05 (s, 2H, H-1), 2.09 (s, 3H, H-7'). ^13^C NMR (151 MHz, DMSO) δ 168.98 (C-6'), 143.00 (C-4'), 131.57 (C-1'), 128.74 (C-2'), 118.44 (C-3'), 24.12 (C-7').

**(1,1'-Biphenyl)-4-sulfonohydrazide (1h)**

**^1^H NMR in DMSO**


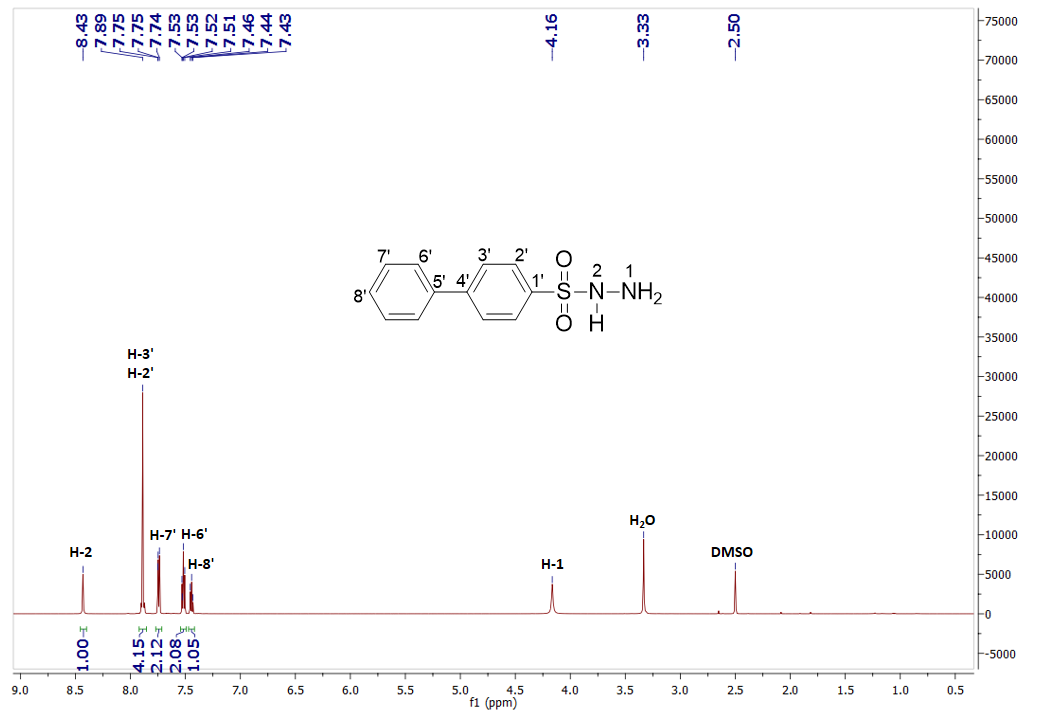


**^13^C NMR in DMSO**


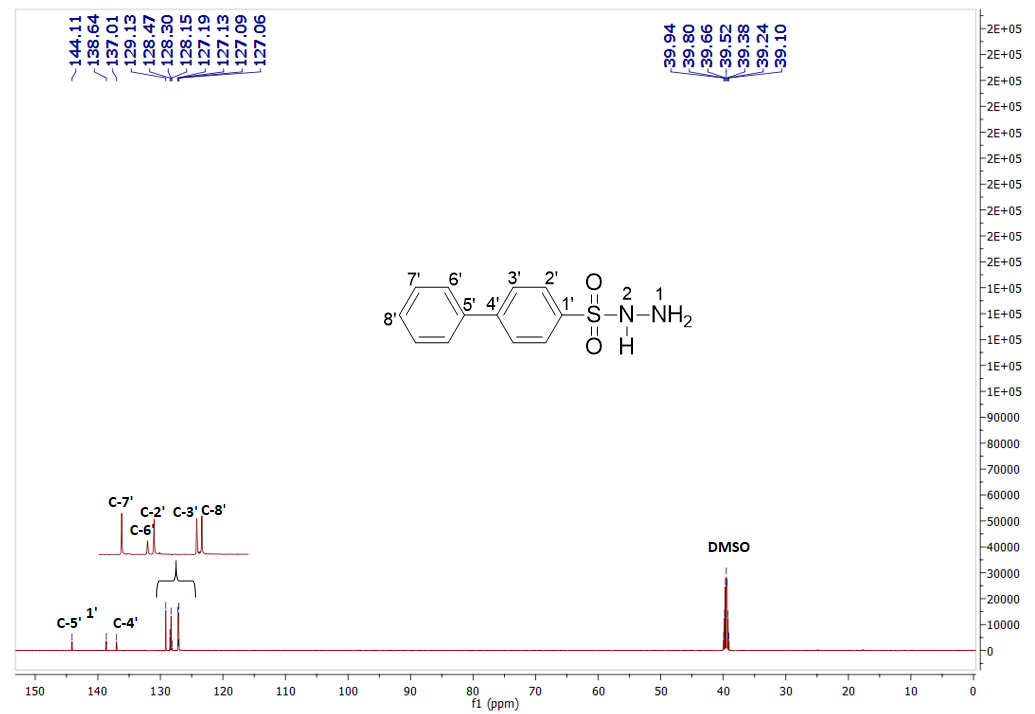


**IR Spectrum**

**
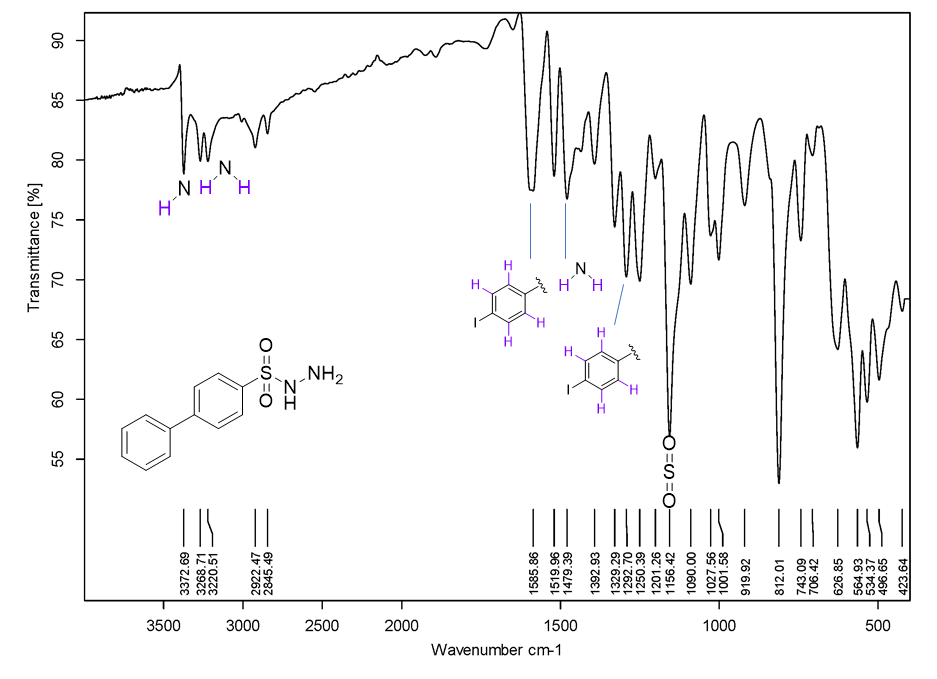
**

White solid, yield: 98%, mp: 180-183 °C, R*_f_* = 0.38 (EtOAc:*n*-Hex, 1:1, v/v), IR υ_max_ (cm^-1^): 3372 (N-H), 3268 (NH2), 1585 (Ar-H), 1479 (NH2), 1329 (Ar-H), 1156 (S=O).^1^H NMR (600 MHz, DMSO) δ 8.43 (s, 1H, H-2), 7.89 (s, 4H, H-3'/2'), 7.74 (d, *J* = 7.4 Hz, 2H, H-7'), 7.52 (t, *J* = 7.4 Hz, 2H, H-6'), 7.44 (t, *J* = 7.4 Hz, 1H, H-8), 4.16 (s, 2H, H-1). ^13^C NMR (151 MHz, DMSO) δ 144.11 (C-5'), 138.64 (C-1'), 137.01 (C-4'), 129.13 (C-7'), 128.47 (C-6'), 128.30 (C-2'), 127.19 (C-8'), 127.06 (C-3').

**4-Fluoro-(1,1'-biphenyl)-4-sulfonohydrazide (1i)**

**^1^H NMR in DMSO**


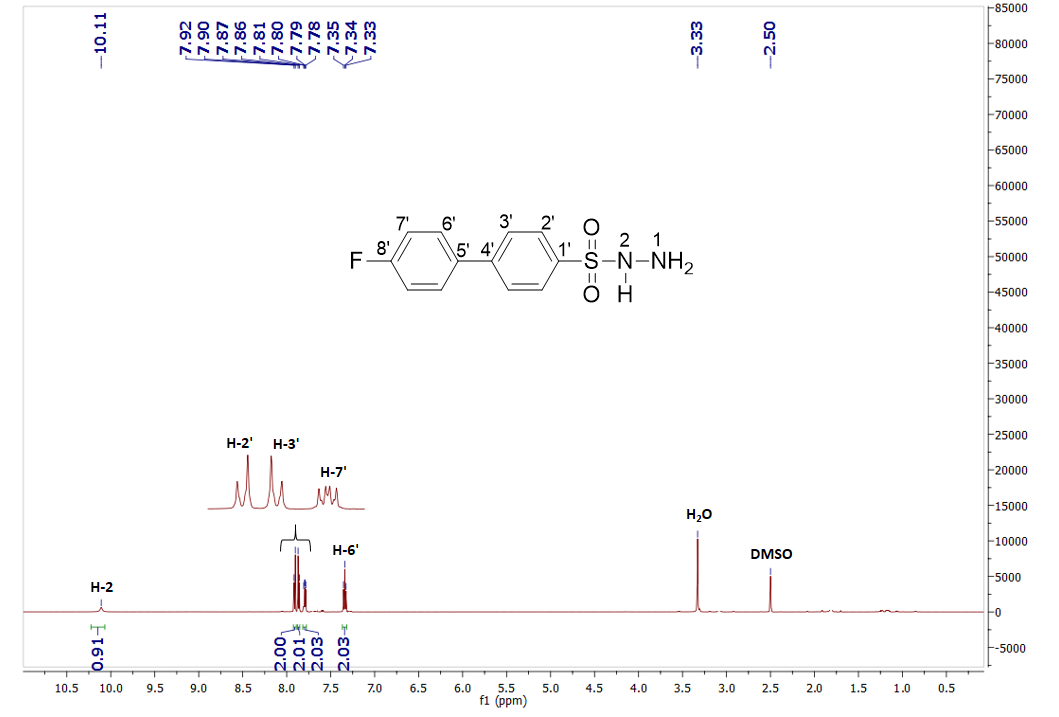


**^13^C NMR in DMSO**


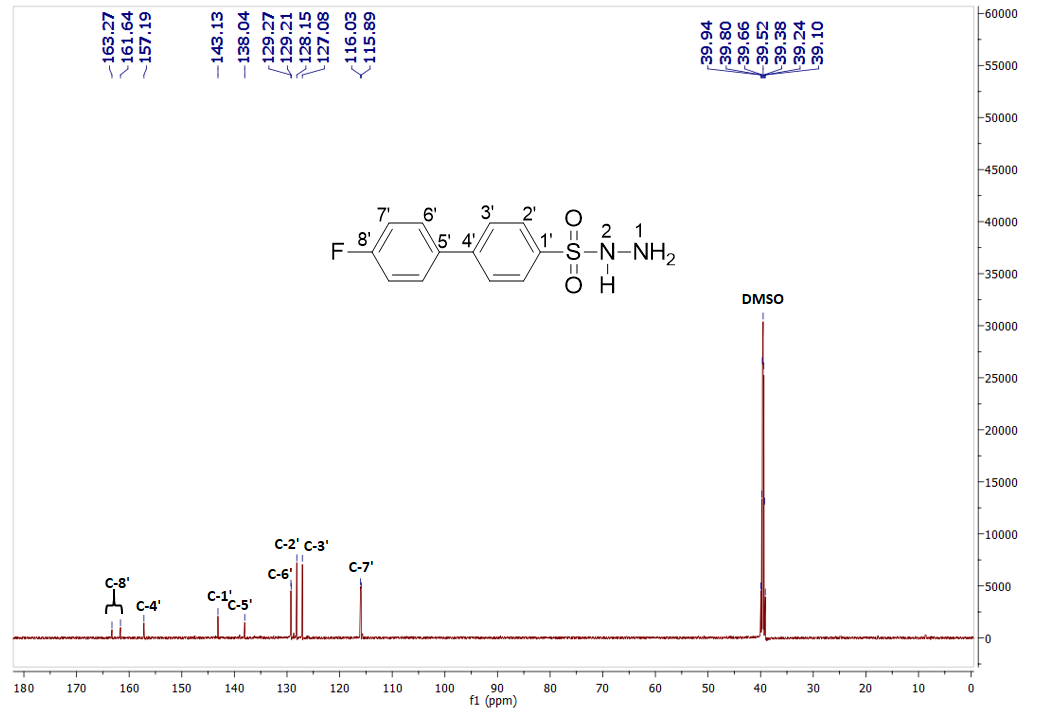


**IR Spectrum**

**
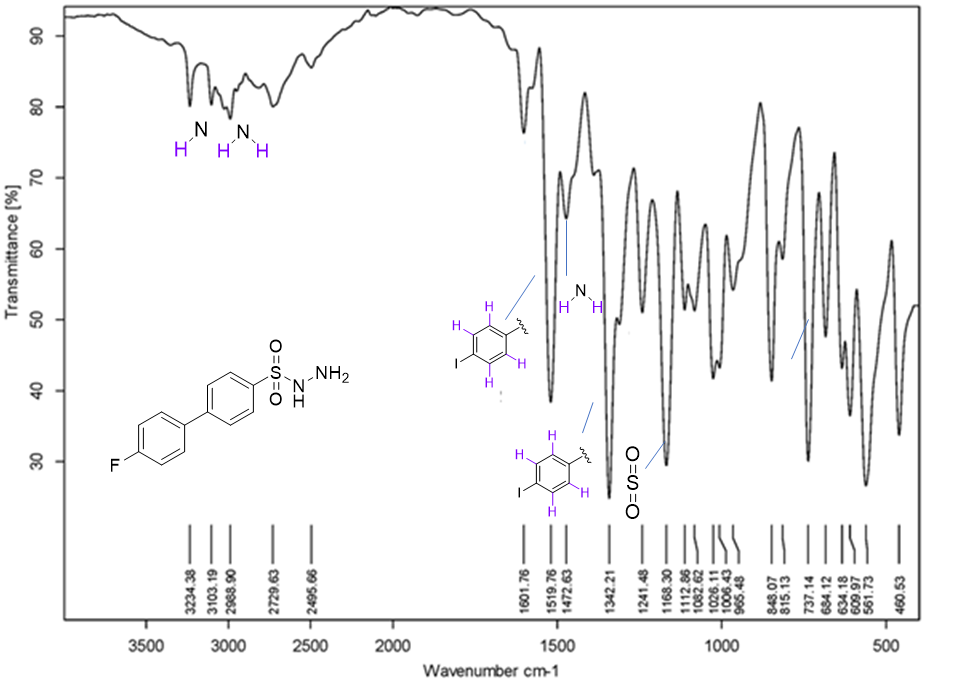
**

White solid, yield: 96%, mp: 164-169 °C, R*_f_* = 0.60 (EtOAc:*n*-Hex, 1:1, v/v), IR υ_max_ (cm^-1^): 3232 (N-H), 3103 (NH_2_), 1519 (Ar-H), 1472 (NH_2_), 1342 (Ar-H), 1168 (S=O).^1^H NMR (600 MHz, DMSO) δ 10.11 (s, 1H, H-2), 7.91 (d, *J* = 8.5 Hz, 2H, H-2'), 7.86 (d, *J* = 8.5 Hz, 2H, H-3'), 7.79 (dd, *J* = 8.8, 5.4 Hz, 2H, H-7'), 7.34 (t, *J* = 8.8 Hz, 2H, H-6'), *4.10 (s, 2H, H-1).^13^C NMR (151 MHz, DMSO) δ 162.46 (d, *J* = 245.8 Hz, C-8'), 157.19 (C-4'), 143.13 (C-1'), 138.04 (C-5'), 129.24 (d, *J* = 8.3 Hz, C-6'), 128.15 (C-2'), 127.08 (C-3'), 115.96 (d, *J* = 21.6 Hz, C-7').

*, signal could not be detected

**4-Chloro-(1,1'-biphenyl)-4-sulfonohydrazide (1j)**

**^1^H NMR in DMSO**


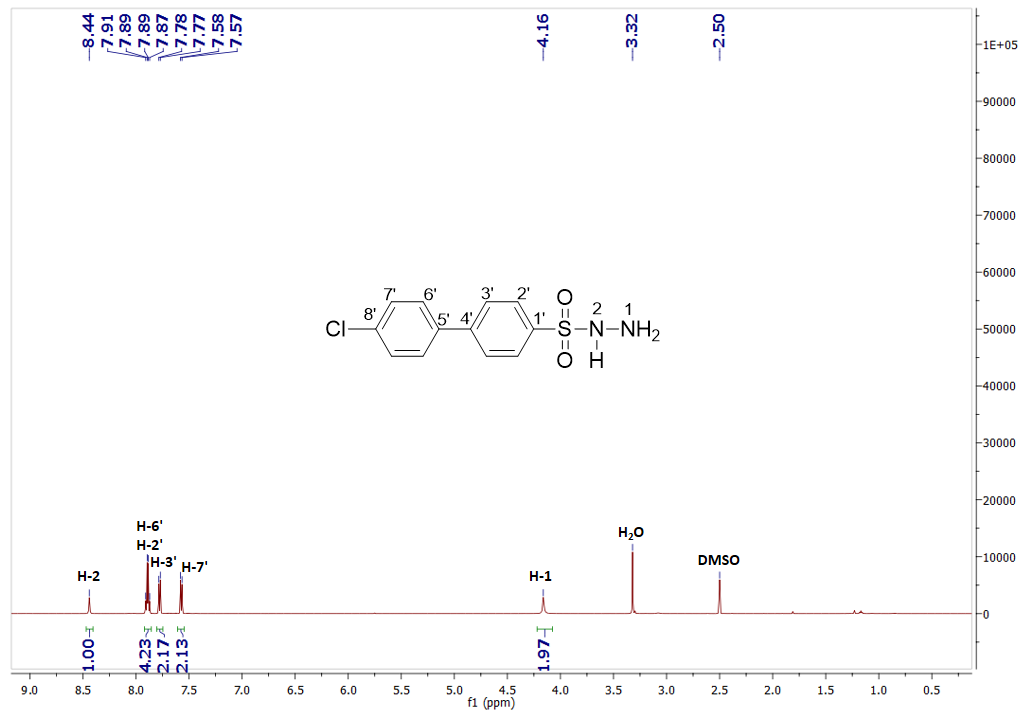


**^13^C NMR in DMSO**


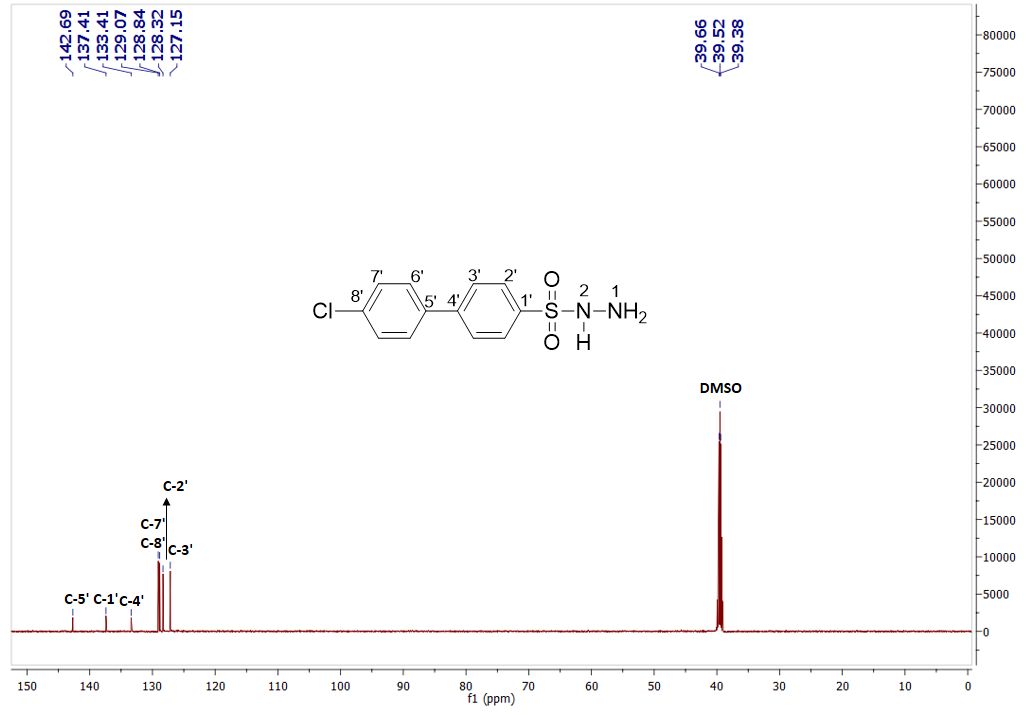


**IR Spectrum**

**
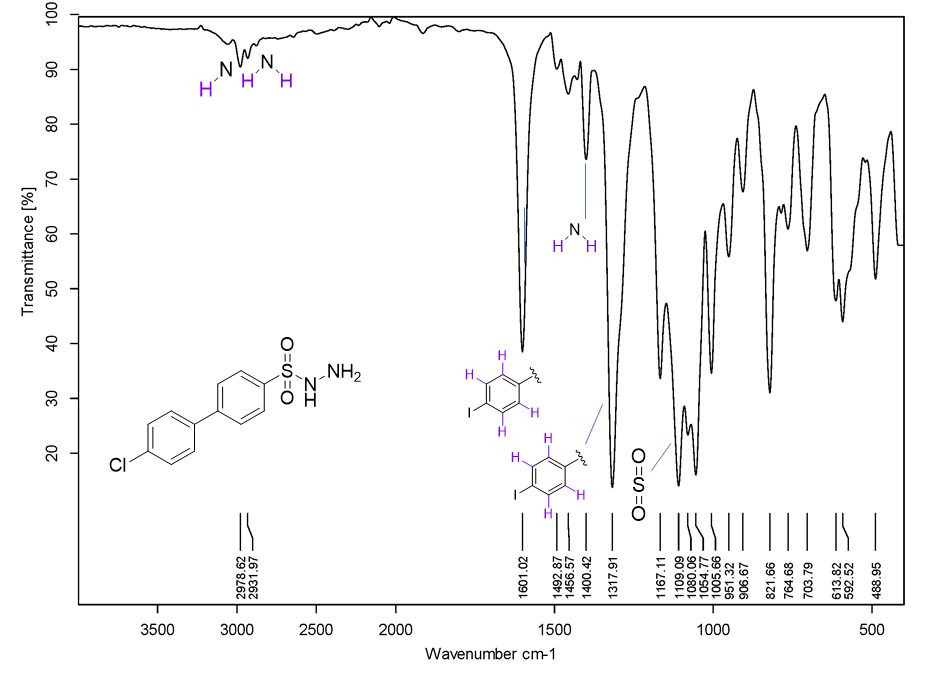
**

White solid, yield: 95%, mp: 150-153 °C, R*_f_* = 0.40 (EtOAc:*n*-Hex, 1:1, v/v), IR υ_max_ (cm^-1^): 2978 (N-H), 2931 (NH_2_), 1601 (Ar-H), 1492 (NH_2_), 1317 (Ar-H), 1109 (S=O).^1^H NMR (600 MHz, DMSO) δ 8.44 (s, 1H, H-2), 7.89 (q, *J* = 8.5 Hz, 4H, H-6'/2'), 7.78 (d, *J* = 8.4 Hz, 2H, H-3'), 7.57 (d, *J* = 8.4 Hz, 2H, H-7'), 4.16 (s, 2H, H-1). ^13^C NMR (151 MHz, DMSO) δ 142.69 (C-5'), 137.41 (C-1'), 133.41 (C-4'), 129.07 (C-7'), 128.84 (C-8'), 128.32 (C-2'), 127.15 (C-3').

**4-Methoxy-(1,1'-biphenyl)-4-sulfonohydrazide (1k)**

**^1^H NMR in DMSO**


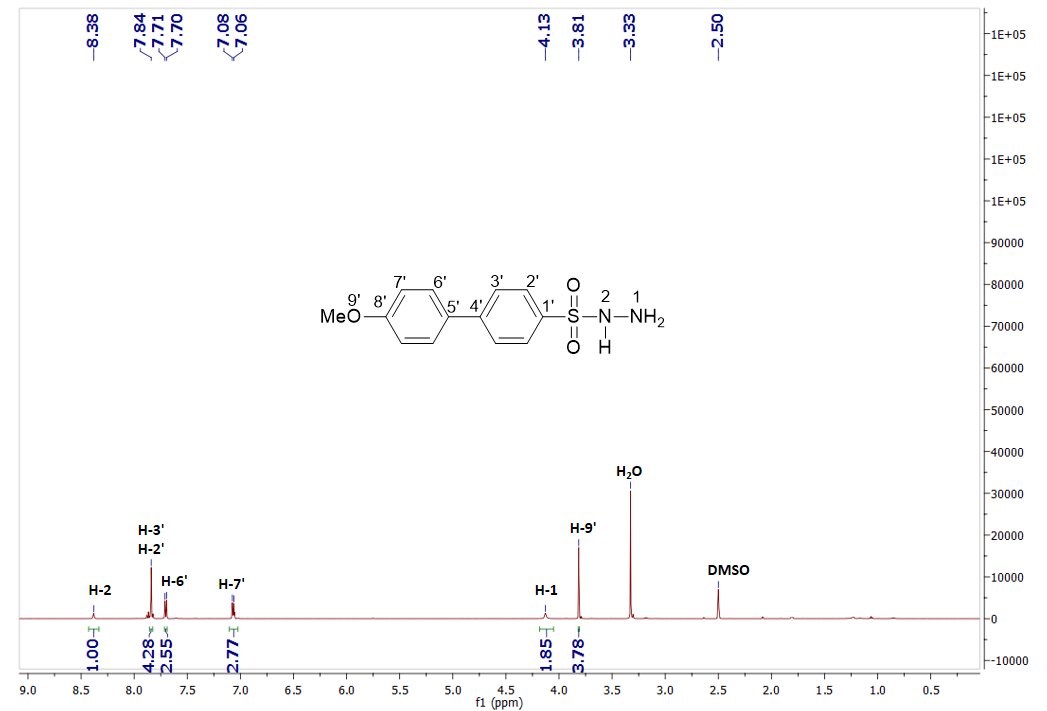


**^13^C NMR in DMSO**


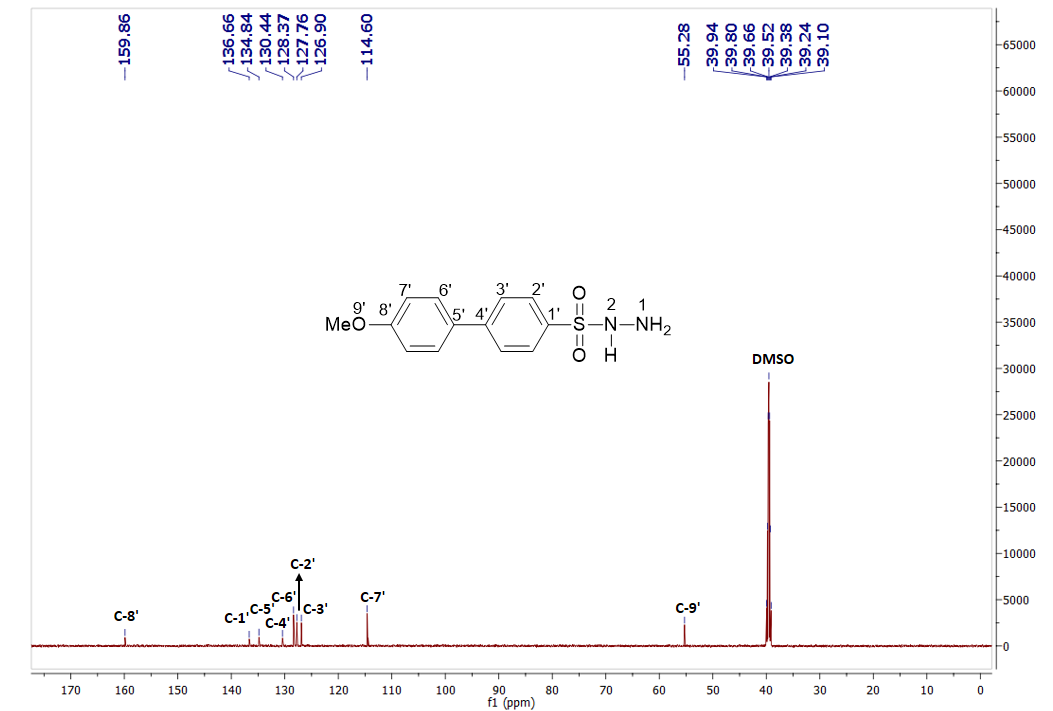


**IR Spectrum**

**
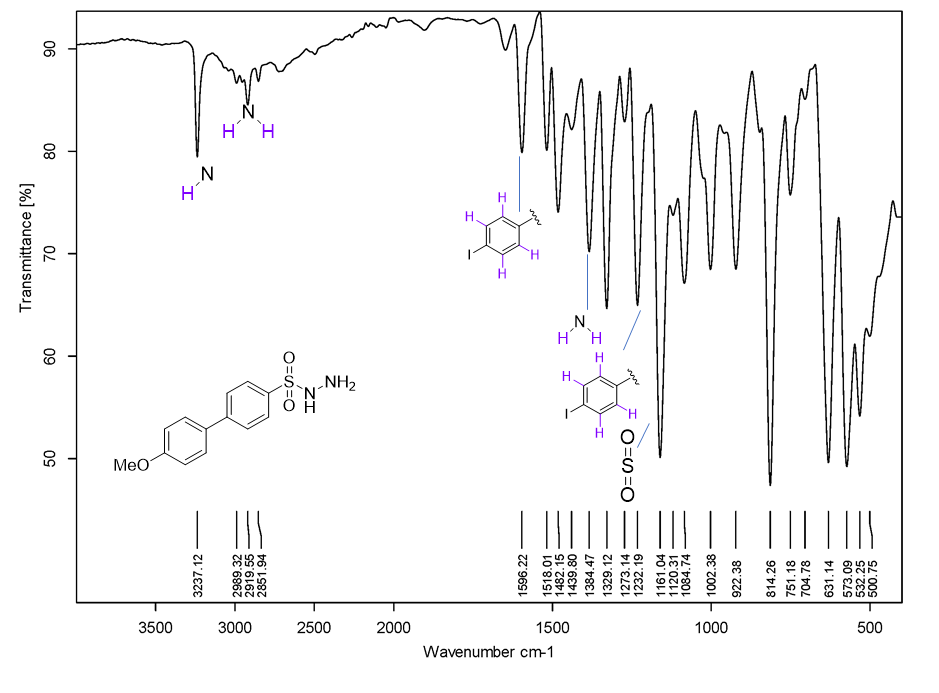
**

White solid, yield: 95%, mp: 173-175 °C, R*_f_* = 0.30 (EtOAc:*n*-Hex, 1:1, v/v), IR υ_max_ (cm^-1^): 3237 (N-H), 2989 (NH_2_), 1596 (Ar-H), 1439 (NH_2_), 1329 (Ar-H), 1161 (S=O).^1^H NMR (600 MHz, DMSO) δ 8.38 (s, 1H, H-2), 7.84 (s, 4H, H-3'/2'), 7.70 (d, *J* = 8.8 Hz, 2H, H-6'), 7.07 (d, *J* = 8.8 Hz, 2H, H-7'), 4.13 (s, 2H, H-1), 3.81 (s, 3H, H-9'). ^13^C NMR (151 MHz, DMSO) δ 159.86 (C-8'), 136.66 (C-1'), 134.84 (C-5'), 130.44 (C-4'), 128.37 (C-6'), 127.76 (C-2'), 126.90 (C-3'), 114.60 (C-7'), 55.28 (C-9').

**2,3-Dihydrobenzo[*b*][1,4]dioxine-5-sulfonohydrazide (1l)**

**^1^H NMR in DMSO**


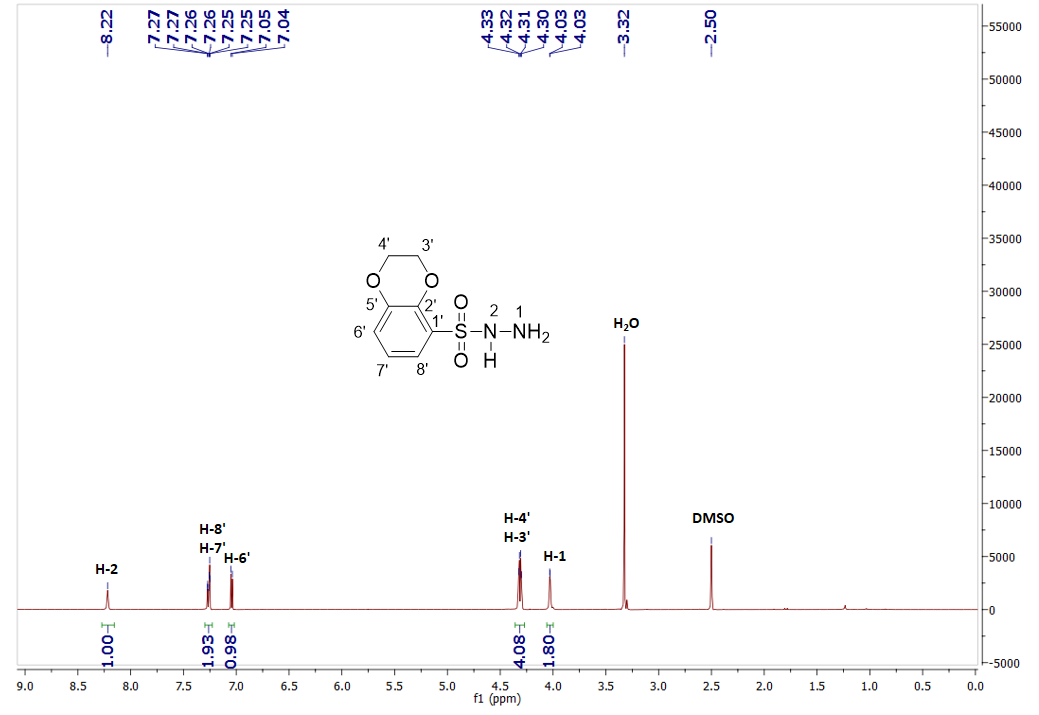


**^13^C NMR in DMSO**


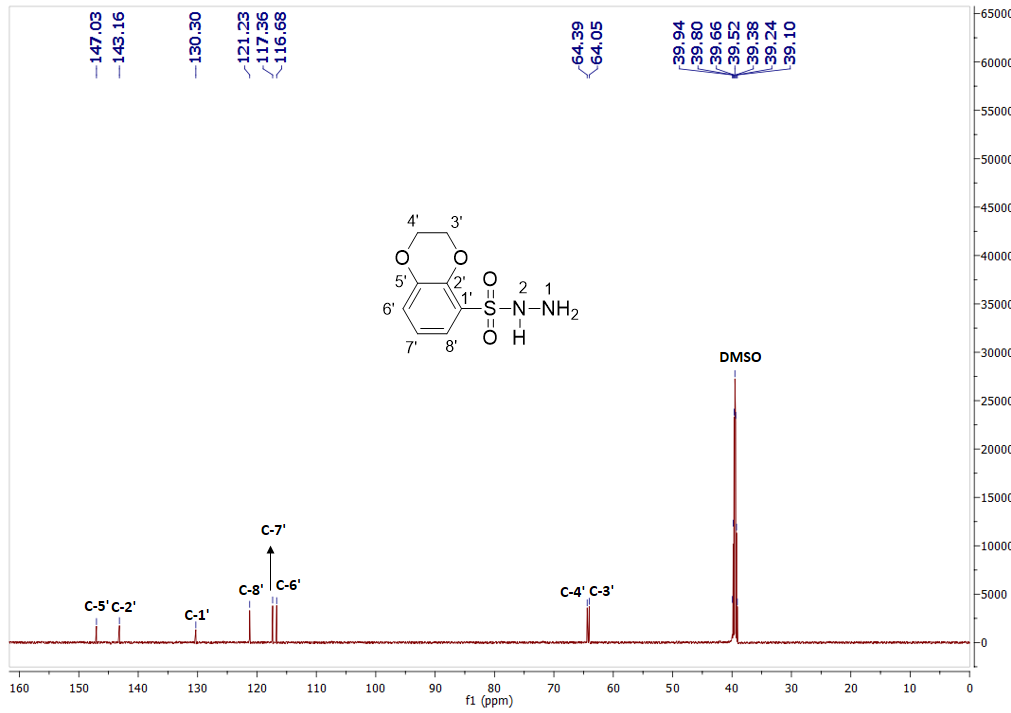


**IR Spectrum**

**
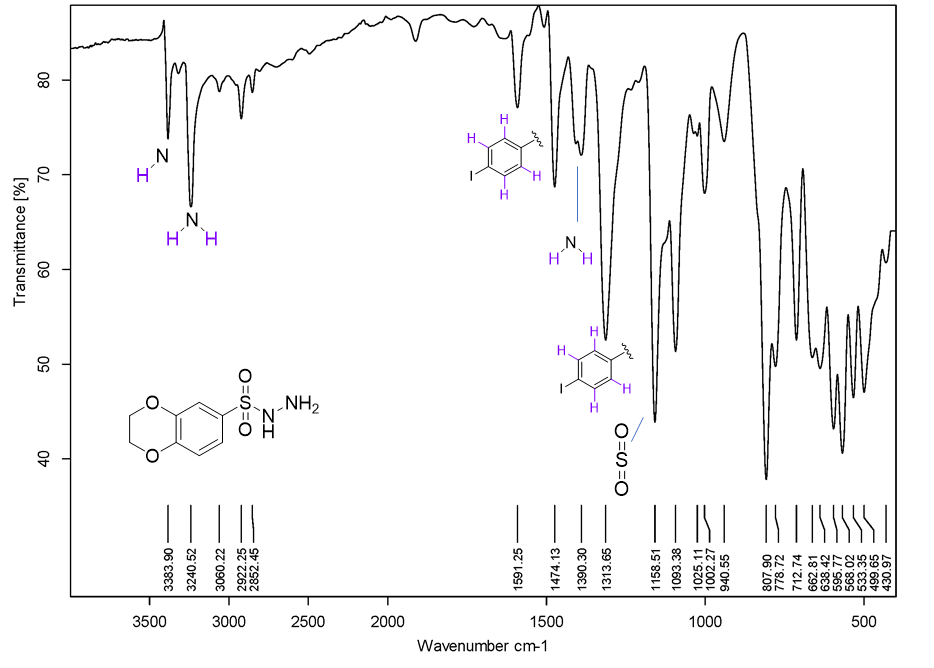
**

White solid, yield: 80%, mp: 101-103 °C, R*_f_* = 0.20 (EtOAc:*n*-Hex, 1:1, v/v), IR υ_max_ (cm^-1^): 3383 (N-H), 3240 (NH_2_), 1591 (Ar-H), 1474 (NH_2_), 1313 (Ar-H), 1158 (S=O). ^1^H NMR (600 MHz, DMSO) δ 8.22 (s, 1H, H-2), 7.28 – 7.25 (m, 2H, H-8'/7'), 7.04 (d, *J* = 8.3 Hz, 1H, H-6'), 4.31 (dd, *J* = 12.4, 5.1 Hz, 4H, H-4'/3'), 4.03 (s, 2H, H-1). ^13^C NMR (151 MHz, DMSO) δ 147.03 (C-5'), 143.16 (C-2'), 130.30 (C-1'), 121.23 (C-8'), 117.36 (C-7'), 116.68 (C-6'), 64.39 (C-4'), 64.05 (C-3').

***(E)*-4-Iodo-*N'*-[(5-nitrofuran-2-yl)methylene]benzenesulfonohydrazide** (**2a**)

**^1^H NMR in DMSO**

**
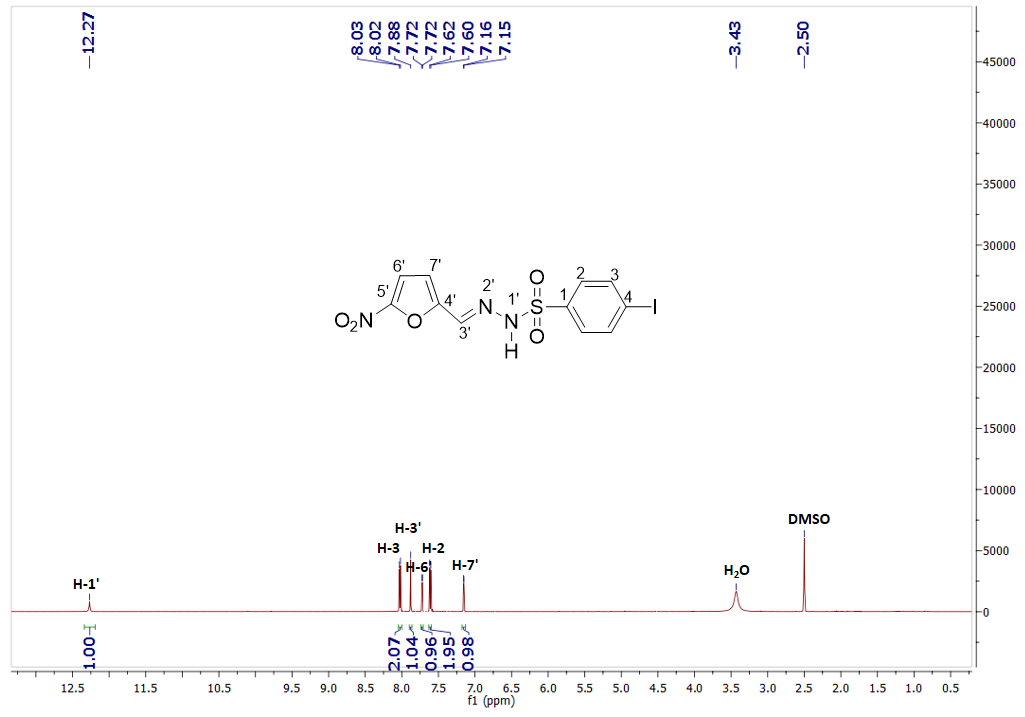
**

**^13^C NMR in DMSO**

**
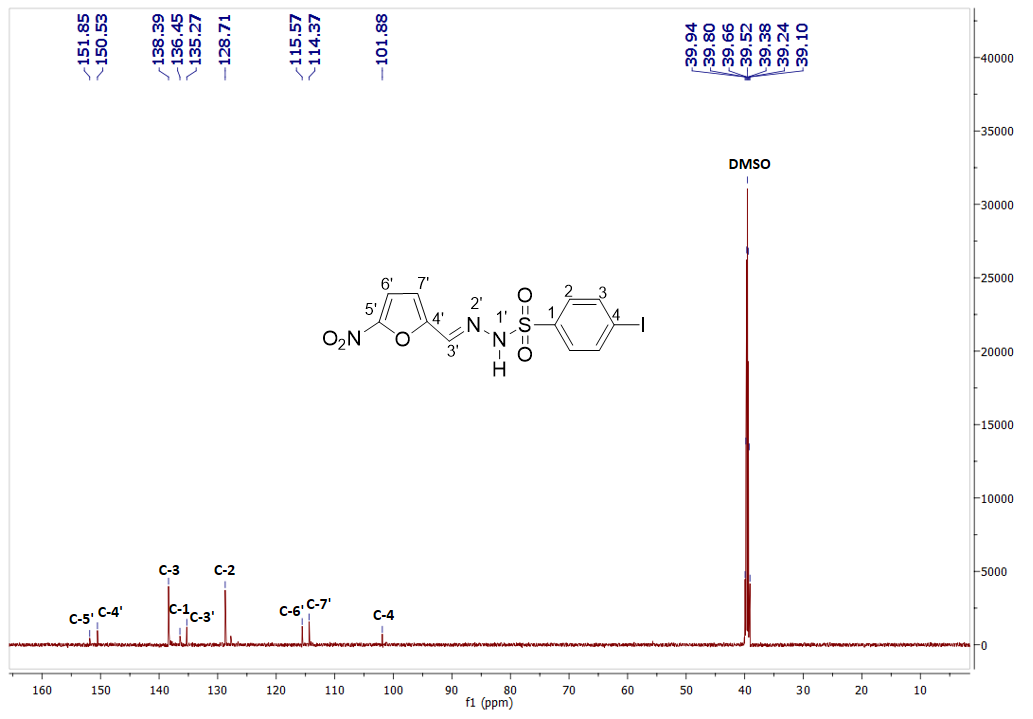
**

**IR Spectrum**


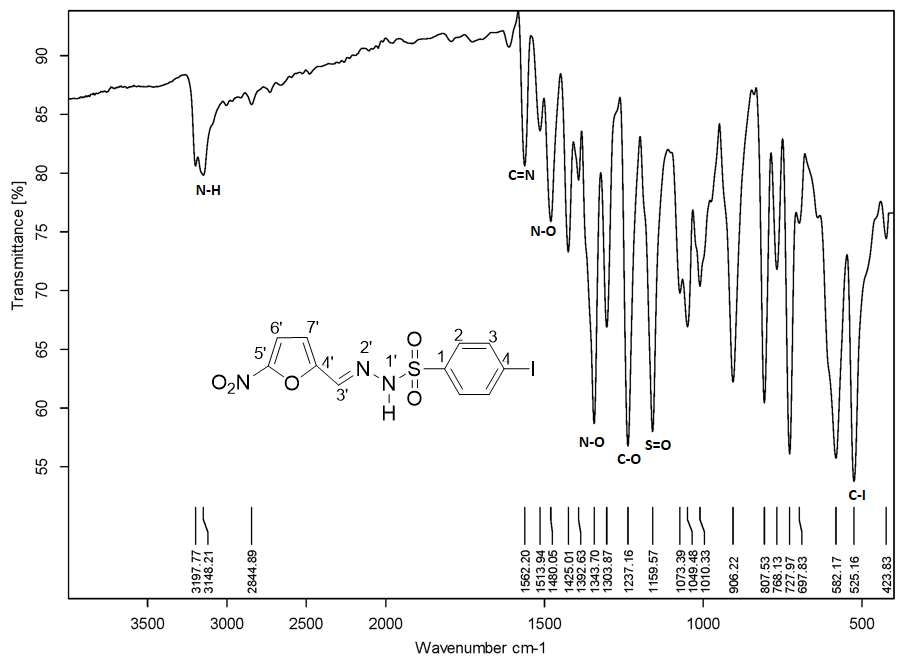


**HRMS**

**
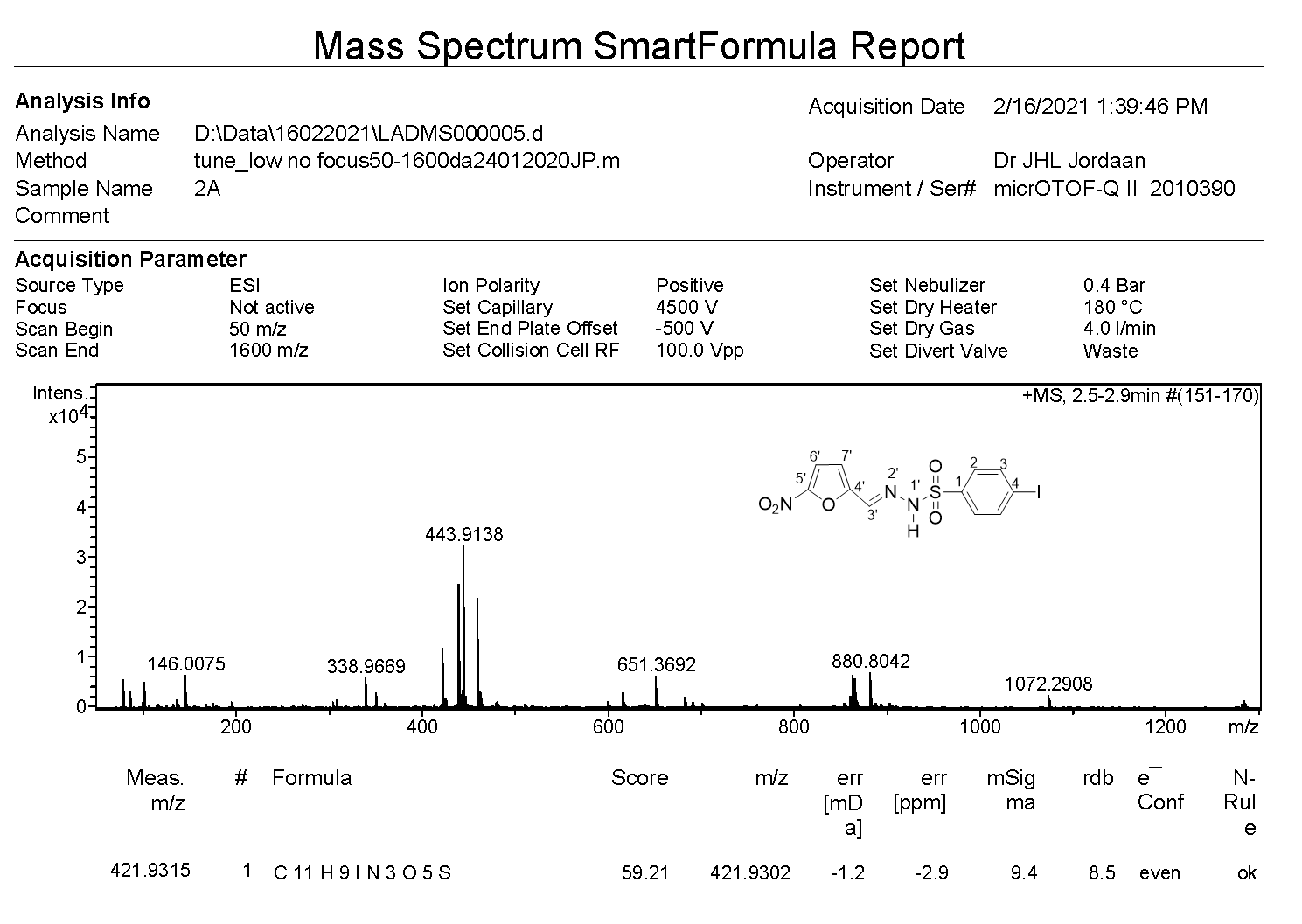
**

Light brown solid, yield: 95%, mp: 161-164 °C (EtOAc), R*_f_* = 0.40 (EtOAc:*n*-Hex 1:1, v/v ), IR u_max_ (cm^-1^): 3148 (N-H), 1562 (C=N), 1480 (N-O), 1480 (S=O), 1343 (N-O), 1237 (C-O), 1159 (S=O), 525 (C-Cl).^1^H NMR (600 MHz, DMSO) δ 12.27 (s, 1H, H-1'), 8.03 (d, *J* = 8.4 Hz, 2H, H-3), 7.88 (s, 1H, H-3'), 7.72 (d, *J* = 3.9 Hz, 1H, H-6'), 7.61 (d, *J* = 8.4 Hz, 1H, H-2), 7.15 (d, *J* = 3.9 Hz, 1H, H-7'). ^13^C NMR (151 MHz, DMSO) δ 151.85 (C-5'), 150.53 (C-4'), 138.39 (C-3), 136.45 (C-1), 135.27 (C-3'), 128.71 (C-2), 115.57 (C-7'), 114.37 (C-6'), 101.88 (C-4). HRMS-ESI (*pos*) *m/z* 421.9302 [M + H] ^+^ (Calcd for C_11_H_9_IN_3_O_5_S^+^, 421.9308).

**(*E*)-4-Nitro-*N'*-[(5-nitrofuran-2-yl)methylene]benzenesulfonohydrazide (2b)**

**^1^H NMR in DMSO**

**
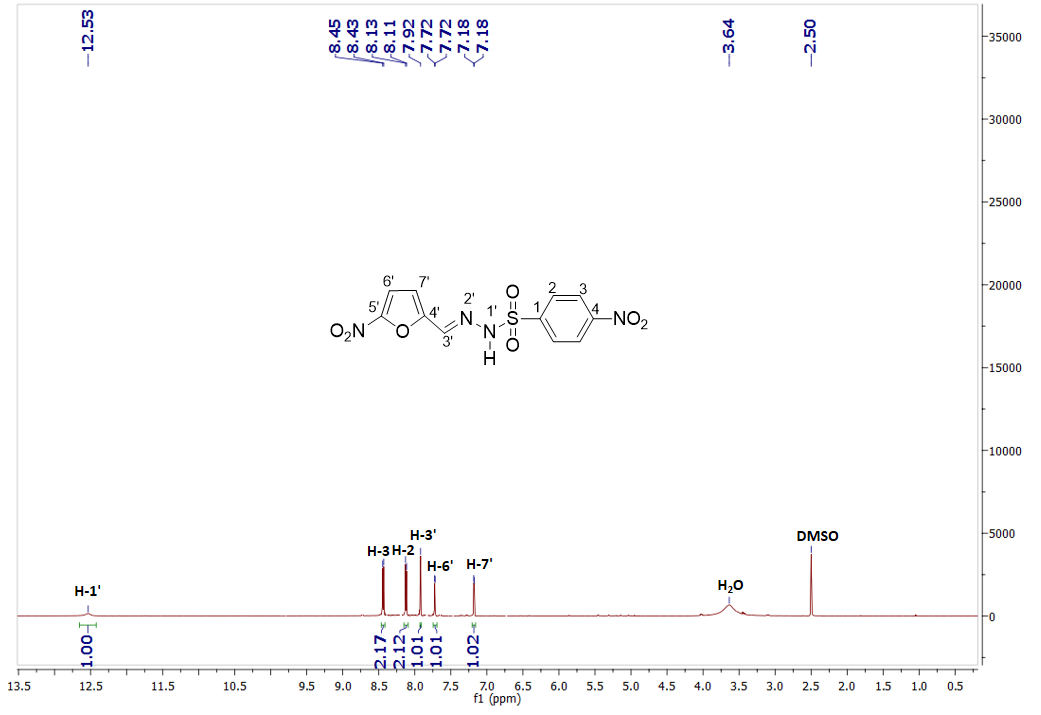
**

**^13^C NMR in DMSO**

**
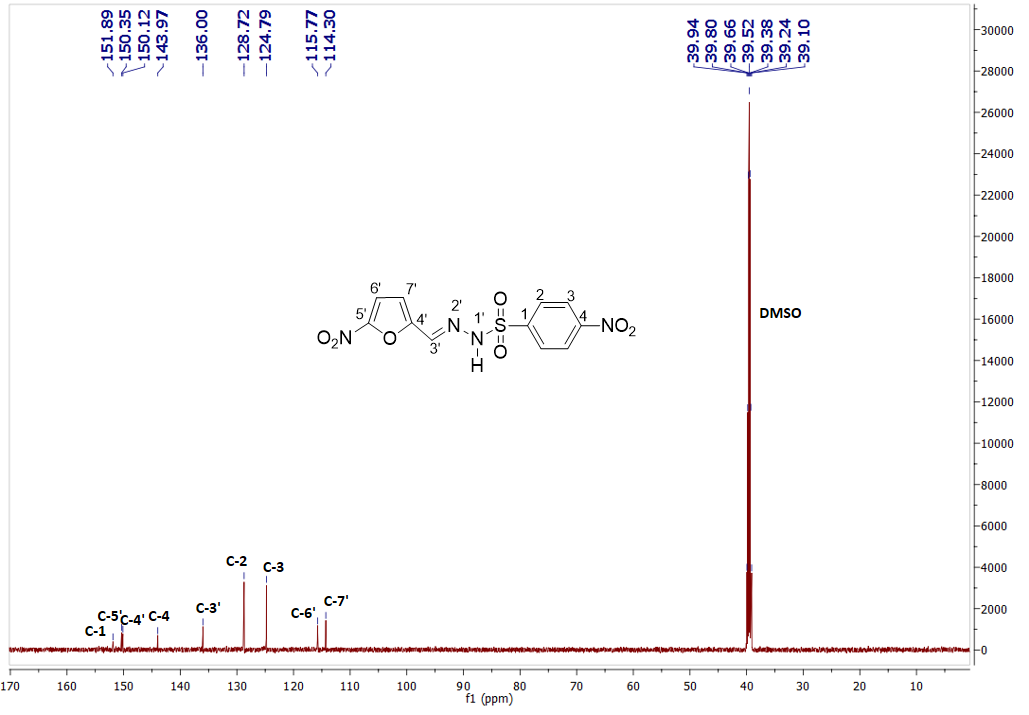
**

**IR Spectrum**

**
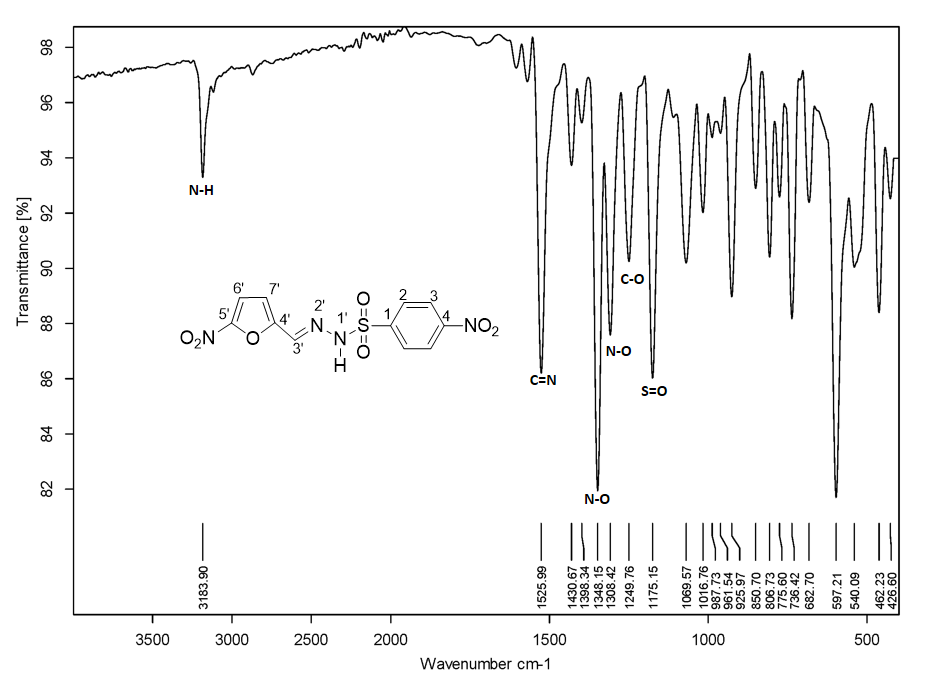
**

**HRMS**

**
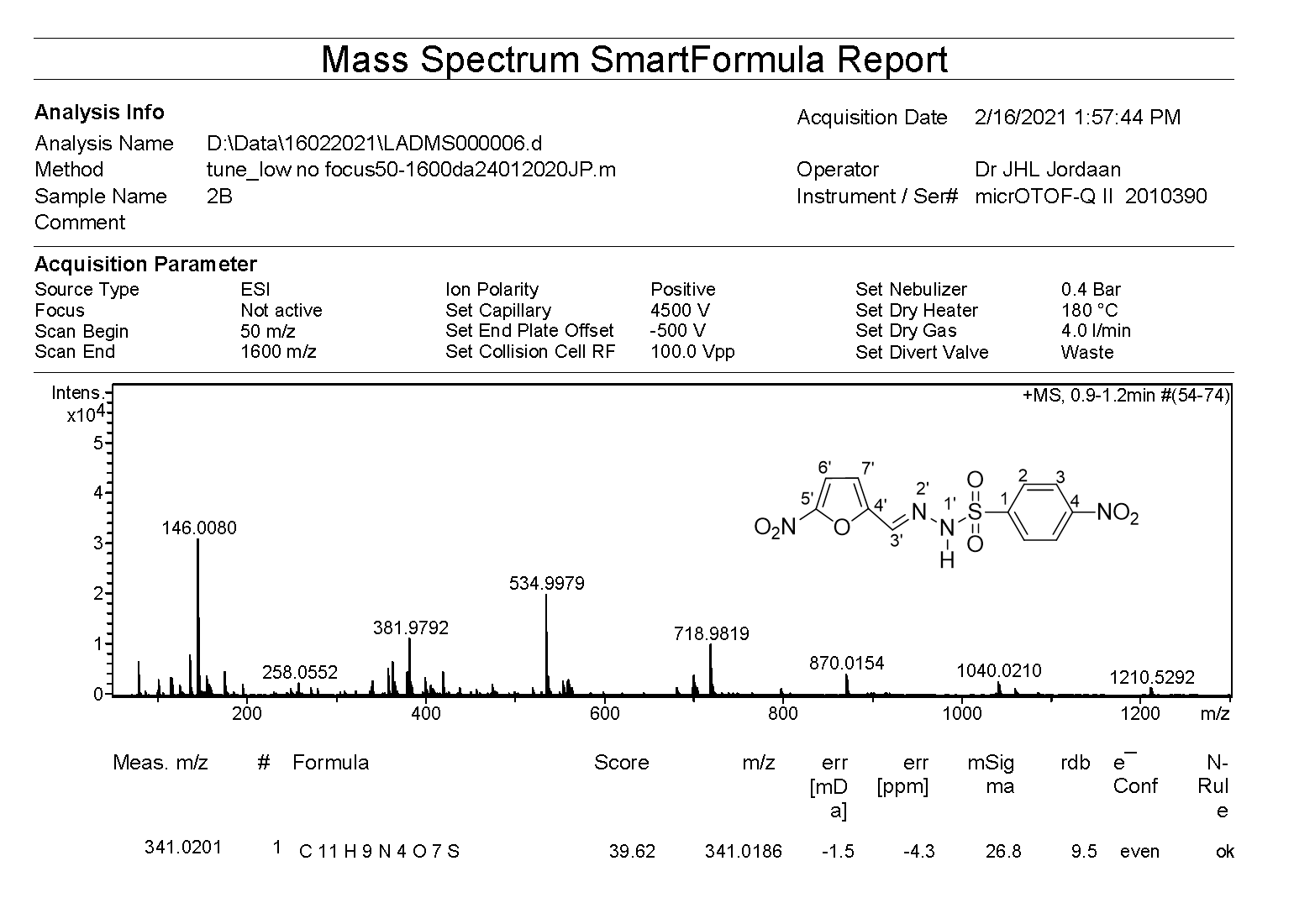
**

Yellow solid, yield: 90%, mp: 179-182 °C (EtOAc), R*_f_* = 0.10 (EtOAc:*n*-Hex 1:1, v/v ), IR υ_max_ (cm^-1^): 3183 (N-H), 1525 (C=N), 1430 (S=O), 1348 (N-O), 1308 (N-O), 1249 (C-O), 1175 (S=O).^1^H NMR (600 MHz, DMSO) δ 12.53 (s, 1H, H-1'), 8.44 (d, *J* = 8.8 Hz, 2H, H-3), 8.12 (d, *J* = 8.8 Hz, 2H, H-2), 7.92 (s, 1H, H-3'), 7.72 (d, *J* = 3.9 Hz, 1H, H-6'), 7.18 (d, *J* = 3.9 Hz, 1H, H-7'). ^13^C NMR (151 MHz, DMSO) δ 151.89 (C-1), 150.35 (C-5'), 150.12 (C-4'), 143.97 (C-4), 136.00 (C-3'), 128.72 (C-2), 124.79 (C-3), 115.77 (C-6'), 114.30 (C-7'). HRMS-ESI (*pos*) *m/z* 341.0186 [M + H] ^+^ (calcd for C_11_H_9_N_4_O_7_S^+^, 341.0192).

**(*E*)-4-Methyl-*N'*-[(5-nitrofuran-2-yl)methylene]benzenesulfonohydrazide (2c)**

**^1^H NMR in DMSO**

**
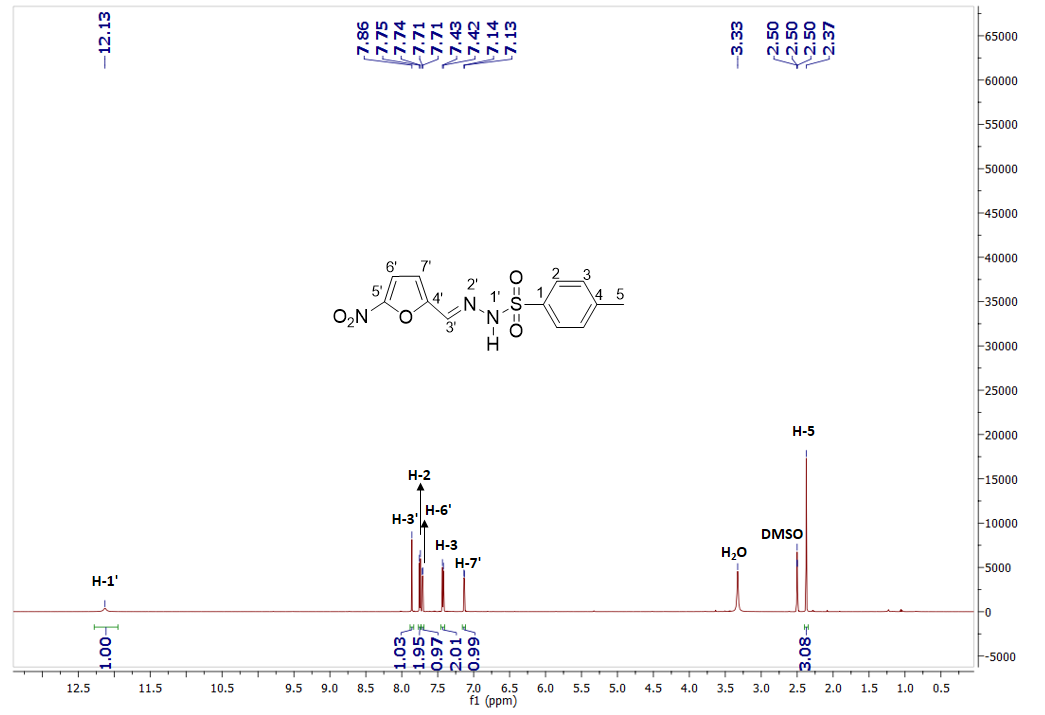
**

**^13^C NMR in DMSO**

**
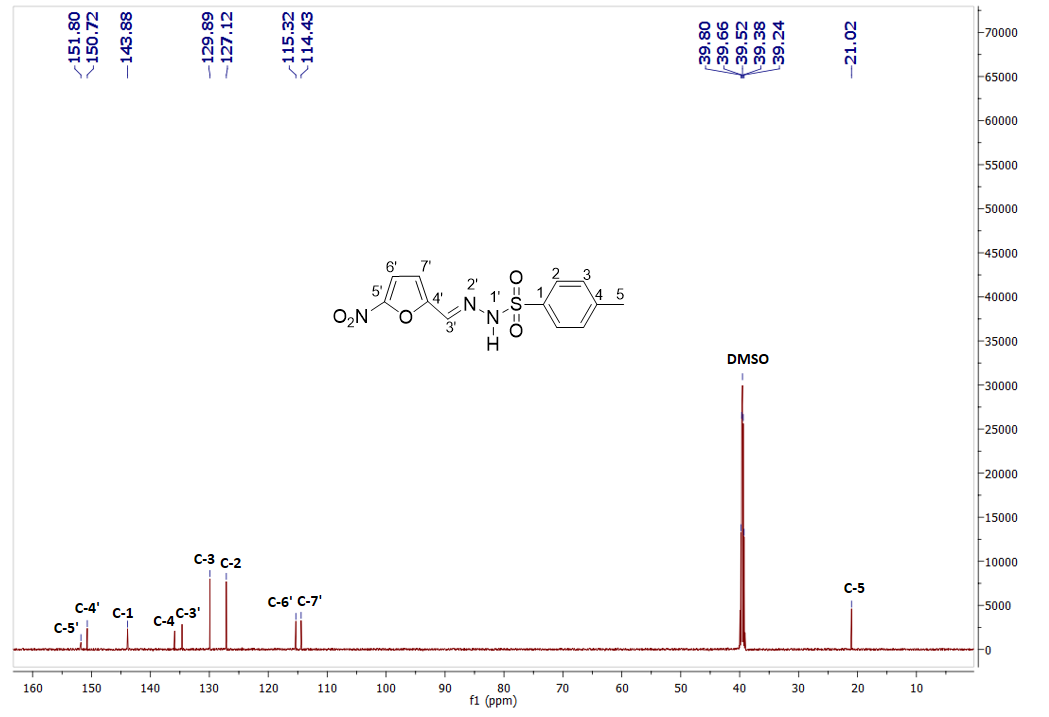
**

**IR Spectrum**

**
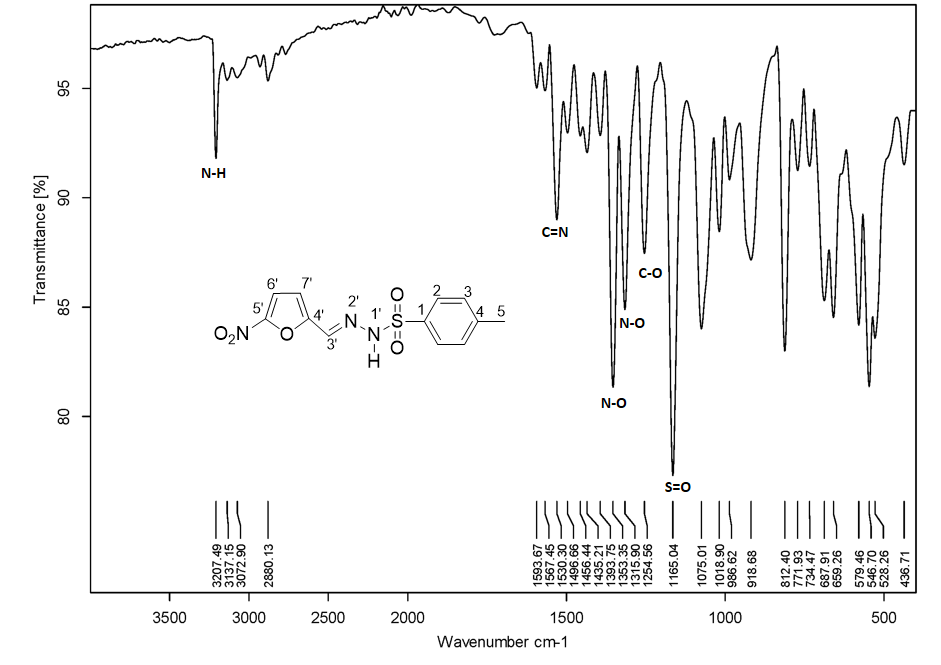
**

**HRMS**


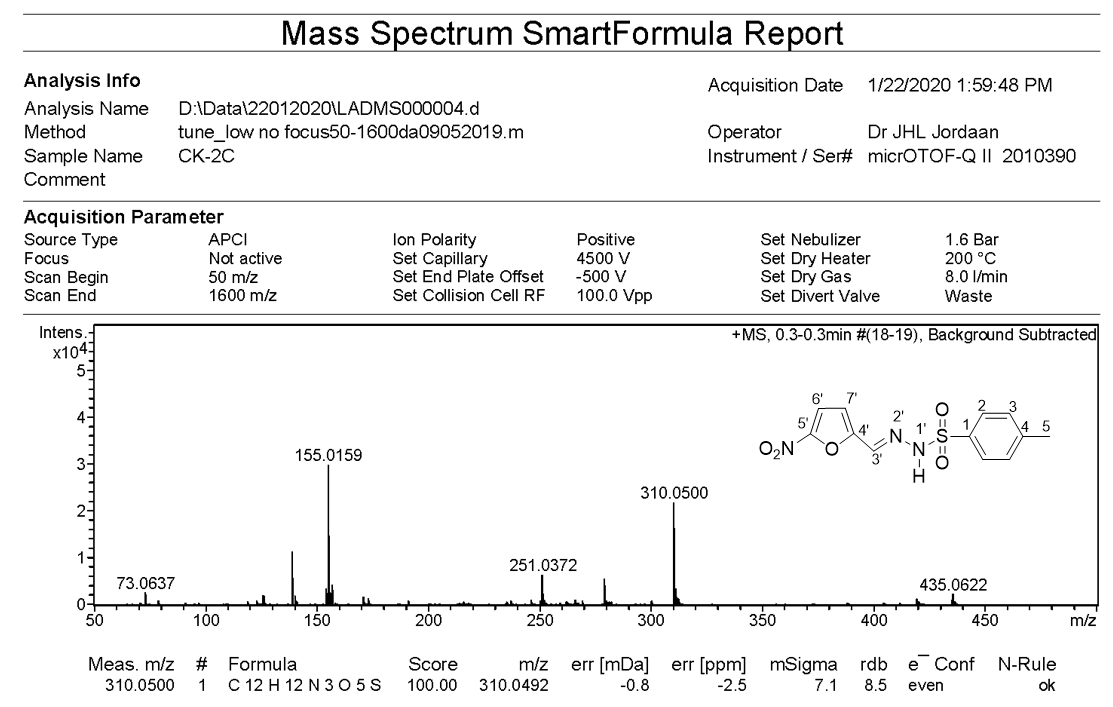


Light brown solid, yield: 92%, mp: 137-140 °C (EtOAc) (Lit. ^1^ 106-108°C), R*_f_* = 0.50 (EtOAc:*n*-Hex 1:1, v/v ), IR υ_max_ (cm^-1^): 3137 (N-H), 1567 (C=N), 1496 (S=O), 1392 (N-O), 1353 (N-O), 1254 (C-O), 1165 (S=O).^1^H NMR (600 MHz, DMSO) δ 12.13 (s, 1H, H-1'), 7.86 (s, 1H, H-3'), 7.75 (d, *J* = 8.3 Hz, 2H, H-2), 7.71 (d, *J* = 3.9 Hz, 1H, H-6'), 7.43 (d, *J* = 8.3 Hz, 2H, H-3), 7.13 (d, *J* = 3.9 Hz, 1H, H-7'), 2.37 (s, 3H, H-5). ^13^C NMR (151 MHz, DMSO) δ 151.80 (C-5'), 150.72 (C-4'), 143.88 (C-1), 135.89 (C-4), 134.64 (C-3'), 129.89 (C-3), 127.12 (C-2), 115.32 (C-6'), 114.43 (C-7'), 21.02 (C-5). HRMS-APCI (*pos*) *m/z* 310.0500 [M + H]^+^ (calcd for C_12_H_12_N_3_O_5_S^+^, 310.0498).

**(*E*)-4-Isopropyl-*N'*-[(5-nitrofuran-2-yl)methylene]benzenesulfonohydrazide** (**2d**)

**^1^H NMR in DMSO**

**
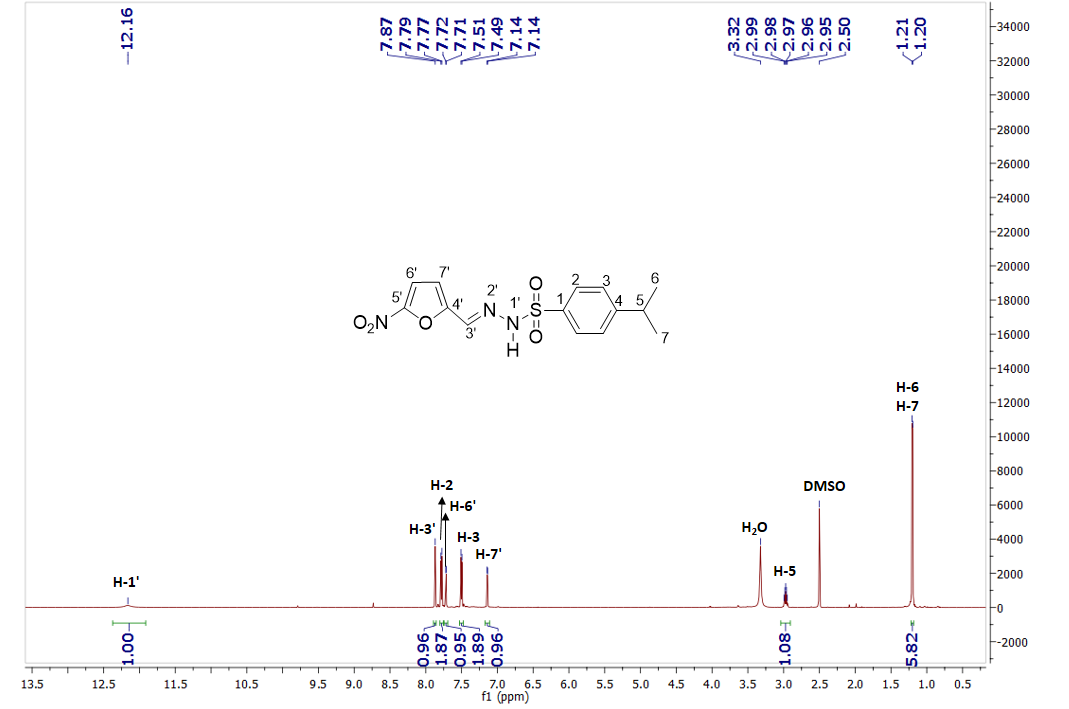
**

**^13^C NMR in DMSO**

**
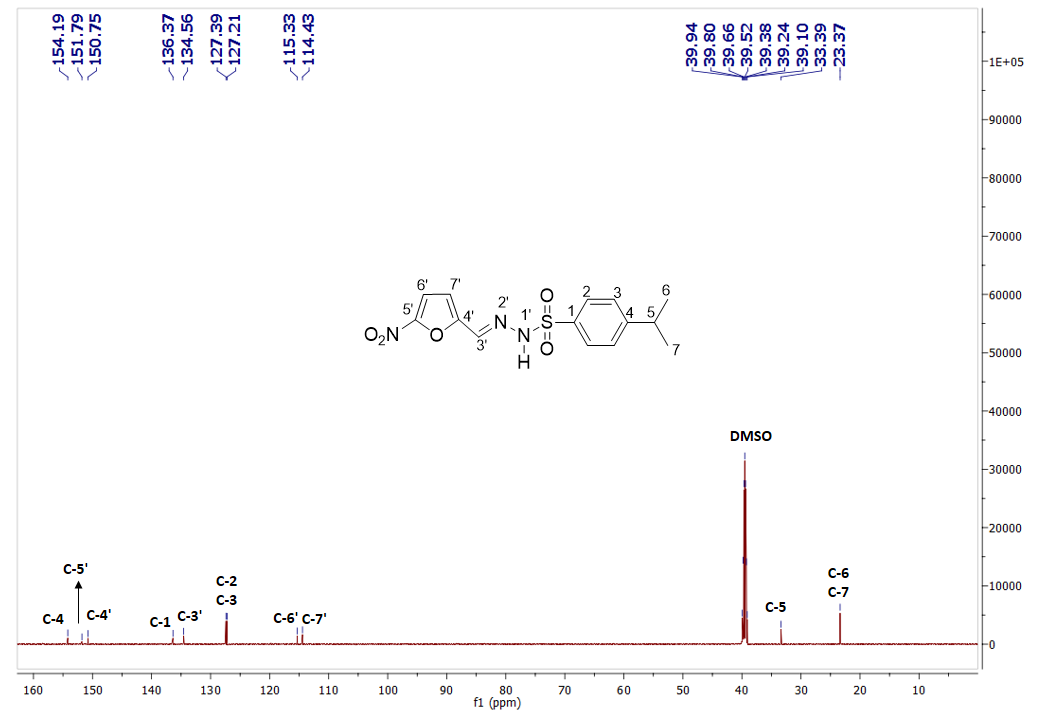
**

**IR Spectrum**

**
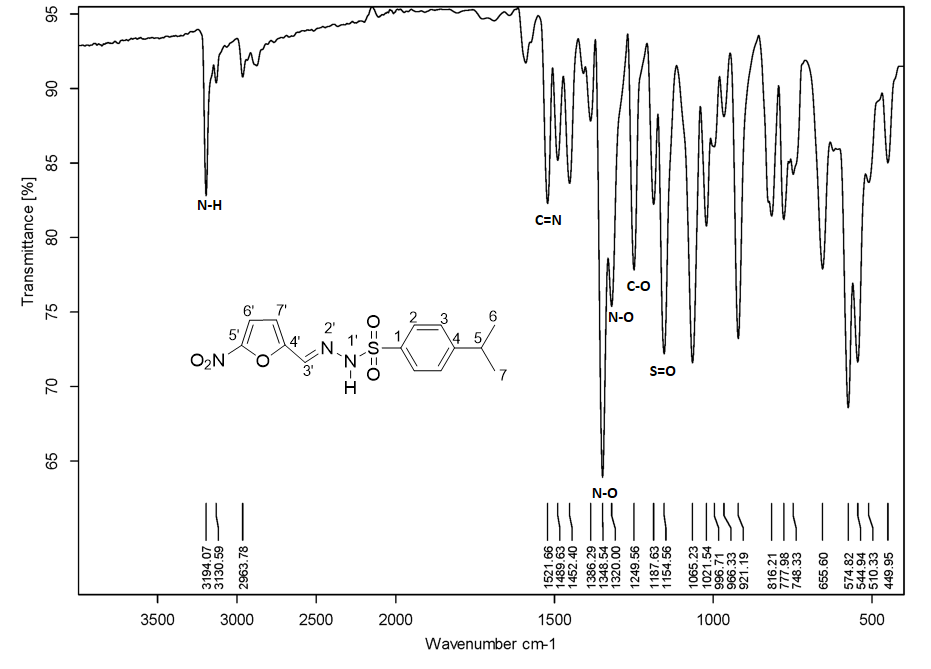
**

**HRMS**


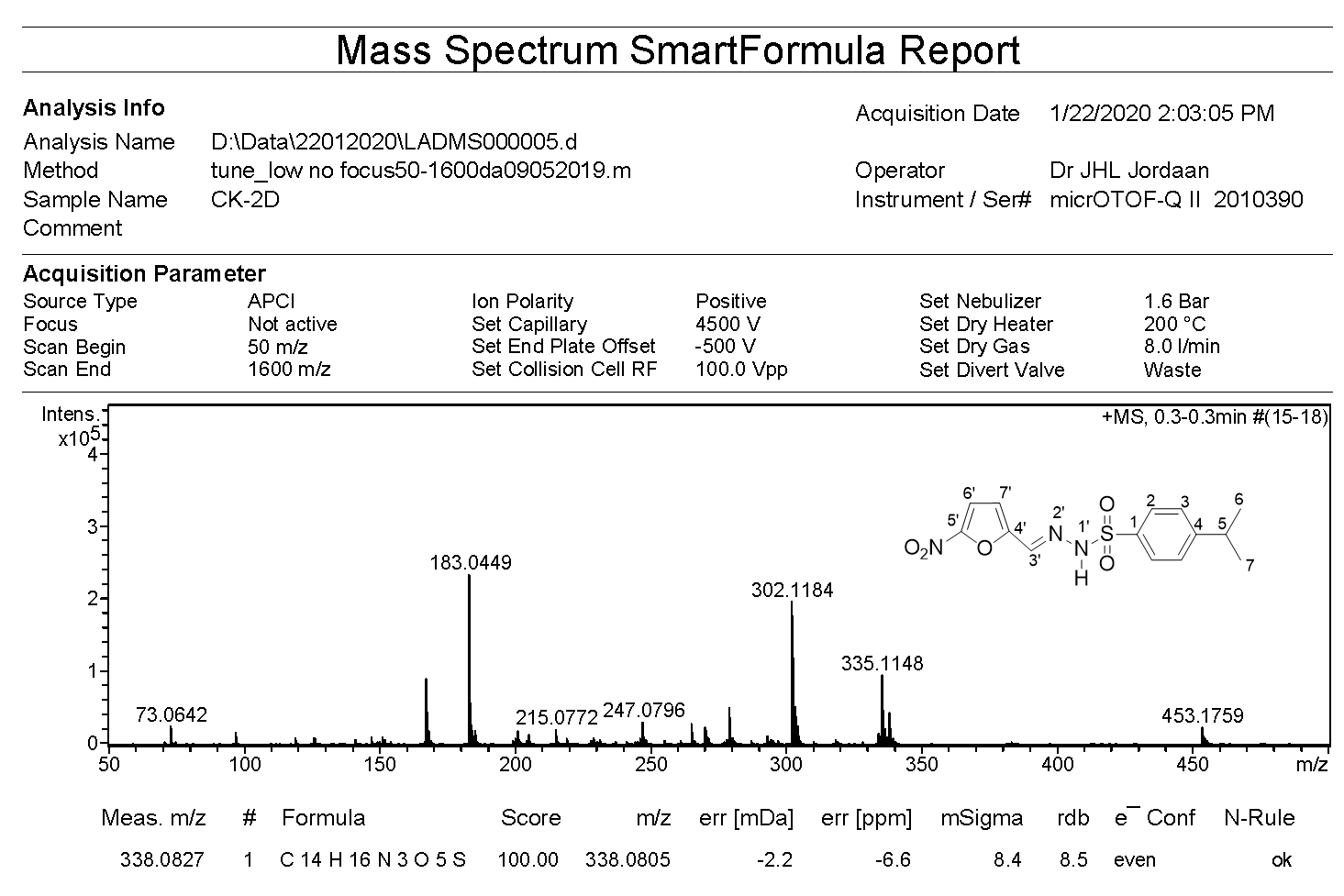


Light brown solid, yield: 94%, mp: 141-144 °C (EtOAc), R*_f_* = 0.50 (EtOAc:*n*-Hex 1:1, v/v ), IR υ_max_ (cm^-1^): 3130 (N-H), 1521 (C=N), 1452 (S=O), 1348 (N-O), 1320 (N-O), 1249 (C-O), 1154 (S=O).^1^H NMR (600 MHz, DMSO) δ 12.16 (s, 1H, H-1'), 7.87 (s, 1H, H-3'), 7.78 (d, *J* = 8.3 Hz, 2H, H-2), 7.72 (d, *J* = 3.9 Hz, 1H, H-6'), 7.50 (d, *J* = 8.3 Hz, 2H, H-3), 7.14 (d, *J* = 3.9 Hz, 1H, H-7'), 2.97 (dt, *J* = 13.8, 6.9 Hz, 1H, H-5), 1.21 (s, 3H, H-6), 1.20 (s, 3H, H-7). ^13^C NMR (151 MHz, DMSO) δ 154.19 (C-4), 151.79 (C-5'), 150.75 (C-4'), 136.37 (C-1), 134.56 (C-3'), 127.39 (C-2), 127.21 (C-3), 115.33 (C-6'), 114.43 (C-7'), 33.39 (C-5), 23.37 (C-6/7). HRMS-APCI (*pos*) *m/z* 338.0827 [M + H] ^+^ (Calcd for C_14_H_16_N_3_O_5_S^+^, 338.0811).

**(*E*)-2,4,6-Trimethyl-*N*'-[(5-nitrofuran-2-yl)methylene]benzenesulfonohydrazide (2e)**

**^1^H NMR in DMSO**

**
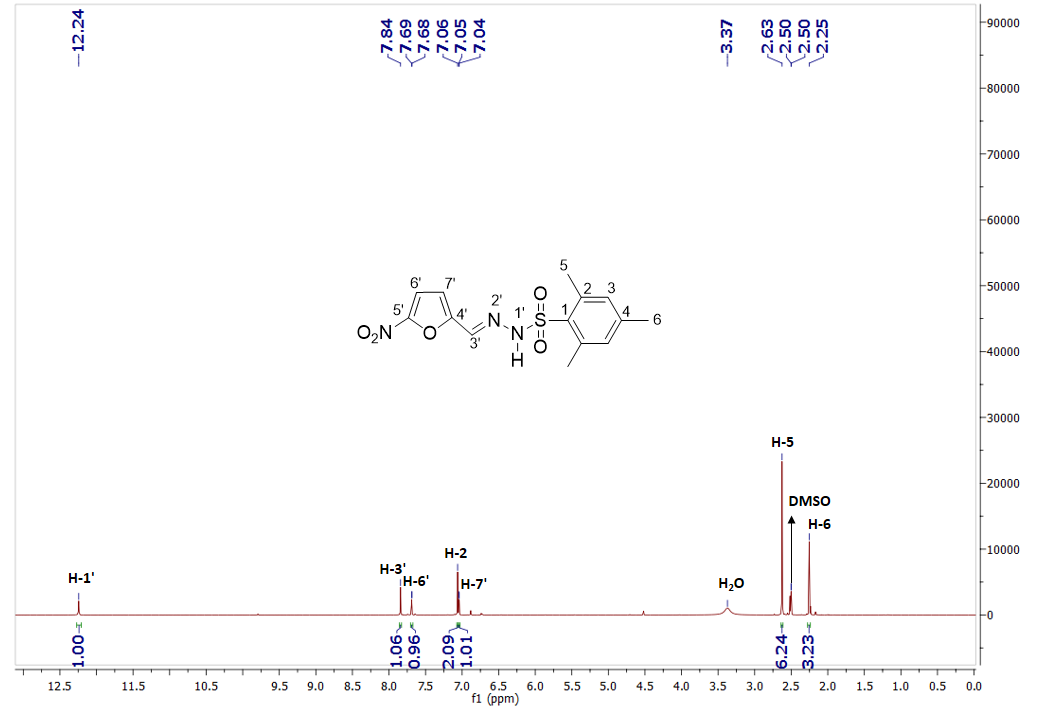
**

**^13^C NMR in DMSO**

**
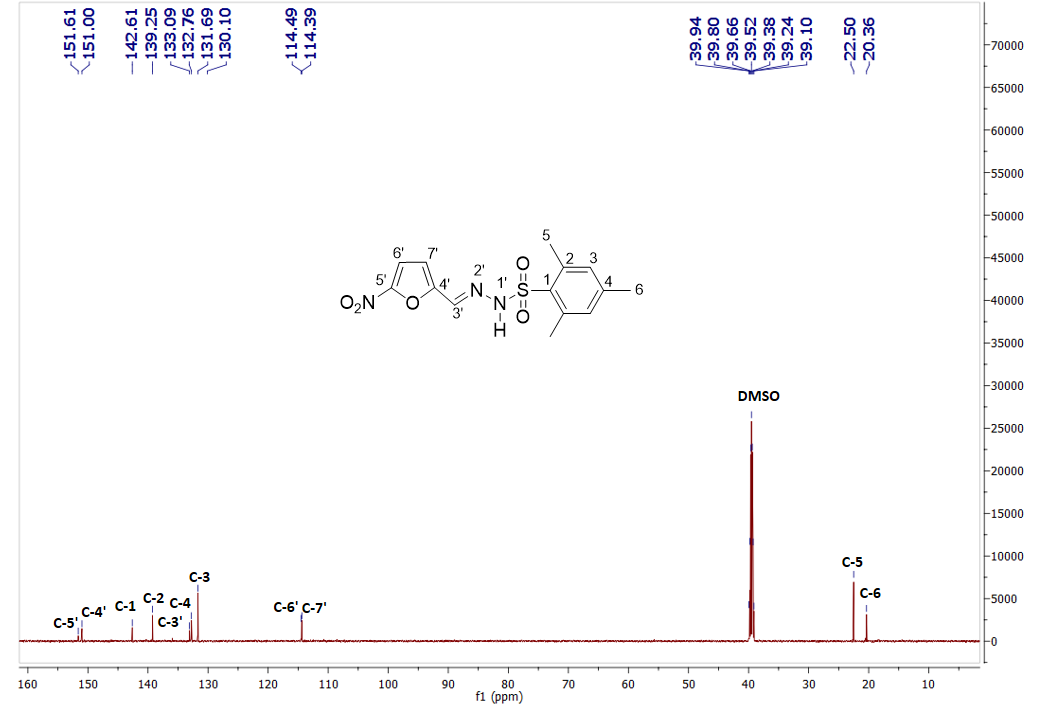
**

**IR Spectrum**

**
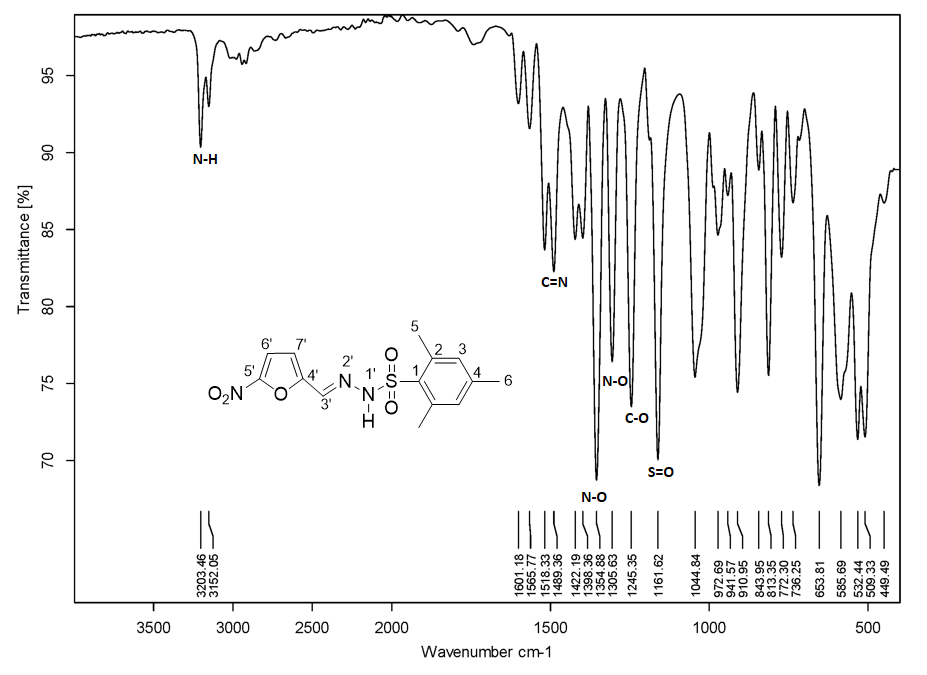
**

**HRMS**


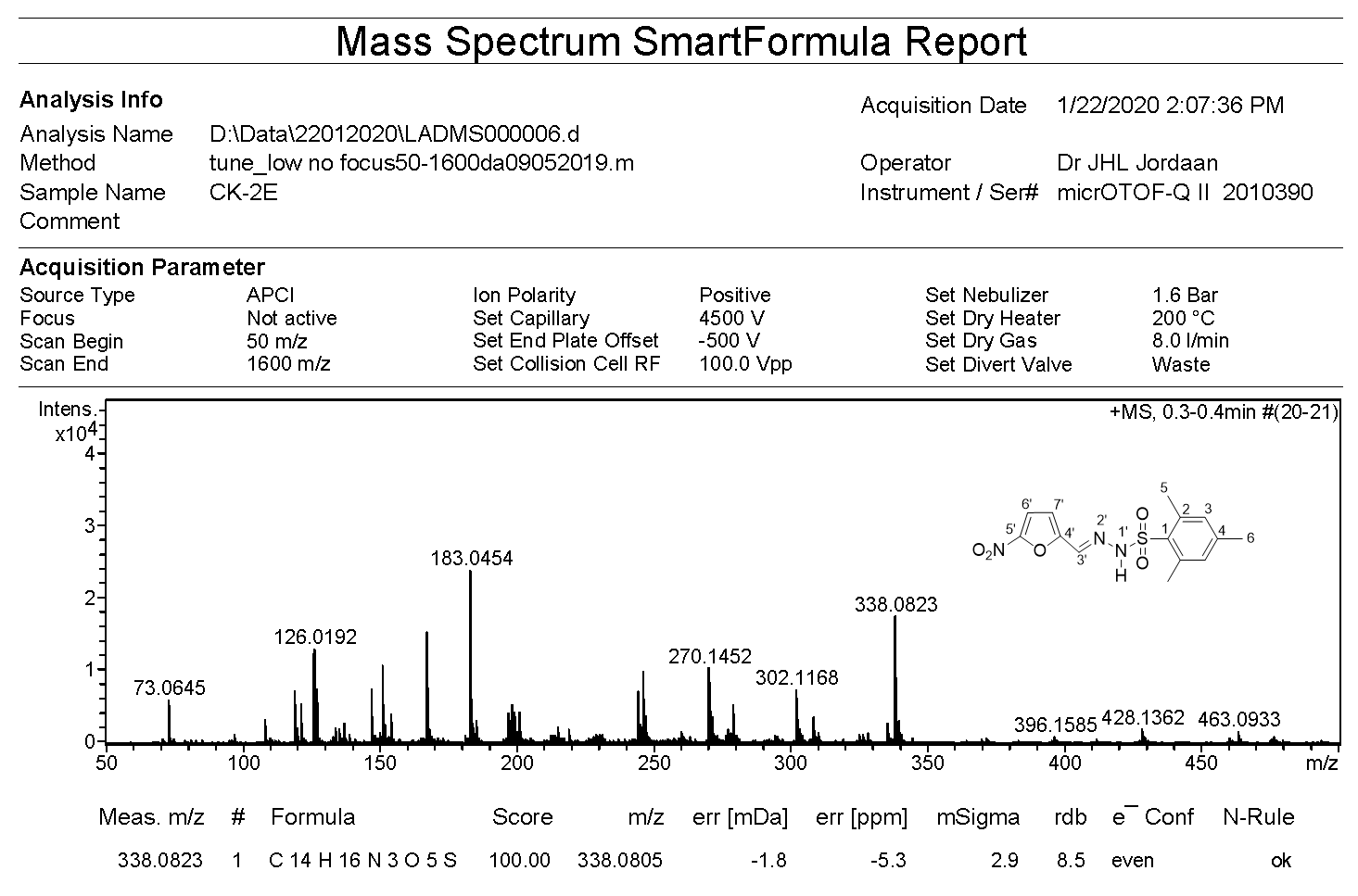


Light yellow solid, yield: 95%, mp: 173-176 °C (EtOAc), R*_f_* = 0.52 (EtOAc:*n*-Hex 1:1, v/v ) IR υ_max_ (cm^-1^): 3203 (N-H), 1518 (C=N), 1489 (S=O), 1398 (N-O), 1305 (N-O), 1245 (C-O), 1161 (S=O).^1^H NMR (600 MHz, DMSO) δ 12.24 (s, 1H, H-1'), 7.84 (s, 1H, H-3'), 7.69 (d, *J* = 3.9 Hz, 1H, H-6'), 7.06 (s, 2H, H-2), 7.04 (d, *J* = 3.9 Hz, 1H, H-7'), 2.63 (s, 6H, H-5), 2.25 (s, 3H, H-6). ^13^C NMR (151 MHz, DMSO) δ 151.61 (C-5'), 151.00 (C-4'), 142.61 (C-1), 139.25 (C-2), 133.09 (C-3'), 132.76 (C-4), 131.69 (C-3), 114.49 (C-6'), 114.39 (C-7'), 22.50 (C-5), 20.36 (C-6). HRMS-APCI (*pos*) *m/z* 338.0823 [M + H] ^+^ (Calcd for C_14_H_16_N_3_O_5_S^+^, 338.0811).

**(*E*)-4-(*tert*-**B**utyl)-*N'*-[(5-nitrofuran-2-yl)methylene]benzenesulfonohydrazide (2f)**

**^1^H NMR in DMSO**

**
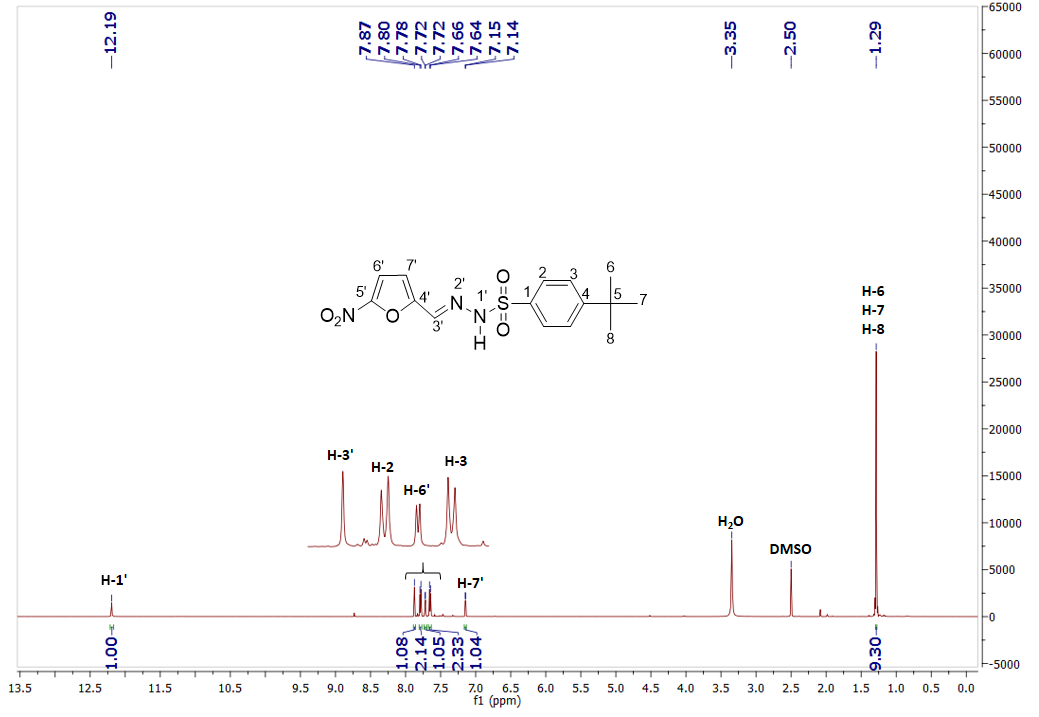
**

**^13^C NMR in DMSO**

**
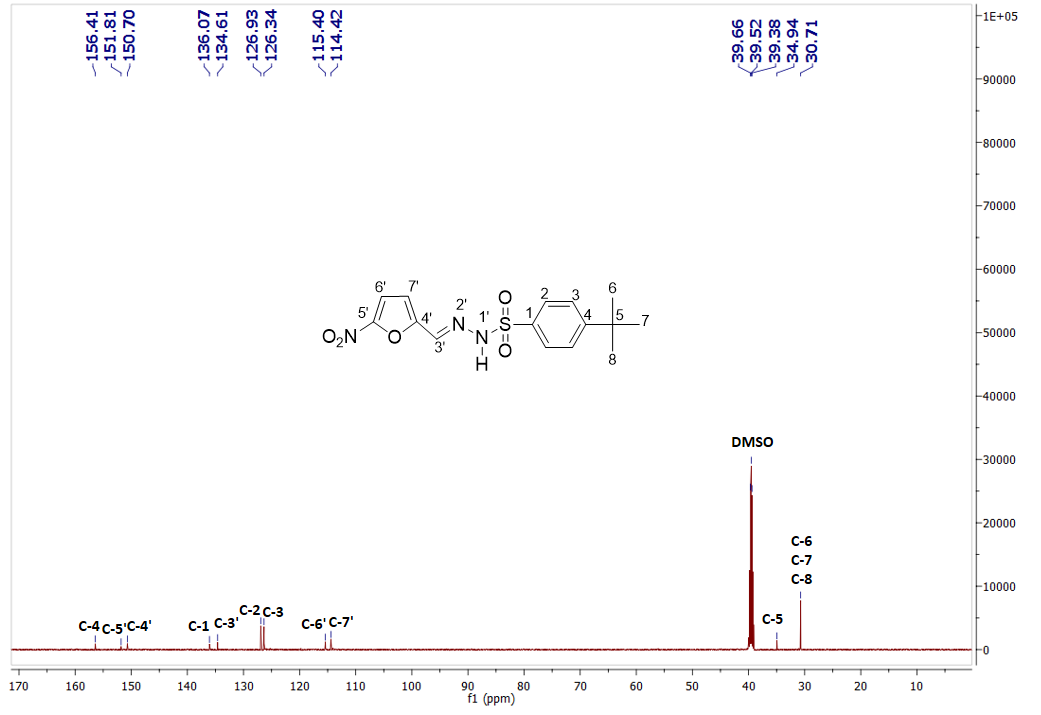
**

**IR Spectrum**

**
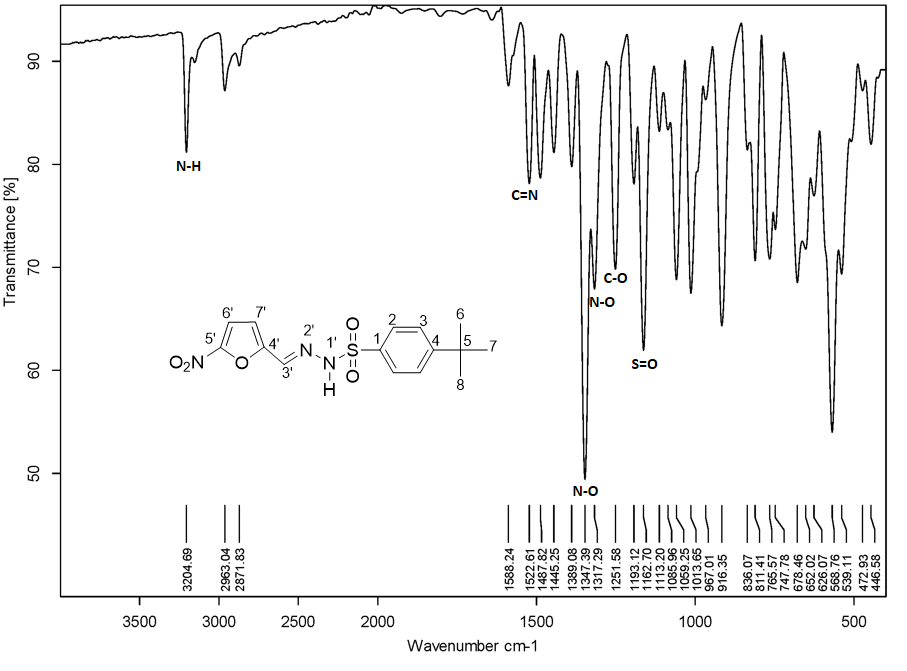
**

**HRMS**


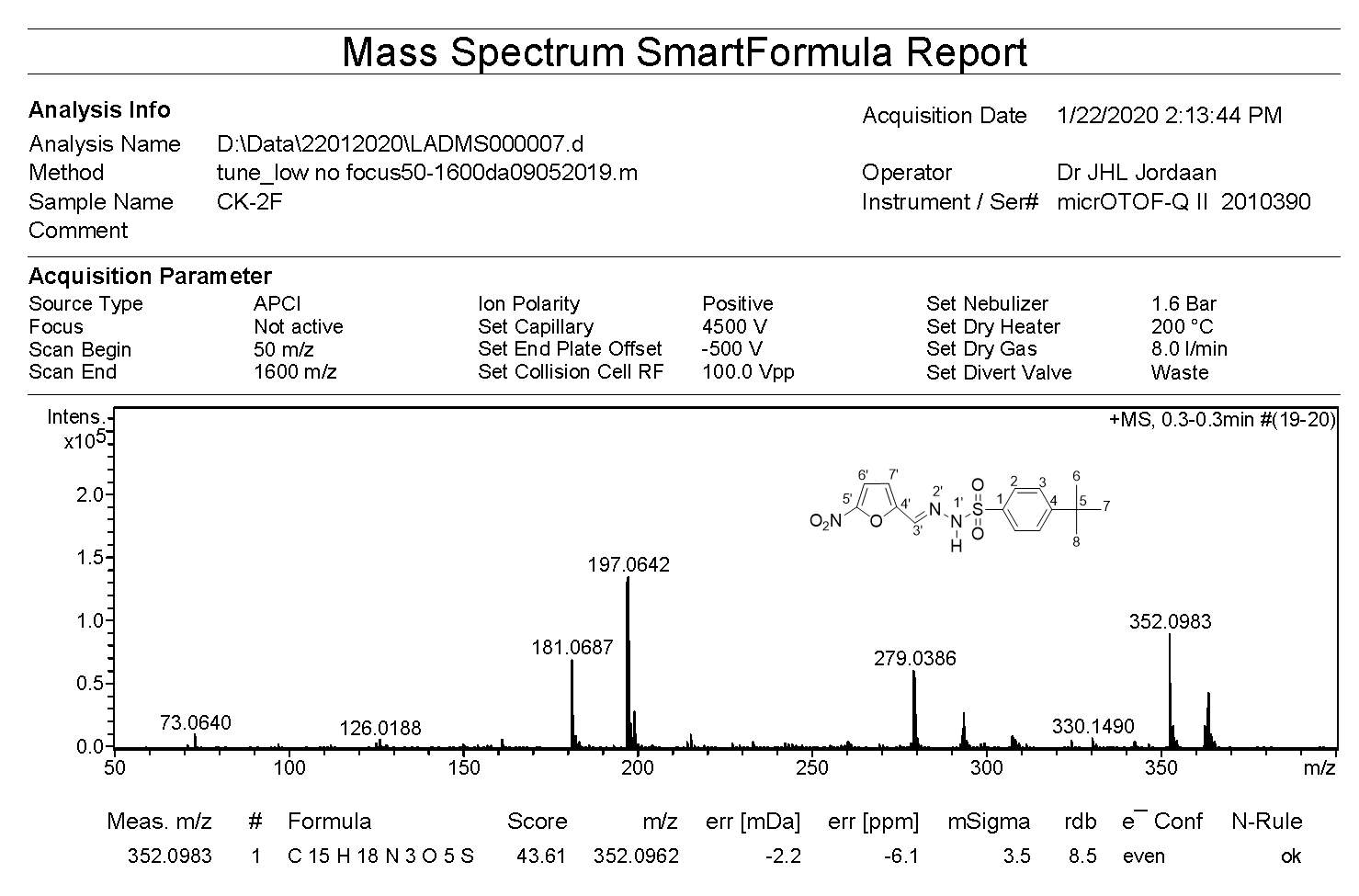


Yellow solid, yield: 90%, mp: 169-171 °C (EtOAc), R*_f_* = 0.50 (EtOAc:*n*-Hex 1:1, v/v ), IR υ_max_ (cm^-1^): 3204 (N-H), 1522 (C=N), 1347 (N-O), 1445 (S=O), 1317 (N-O), 1251 (C-O), 1162 (S=O).^1^H NMR (600 MHz, DMSO) δ 12.19 (s, 1H, H-1'), 7.87 (s, 1H, H-3'), 7.79 (d, *J* = 8.4 Hz, 2H, H-2), 7.72 (d, *J* = 3.9 Hz, 1H, H-6'), 7.65 (d, *J* = 8.4 Hz, 2H, H-3), 7.15 (d, *J* = 3.9 Hz, 1H, H-7'), 1.29 (s, 9H, H-6/7/8). ^13^C NMR (151 MHz, DMSO) δ 156.41 (C-4), 151.81 (C-5'), 150.70 (C-4'), 136.07 (C-1), 134.61 (C-3'), 126.93 (C-2), 126.34 (C-3), 115.40 (C-6'), 114.42 (C-7'), 34.94 (C-5), 30.71 (C-6/7/8). HRMS-APCI (*pos*) *m/z* 352.0983 [M + H] ^+^ (Calcd for C_15_H_18_N_3_O_5_S^+^, 352.0967).

**(*E*)-*N*-{4-{{2-[(5-nitrofuran-2-yl)methylene]hydrazinyl}sulfonyl}phenyl}acetamide (2g)**

**^1^H NMR in DMSO**

**
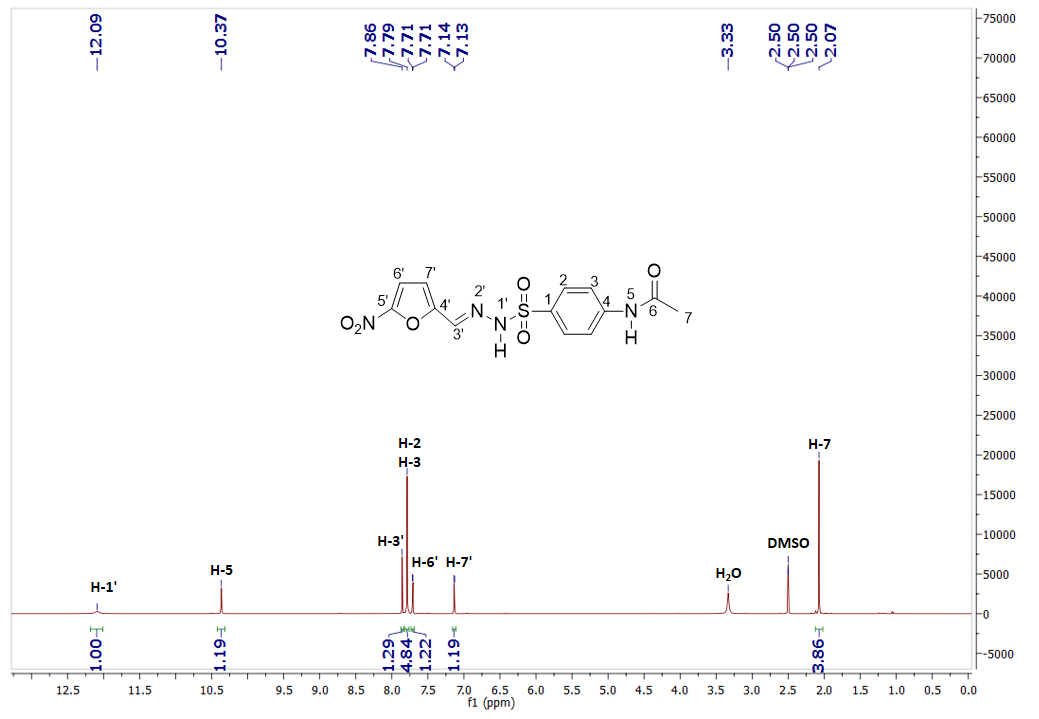
**

**^13^C NMR in DMSO**

**
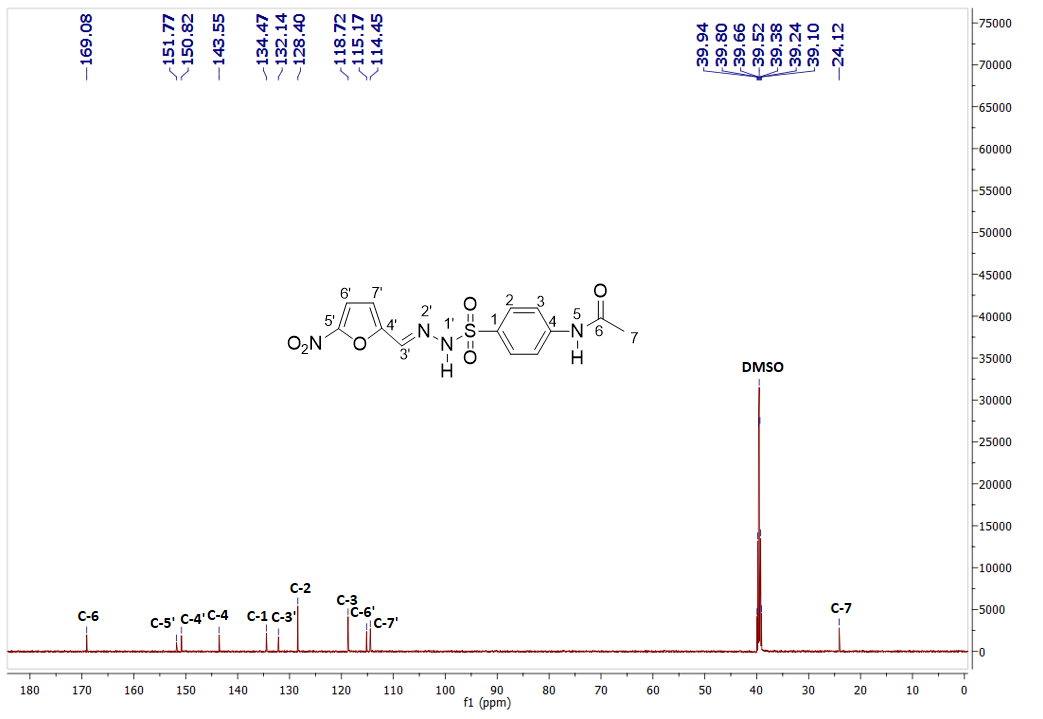
**

**IR Spectrum**

**
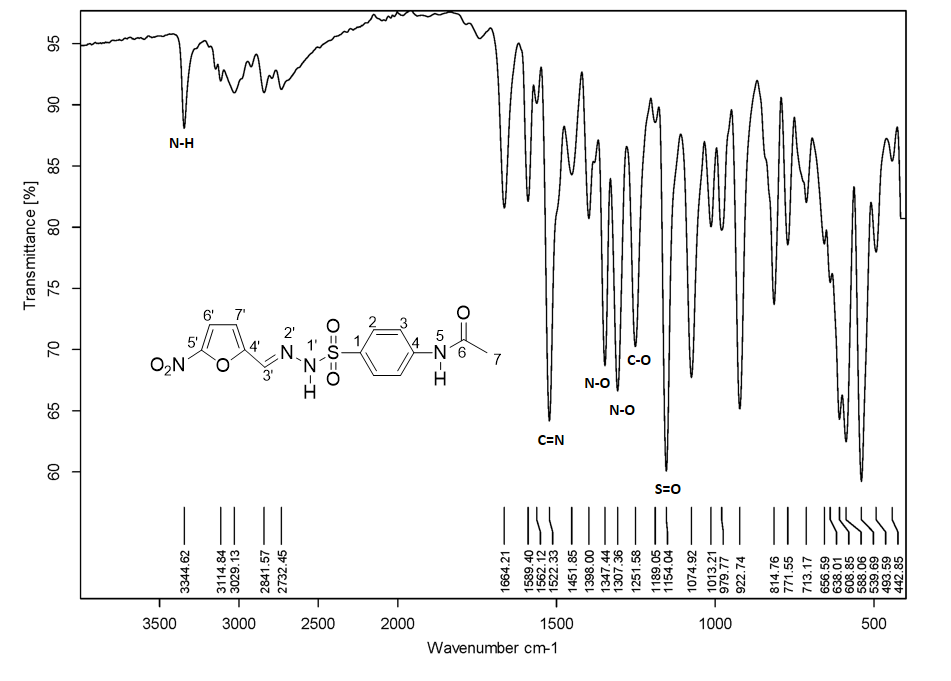
**

**HRMS**

**
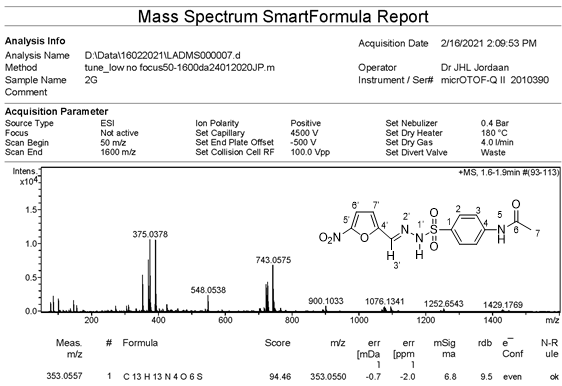
**

Light yellow solid, yield: 96%, mp: 217-220 °C (EtOAc), R*_f_* = 0.97 (EtOAc:*n*-Hex 1:1, v/v ), IR υ_max_ (cm^-1^): 3344 (N-H), 1522 (C=N), 1451 (S=O), 1347 (N-O), 1307 (N-O), 1251 (C-O), 1154 (S=O).^1^H NMR (600 MHz, DMSO) δ 12.09 (s, 1H, H-1'), 10.37 (s, 1H, H-5), 7.86 (s, 1H, H-3'), 7.79 (s, 4H, H-2/3), 7.71 (d, *J* = 3.9 Hz, 1H, H-6'), 7.13 (d, *J* = 3.9 Hz, 1H, H-7'), 2.07 (s, 3H, H-7). ^13^C NMR (151 MHz, DMSO) δ 169.08 (C-6), 151.77 (C-5'), 150.82 (C-4'), 143.55 (C-4), 134.47 (C-1), 132.14 (C-3'), 128.40 (C-2), 118.72 (C-3), 115.17 (C-6'), 114.45 (C-7'), 24.12 (C-7). HRMS-ESI (*pos*) *m/z* 353.0550 [M + H] ^+^ (calcd for C_13_H_13_N_4_O_6_S^+^, 353.0556).

**(*E*)-*N'*-[(5-Nitrofuran-2-yl)methylene]-(1,1'-biphenyl)-4-sulfonohydrazide (2h)**

**^1^H NMR in DMSO**

**
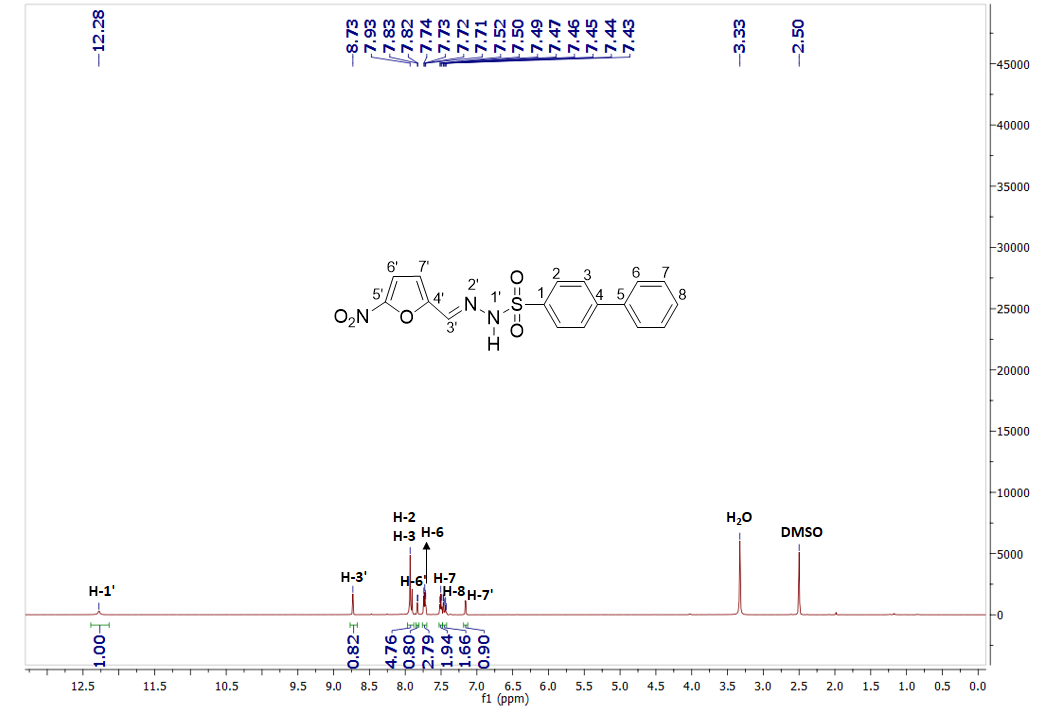
**

**^13^C NMR in DMSO**

**
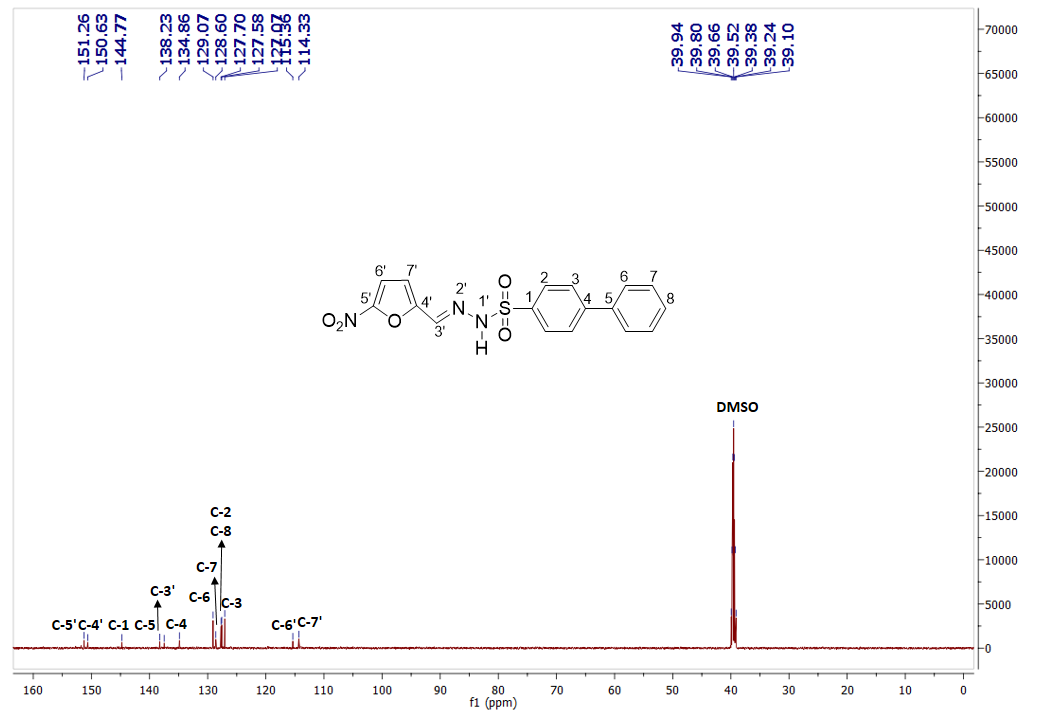
**

**IR Spectrum**

**
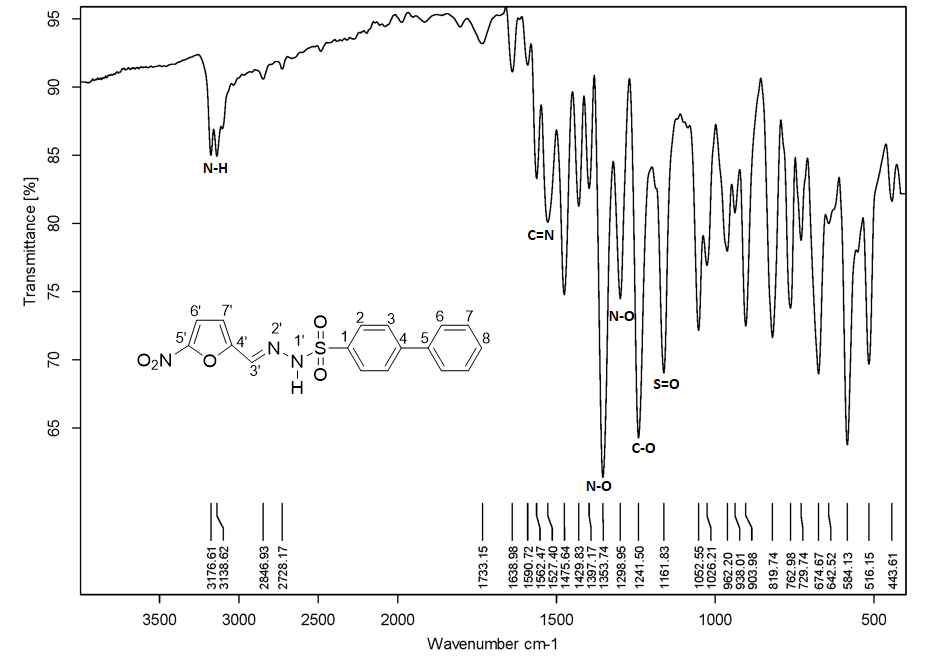
**

**HRMS**


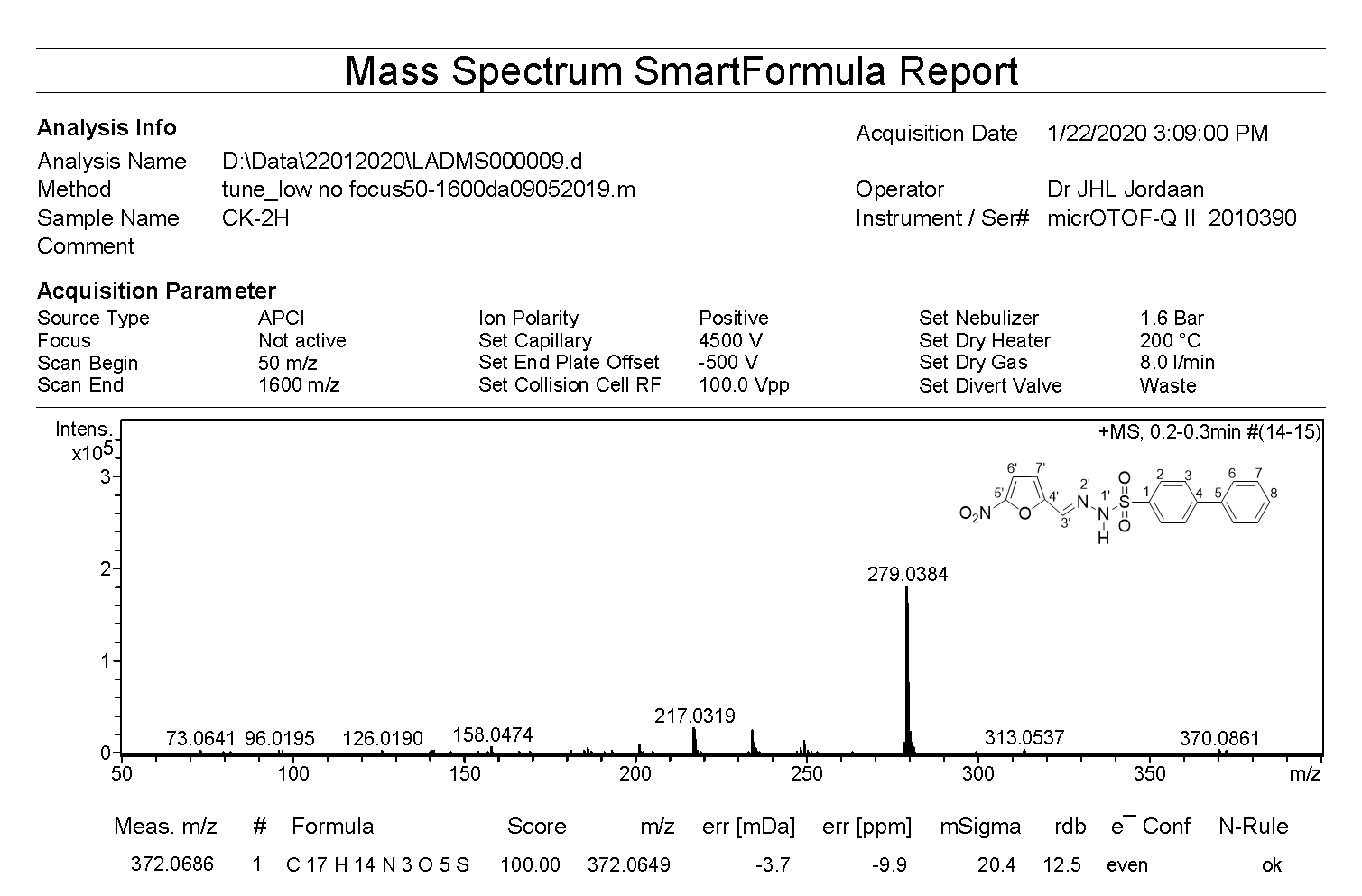


Yellow solid, yield: 92%, mp: 155-158 °C (EtOAc), R*_f_* = 0.40 (EtOAc:*n*-Hex 1:1, v/v ), IR υ_max_ (cm^-1^): 3138 (N-H), 1562 (C=N), 1475 (S=O), 1353 (N-O), 1298 (N-O), 1241 (C-O), 1161 (S=O). ^1^H NMR (600 MHz, DMSO) δ 12.28 (s, 1H, H-1'), 8.73 (s, 1H, H-3'), 7.93 (s, 4H, H-2/3), 7.83 (d, *J* = 3.8 Hz, 1H, H-6'), 7.73 (dd, *J* = 9.6, 5.8 Hz, 2H, H-6), 7.50 (t, *J* = 7.6 Hz, 2H, H-7), 7.46 (dd, *J* = 13.7, 5.6 Hz, 1H, H-8), 7.16 (d, *J* = 3.8 Hz, 1H, H-7'). ^13^C NMR (151 MHz, DMSO) δ 151.26 (C-5'), 150.63 (C-4'), 144.77 (C-1), 138.23 (C-5), 137.48 (C-3'), 134.86 (C-4), 129.07 (C-6), 128.60 (C-7), 127.70 (C-2), 127.58 (C-8), 127.07 (C-3), 115.36 (C-6'), 114.33 (C-7'). HRMS-APCI (*pos*) *m/z* 372.0686 [M + H] ^+^ (Calcd for C_17_H_14_N_3_O_5_S^+^, 372.0654).

***(E)*-4-Fluoro-*N'*-([5-nitrofuran-2-yl]methylene)-(1,1'-biphenyl)-4-sulfonohydrazide (2i)**

**^1^H NMR in DMSO**

**
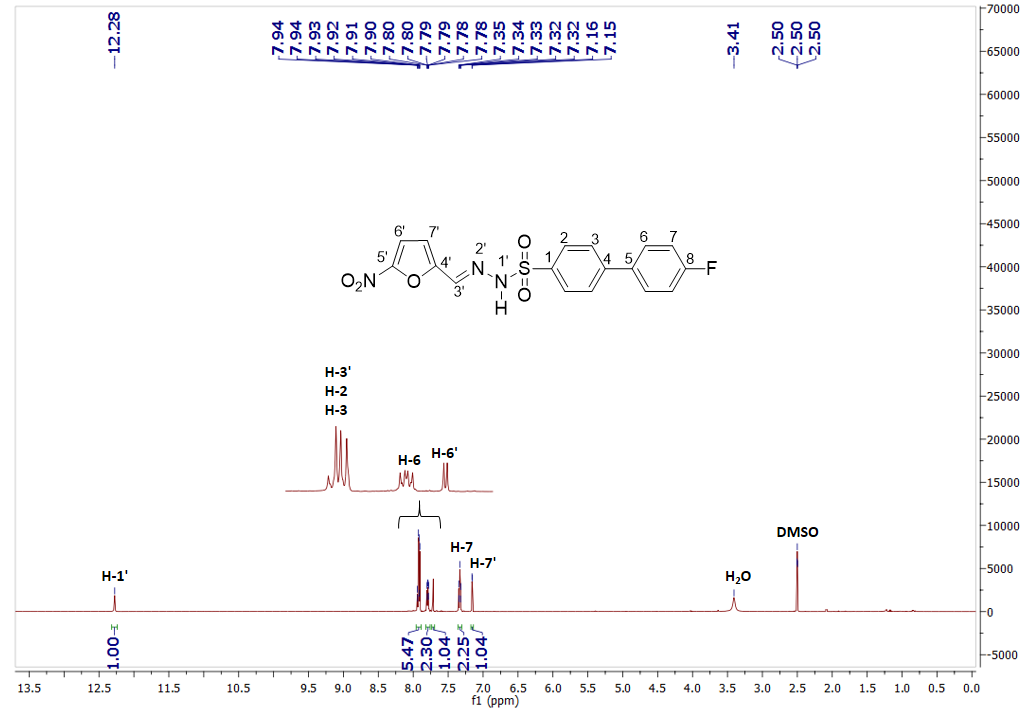
**

**^13^C NMR in DMSO**

**
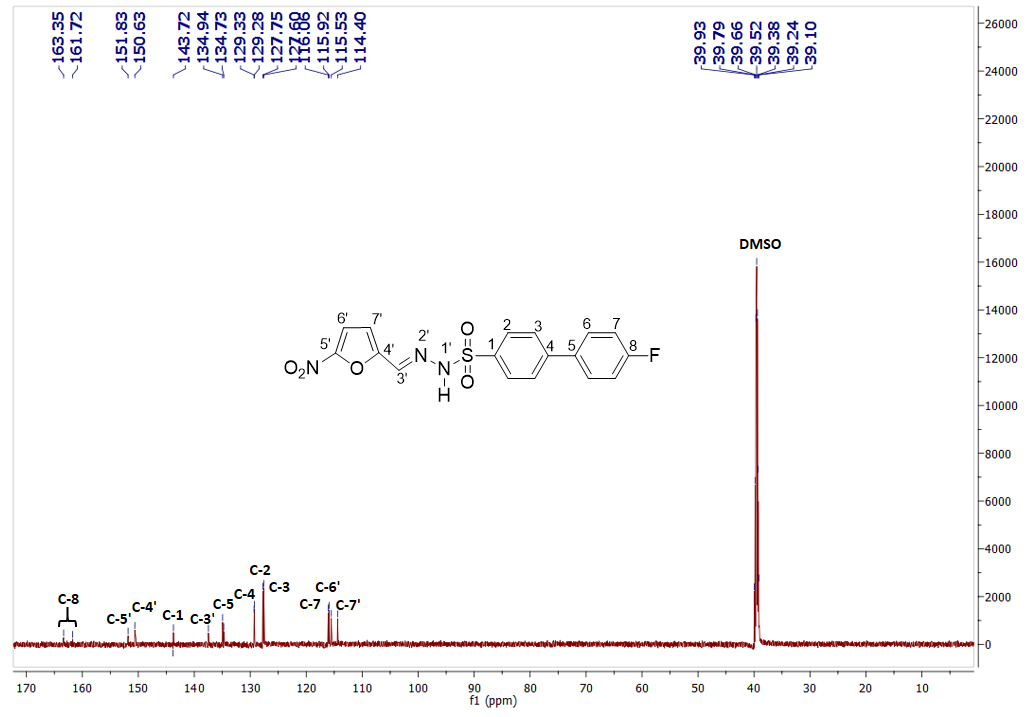
**

**IR Spectrum**

**
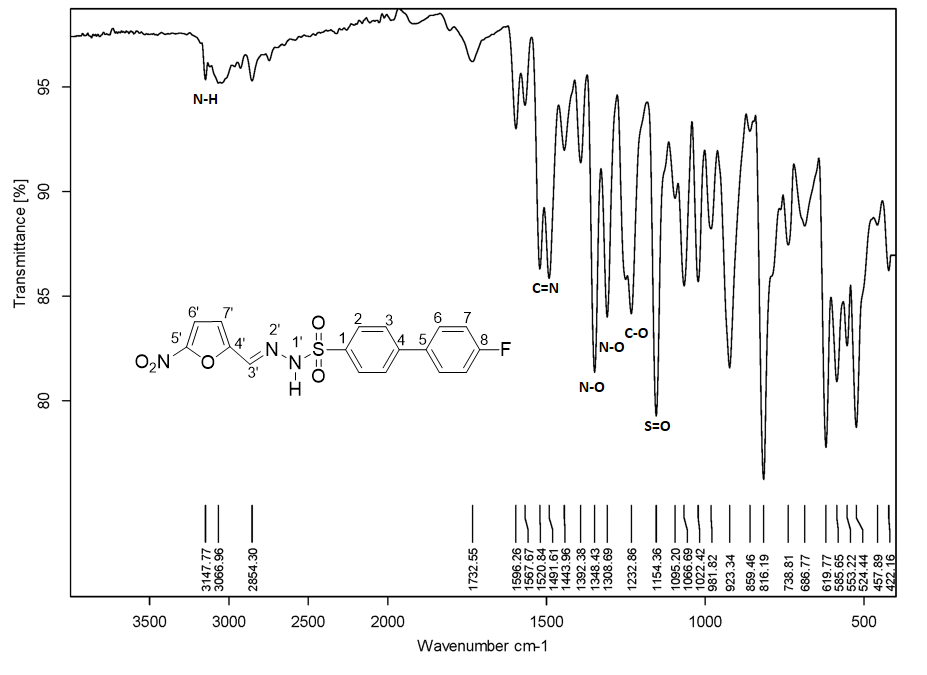
**

**HRMS**

**
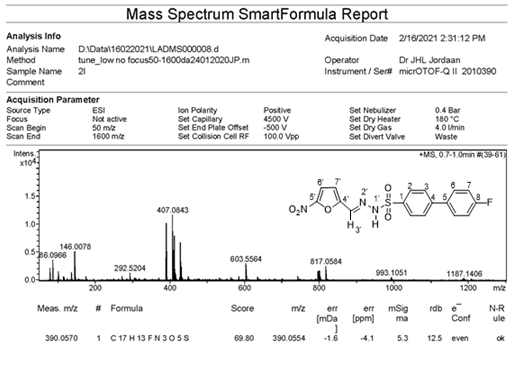
**

Light brown solid, yield: 94%, mp: 155-158 °C (EtOAc), R*_f_* = 0.34 (EtOAc:*n*-Hex 1:1, v/v ), IR υ_max_ (cm^-1^): 3147 (N-H), 1567 (C=N), 1443 (S=O), 1392 (N-O), 1348 (N-O), 1232 (C-O), 1154 (S=O). ^1^H NMR (600 MHz, DMSO) δ 12.28 (s, 1H, H-1'), 7.94 – 7.90 (m, 5H, H-3'/2/3), 7.79 (dd, *J*_H-H_ = 8.8, ^3^*J*_H-F_ = 5.4 Hz, 2H, H-7), 7.72 (d, *J* = 3.9 Hz, 1H, H-6'), 7.33 (d, *J* = 8.8 Hz, 2H, H-6), 7.16 (d, *J* = 3.9 Hz, 1H, H-7'). ^13^C NMR (151 MHz, DMSO) δ 162.53 (d, ^1^*J* _C-F_= 245.4 Hz, C-8), 151.84 (C-5'), 150.63 (C-4'), 143.72 (C-1), 137.48 (C-3'), 134.94 (C-5), 134.73 (C-4), 129.30 (d, ^3^*J*_C-F_ = 7.7 Hz, C-6), 127.75 (C-2), 127.60 (C-3), 115.99 (d, ^2^*J*_C-F_ = 21.7 Hz, C-7), 115.53 (C-6'), 114.40 (C-7'). HRMS-ESI (*pos*) *m/z* 390.0570 [M + H] ^+^ (Calcd for C_17_H_13_FN_3_O_5_S^+^, 390.0560).

**(*E*)-4-Chloro-*N'*-[(5-nitrofuran-2-yl)methylene]-(1,1'-biphenyl)-4-sulfonohydrazide (2j)**

**^1^H NMR in DMSO**

**
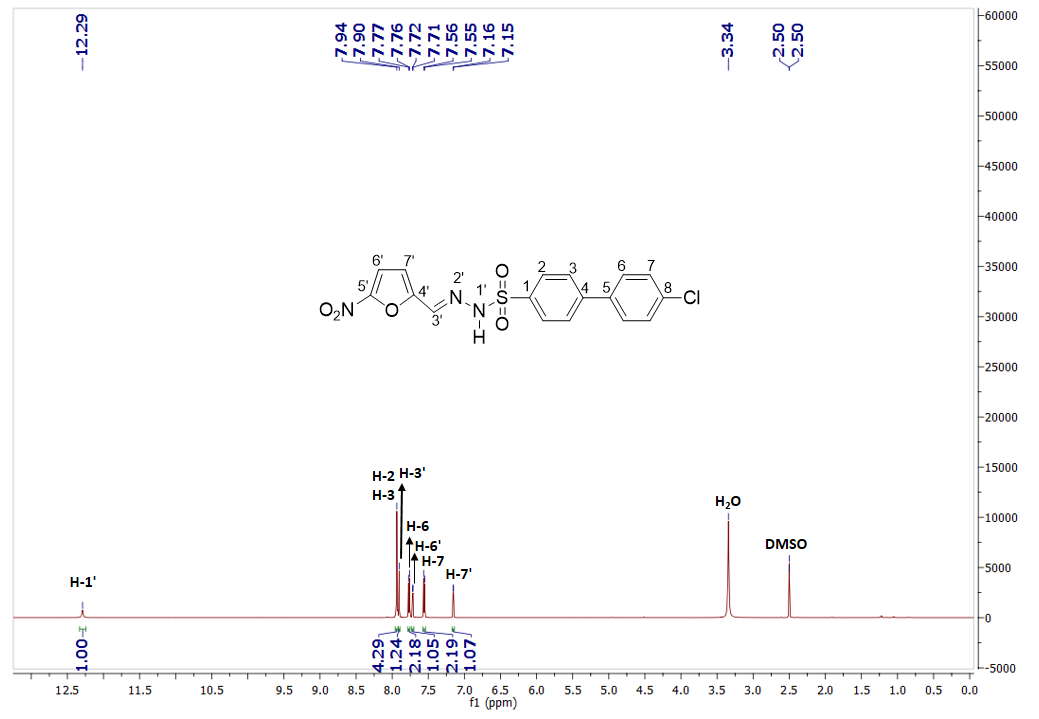
**

**^13^C NMR in DMSO**

**
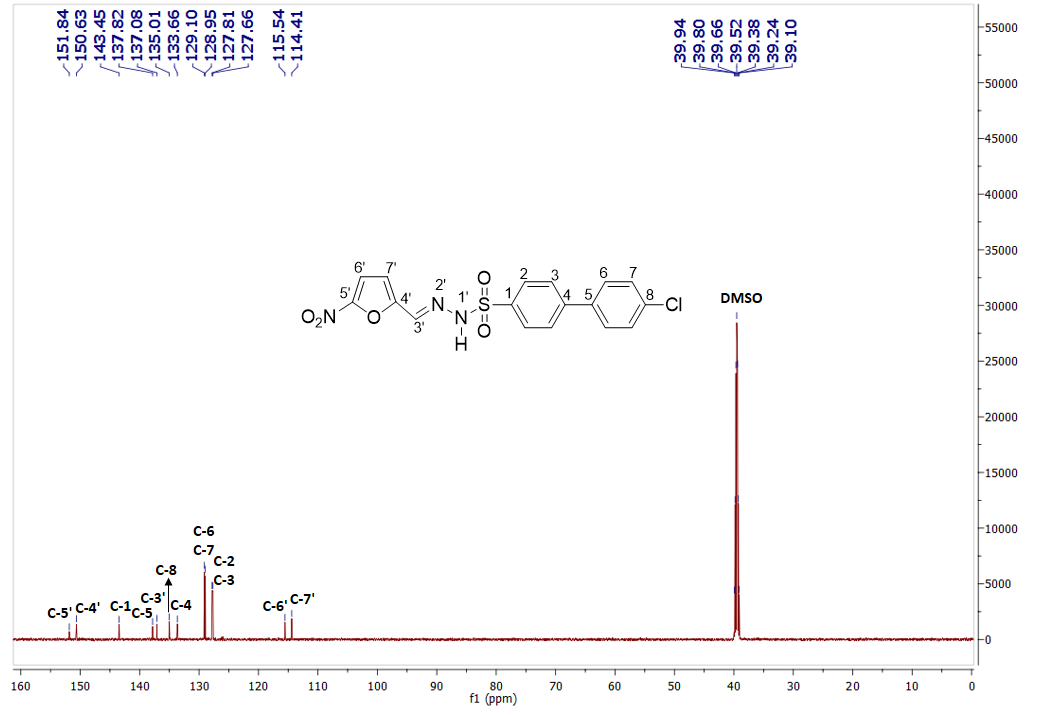
**

**IR Spectrum**

**
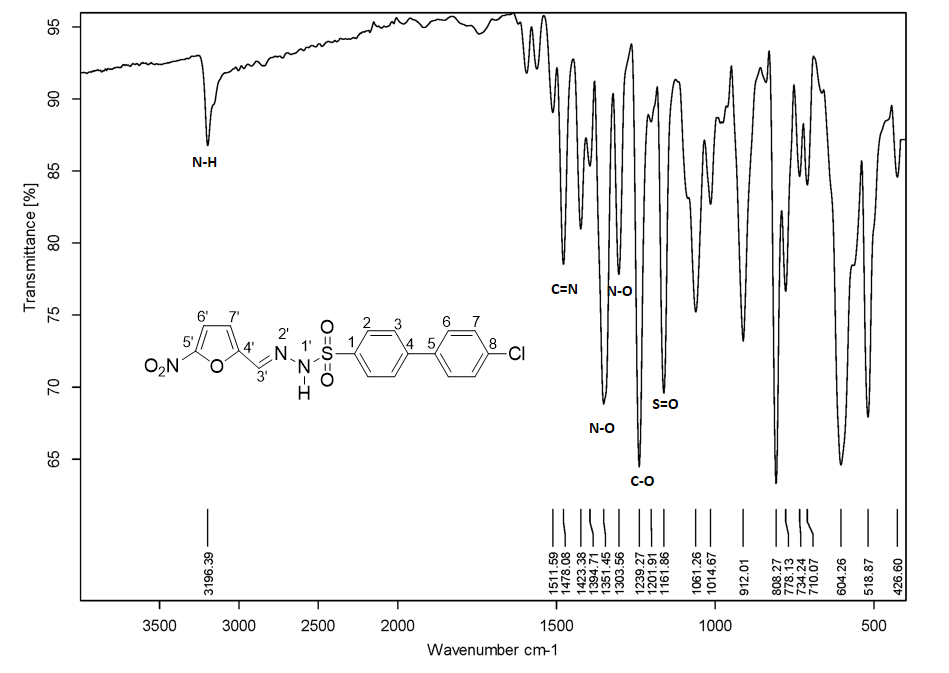
**

**HRMS**


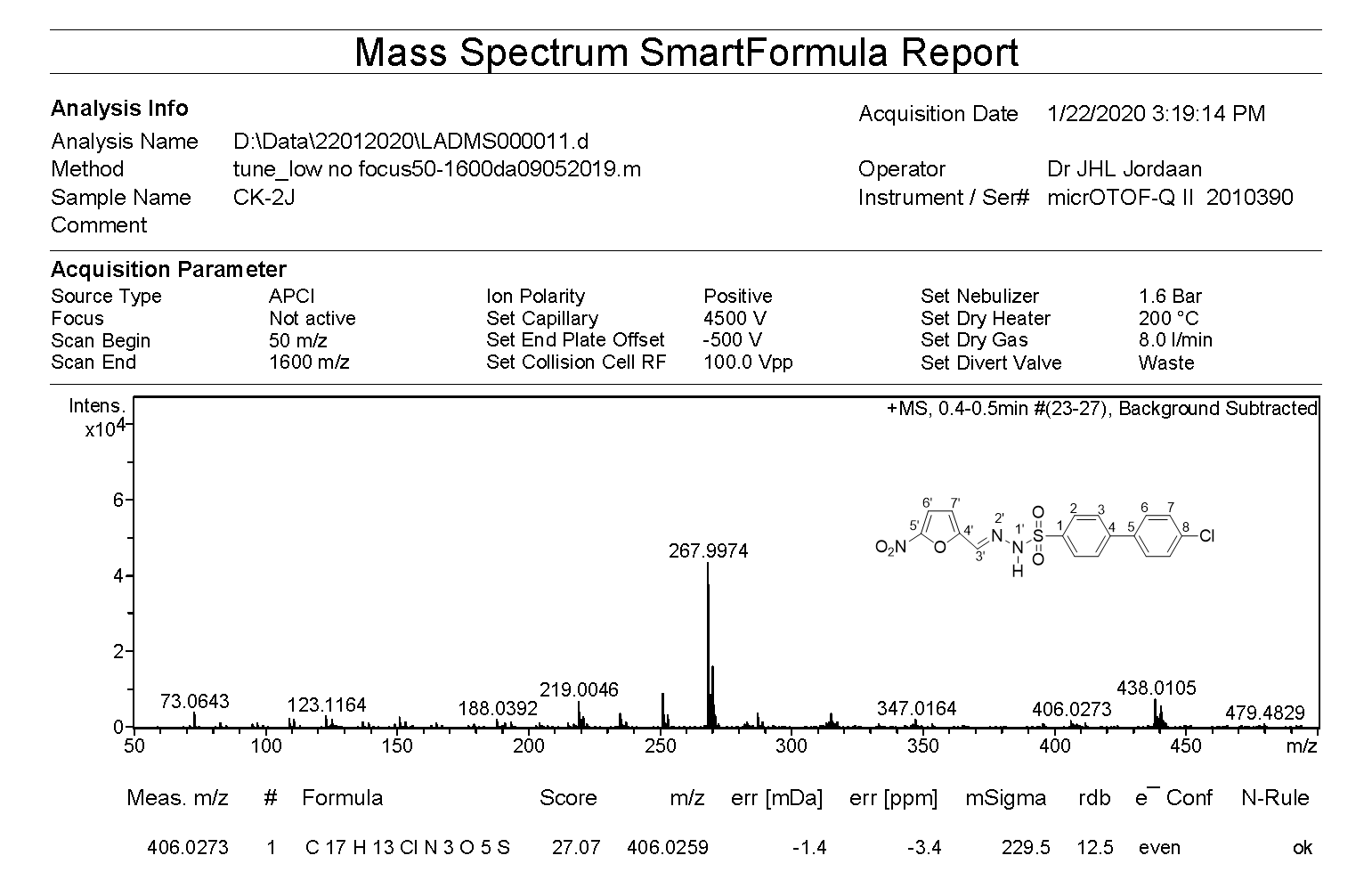


Yellow solid, yield: 95%, mp: 179-182 °C (EtOAc), R*_f_* = 0.34 (EtOAc:*n*-Hex 1:1, v/v ), IR υ_max_ (cm^-1^): 3196 (N-H), 1511 (C=N), 1478 (S=O), 1394 (N-O), 1303 (N-O), 1239 (C-O), 1161 (S=O). ^1^H NMR (600 MHz, DMSO) δ 12.29 (s, 1H, H-1'), 7.94 (s, 4H, H-2/3), 7.77 (d, *J* = 8.5 Hz, 2H, H-3'), 7.71 (d, *J* = 3.9 Hz, 1H, H-6'), 7.56 (d, *J* = 8.5 Hz, 2H, H-7), 7.15 (d, *J* = 3.9 Hz, 1H H-7'). ^13^C NMR (151 MHz, DMSO) δ 151.84 (C-5'), 150.63 (C-4'), 143.45 (C-1), 137.82 (C-5), 137.08 (C-3'), 135.01 (C-8), 133.66 (C-4), 129.10 (C-6), 128.95 (C-7), 127.81 (C-2), 127.66 (C-3), 115.54 (C-6'), 114.41 (C-7'). HRMS-APCI (*pos*) *m/z* 406.0273 [M + H] ^+^ (Calcd for C_17_H_13_ClN_3_O_5_S^+^, 406.0264).

**(*E*)-4-Methoxy-*N'*-[(5-nitrofuran-2-yl)methylene]-(1,1'-biphenyl)-4-sulfonohydrazide (2k)**

**^1^H NMR in DMSO**

**
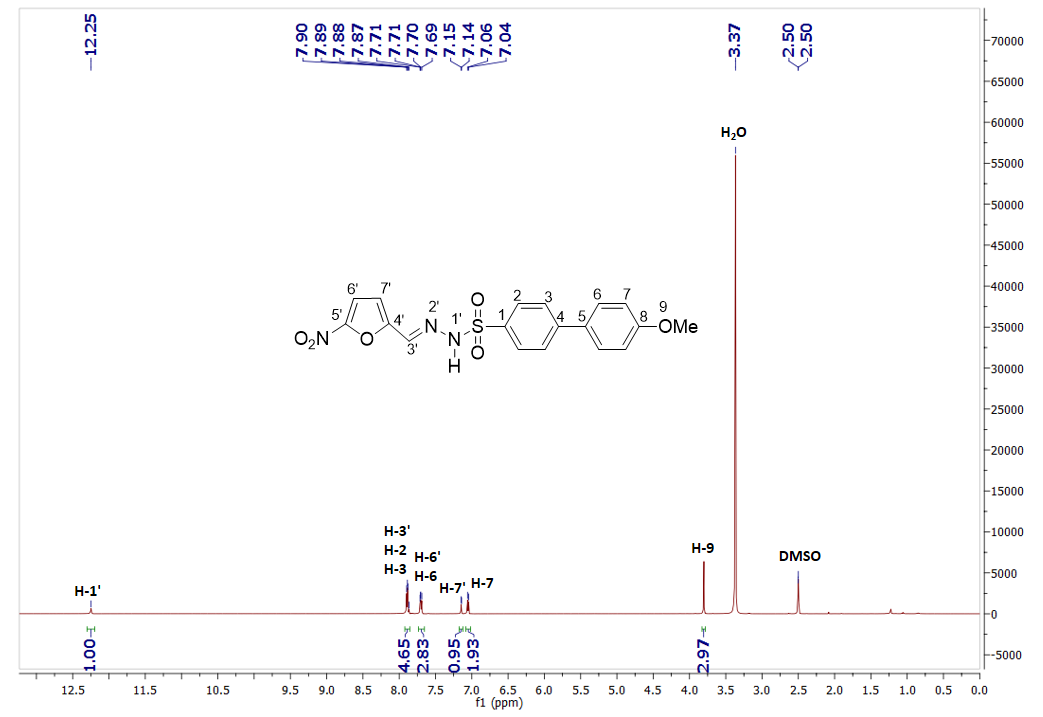
**

**^13^C NMR in DMSO**

**
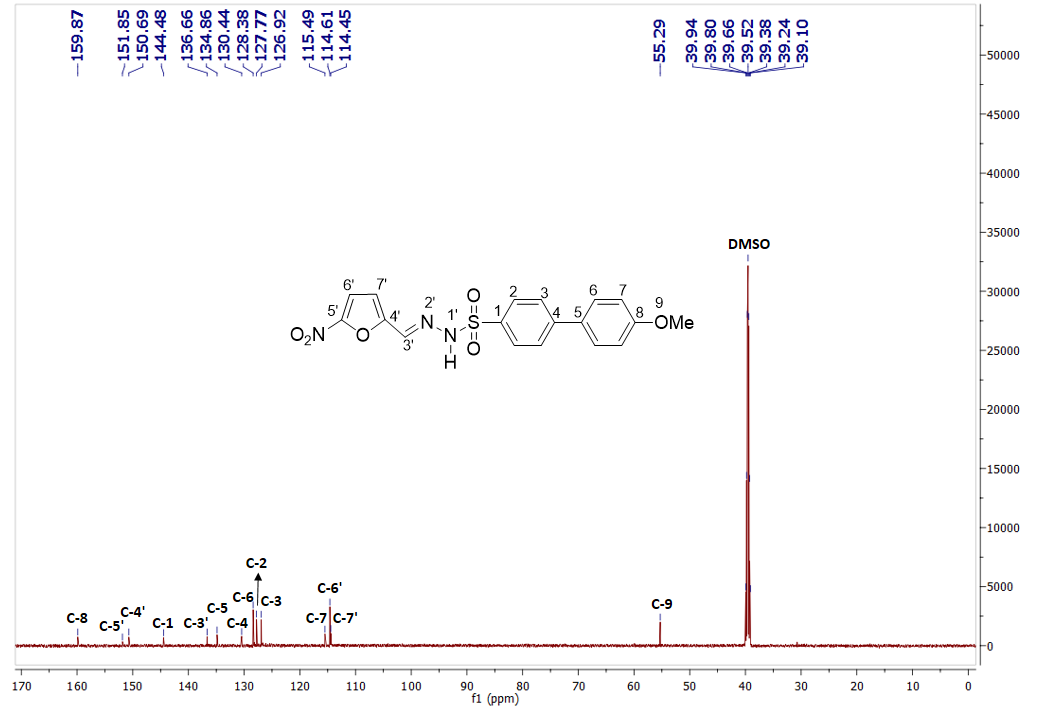
**

**IR Spectrum**

**
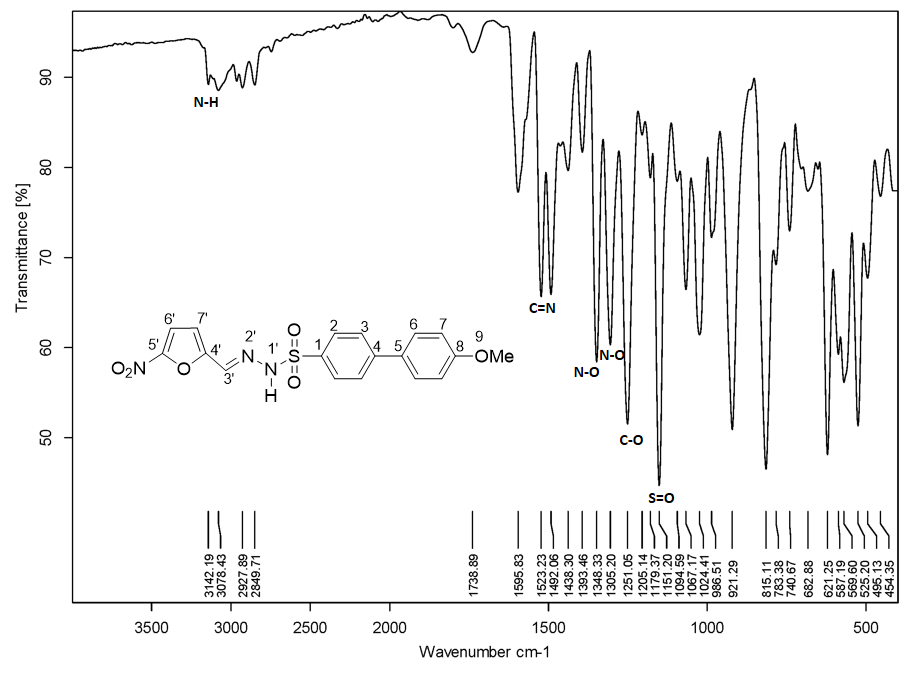
HRMS**

**
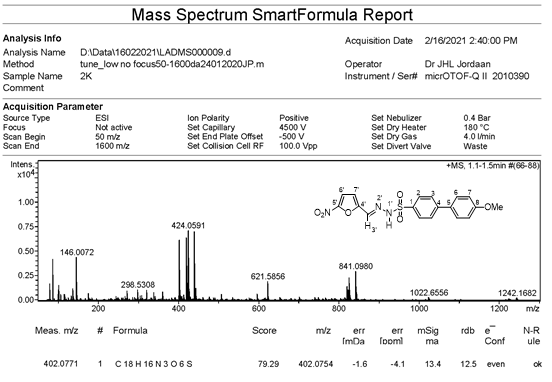
**

White solid, yield: 96%, mp: 167-170 °C (EtOAc), R*_f_* = 0.34 (EtOAc:*n*-Hex 1:1, v/v ), IR υ_max_ (cm^-1^): 3142 (N-H), 1522 (C=N), 1438 (S=O), 1393 (N-O), 1305 (N-O), 1251 (C-O), 1179 (S=O). ^1^H NMR (600 MHz, DMSO) δ 12.25 (s, 1H, H-1'), 7.88 (dd, *J* = 11.5, 7.4 Hz, 5H, H-3'/2/3), 7.70 (d, *J* = 8.8 Hz, 2H, H-6), 7.14 (d, *J* = 3.9 Hz, 1H, H-6'), 7.14 (d, *J* = 3.9 Hz, 1H, H-7'), 7.05 (d, *J* = 8.8 Hz, 2H, H-7), 3.80 (s, 3H, H-9). ^13^C NMR (151 MHz, DMSO) δ 159.87 (C-8), 151.85 (C-5'), 150.69 (C-4'), 144.48 (C-1), 136.66 (C-3'), 134.86 (C-5), 130.44 (C-4), 128.38 (C-6), 127.77 (C-2), 126.92 (C-3), 115.49 (C-7), 114.61 (C-6'), 114.45 (C-7'), 55.29 (C-9). HRMS-ESI (*pos*) *m/z* 402.0771 [M + H] ^+^ (Calcd for C_18_H_16_N_3_O_6_S^+^, 402.0760).

**(*E*)-*N'*-[(5-Nitrofuran-2-yl)methylene]-2,3-dihydrobenzo[*b*][1,4]dioxine-5-sulfonohydrazide (2l)**

**^1^H NMR in DMSO**

**
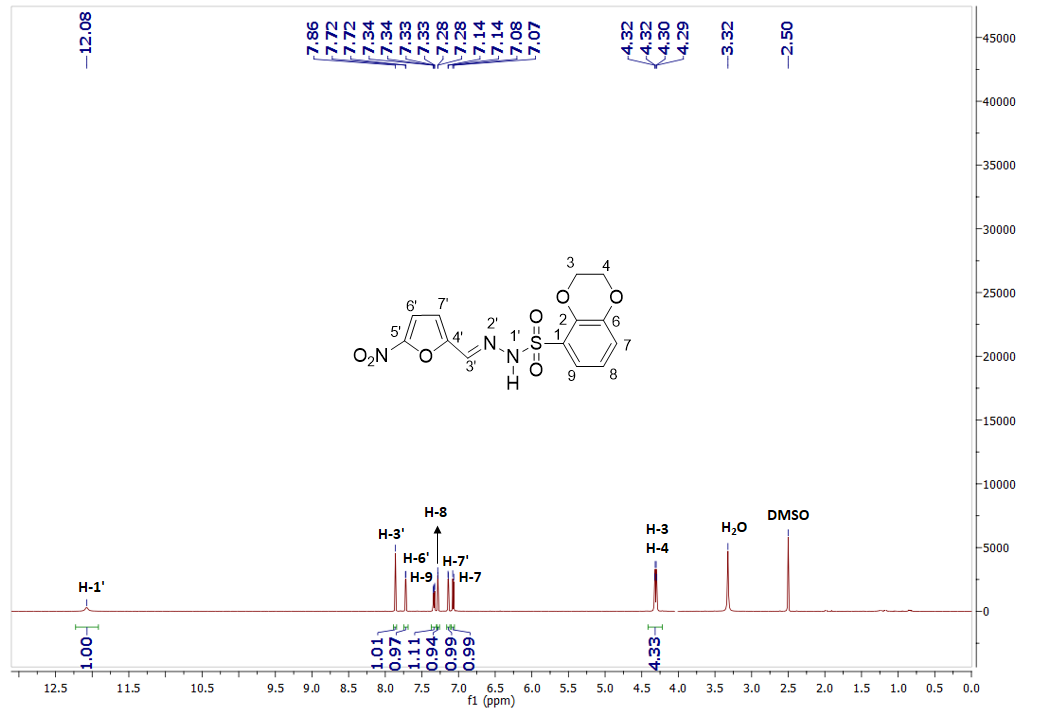
**

**^13^C NMR in DMSO**

**
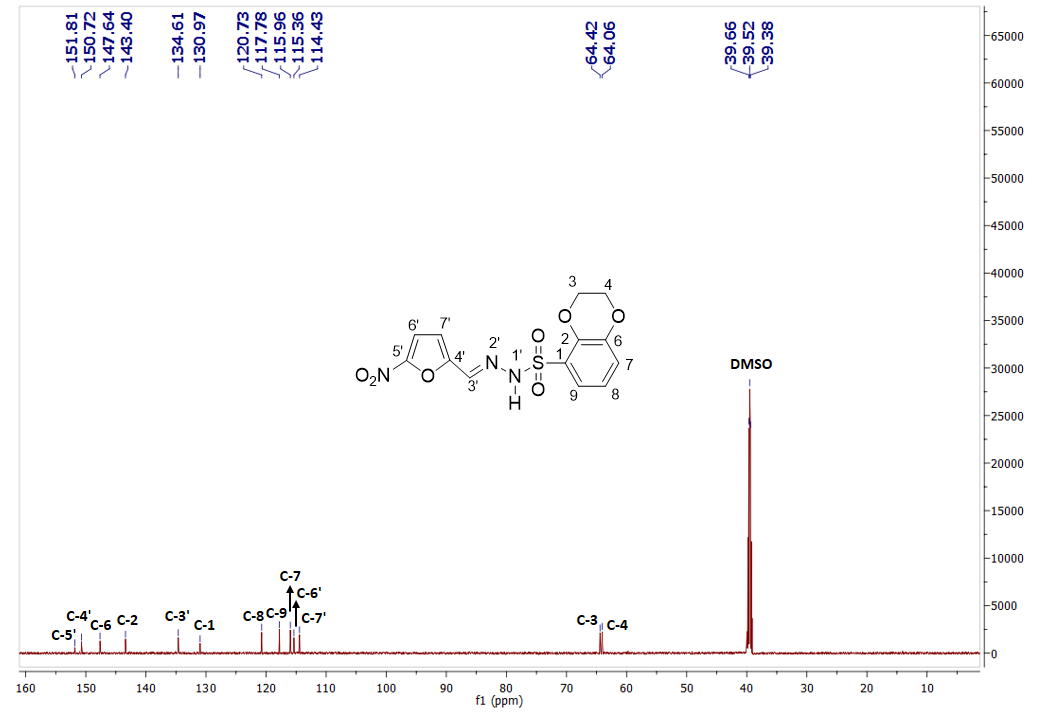
**

**IR Spectrum**

**
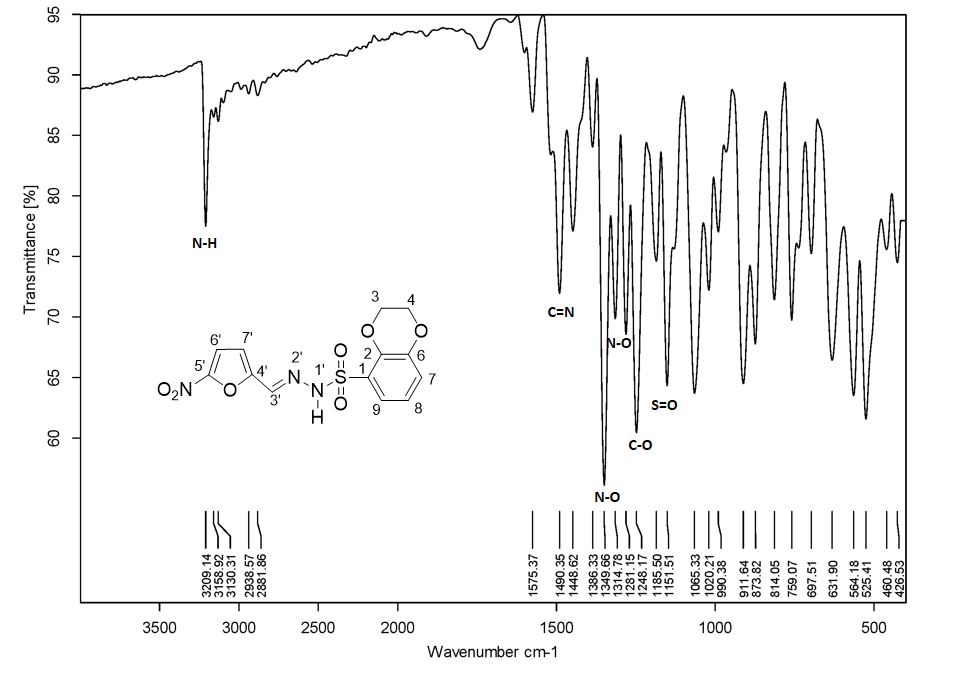
**

**HRMS**

**
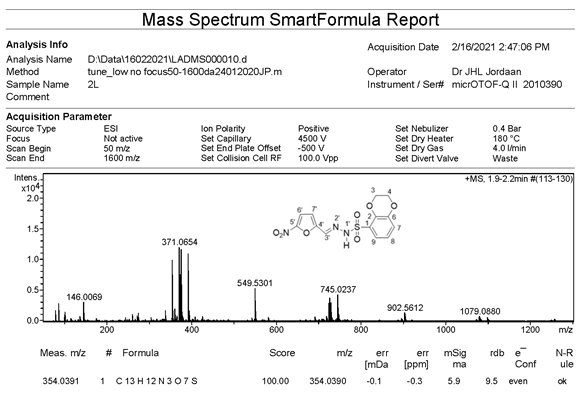
**

Light yellow solid, yield: 92%, mp: 163-166 °C (EtOAc), R*_f_* = 0.26 (EtOAc:*n*-Hex 1:1, v/v ), IR υ_max_ (cm^-1^): 3209 (N-H), 1490 (C=N), 1448 (S=O), 1349 (N-O), 1314 (N-O), 1248 (C-O), 1185 (S=O).^1^H NMR (600 MHz, DMSO) δ 12.08 (s, 1H, H-1'), 7.86 (s, 1H, H-3'), 7.72 (d, *J* = 3.9 Hz, 1H, H-6'), 7.33 (dd, *J* = 8.5, 2.2 Hz, 1H, H-9), 7.28 (d, *J* = 2.1 Hz, 1H, H-8), 7.14 (d, *J* = 3.9 Hz, 1H, H-7'), 7.07 (d, *J* = 8.5 Hz, 1H, H-7'), 4.31 (dd, *J* = 13.0, 5.0 Hz, 4H, H-3/4). ^13^C NMR (151 MHz, DMSO) δ 151.81 (C-5'), 150.72 (C-4'), 147.64 (C-6), 143.40 (C-2), 134.61 (C-3'), 130.97 (C-1), 120.73 (C-8), 117.78 (C-9), 115.96 (C-7), 115.36 (C-6'), 114.43 (C-7'), 64.42 (C-3), 64.06 (C-4). HRMS-ESI (*pos*) *m/z* 354.0391 [M + H] ^+^ (Calcd for C_13_H_12_N_3_O_7_S^+^, 354.0396).

***In vitro* biological assays**

***Antipromastigote assay***

The antipromastigote activity of synthesized compounds was evaluated using a modified, resazurin-based method of Kulshrestha *et al.* ^2^ and Siqueira-Neto *et al.* ^3^ and on three *Leishmania* strains. *Leishmania donovani* (strains 1S (MHOM/SD/62/1S) and 9515 (MHOM/IN/95/9515)) and *L. major* (strain IR-173 (MHOM/IR/-173)) promastigotes were cultured in M199 with Hank's salts and 0.68 mM L-glutamine (Sigma Aldrich) supplemented with 50 U/mL Penicillin/Streptomycin solution (Lonza), 4.2 mM sodium bicarbonate, 25 mM Hepes, 10% fetal bovine serum, 0.25% hemin, 0.1% biotin, and 10 mM adenine (Sigma Aldrich) and the pH adjusted to 7.3 – 7.4. The promastigotes were maintained at 25 °C.

For the resazurin assay, logarithmic phase promastigotes (1.25 x 10^6^ cells/mL, final volume 100 μL/well) were seeded in 96 well plates (Nunc, Thermofisher Scientific) in the presence of: (i) 10 μM of compound for activity screening or (ii) 7 two-fold dilution concentrations of 10 μM compounds for IC_50_ determination. Amphothericin B (10 μM) served as the standard drug and growth medium without parasites served as the blank. The plates were incubated for 48 hours at 25 ˚C in humidified atmosphere. After incubation, 50 μL of resazurin solution (0.01% in PBS) was added to each well and the plates were further incubated at 25 ˚C in the dark for 24 hours. Absorbance was measured at 570 nm and 600 nm using the Thermofisher Scientific GO Multiscan plate reader. Data analysis was performed for each biological replicate using SkanIt 4.0 Research Edition software. Background absorbance of resazurin (600 nm) was subtracted from the absorbance values of resorufin (570 nm). The mean absorbance calculated and the percentage growth inhibition and cell viability were determined by the following equations:

$$Growth inhibition \% = \frac{(\Delta Abs neg control - \Delta Abs blank) - (\Delta Abs sample - \Delta Abs blank)}{(\Delta Abs neg control - \Delta Abs blank)}\times100$$

$$Cell viability \% =\frac{(\Delta Abs sample - \Delta Abs blank)}{(\Delta Abs neg control - \Delta Abs blank)}\times100$$

All compounds were first screened for >70% growth inhibition at 10 μM ^3^ and qualifying compounds were further used for IC_50_ determinations. The IC_50_ and Z-score were determined for each compound’s three biological replicates using the cell viability % values and GraphPad Prism 5. The mean IC_50_ of the biological replicates with standard deviation (SD) served as the final IC_50_ of each compound.

***Cytotoxicity***

African green monkey kidney epithelial (Vero) cells (Cellonex, South Africa) were cultured and the basal cytotoxicity of the synthesized compounds with antileishmanial and/or anticancer activity was evaluated using the resazurin assay, as described in Mangwegape *et al*. ^4^ Emetine served as the standard cytotoxic drug. Further use of reference drugs, controls, data analysis, calculations and IC_50_ determinations were identical to that of the antileishmanial assay.

Cytotoxicity of the compounds on the differentiated host THP-1 cells were also evaluated. As mentioned, duplicate plates of differentiated THP-1 cells were prepared during the anti-amastigote assay. These plates were incubated for 72 hours to differentiate, instead of replacing the growth medium with parasite-containing medium after 48 hours. The plates were then treated with 200 μL of: (i) amphotericin B (Sigma Aldrich) (positive control); (ii) growth medium and solvent (negative control to compensate for possible solvent effects); (iii) 7 two-fold dilution concentrations of 100 μM compounds for IC_50_ determination. Blanks were growth medium without cells. The treated plates were incubated for 72 hours, followed by the addition of 50 μL of resazurin solution (0.01% in PBS) to each well and 24-hour incubation. Further data analysis, calculations and IC_50_ determinations were identical to that of the antileishmanial assays.

**Anticancer assay: MCF7 and A549 cell lines**

The potential anticancer activities of synthesized compounds were screened using the resazurin assay. ^5^ Breast adenocarcinoma (MCF7, ATCC® HTB-22™) and human melanoma (A375, ATCC® CRL-1619™) cells were cultured in Hyclone Dulbecco's modified Eagle’s medium with high glucose (Separations) supplemented with 10 % fetal bovine serum (FBS) (Thermofisher Scientific) and 1 % L-glutamine, penicillin-streptomycin and non-essential amino acids (Lonza). Human lung carcinoma (A549, ATCC®, CCL-185™) cells were cultured in Ham’s F12K medium supplemented with 10% FBS and 1% L-glutamine, penicillin-streptomycin and non-essential amino acids. Human promyelocytic leukemia (Clone 15 HL-60, ATCC® CRL-1946™) cells were cultured in RPMI-1640 medium (Sigma Aldrich) supplemented with 10% FBS and 1% penicillin-streptomycin. The cell lines were maintained in a humidified atmosphere at 37 °C and 5% CO_2_.

For the resazurin assay, 96 well plates were prepared with 100 μL of 100 000 cells/mL MCF7 cell suspension, 100 μL of 50 000 cells/mL A375 cell suspension, 100 μL of 25 000 cells/mL A549 cell suspension, or 50 μL of 50 000 cells/mL HL-60 cell suspension. The MCF7, A375 and A549 cell plates were incubated for 24 hours to allow adherence of the cells to the plate surface, after which the growth medium is replaced with 100 μL medium containing compounds. The HL-60 cell line is a suspension cell line that does not require the 24-hour incubation period for adherence and the wells with cell suspension (50 μL/well) were treated on the same day by adding 50 μL of medium containing compounds. The cell lines were treated with: (i) parthenolide (Sigma Aldrich) (positive control); (ii) growth medium and solvent (negative control to compensate for possible solvent effects); (iii) growth medium and experimental compound solutions. Concentrations used: (i) 20 μM of compound for activity screening or (ii) 7 two-fold dilution concentrations of 20 μM compounds for IC_50_ determination. Blanks contained growth medium without cells. The treated plates were incubated for 48 hours.

To initiate the resazurin assay, 50 μL of sterile-filtered resazurin sodium salt (Sigma Aldrich) solution (0.01 % in PBS) was added and the plates incubated for 2 hours (MCF7, A375 and A549 cells) or 24 hours (HL-60 cells). Absorbance was measured at 570 and 600 nm using the Thermofisher Scientific GO Multiscan plate reader. Data analysis was performed for each biological replicate using SkanIt 4.0 Research Edition software. Further data analysis, calculations and IC_50_ determinations were identical to that of the antileishmanial and cytotoxicity assays.

All compounds were screened for growth inhibition >50 % at 20 μM and compounds that qualified were further used for IC_50_ determination. The IC_50_ and Z-score were determined for each qualifying compound’s biological replicate using GraphPad Prism 5. For the final IC_50_ of each compound, the mean IC_50_ of the biological replicates were calculated with standard deviation (SD).

**References**

1. H. Alsaeedi, N. Aljaber and I. Ara, *Asian J. Chem.*, 2015, **27**, 3639-3646.

2. A. Kulshrestha, V. Bhandari, R. Mukhopadhyay, V. Ramesh, S. Sundar, L. Maes, J. C. Dujardin, S. Roy and P. Salotra, *Parasitology research*, 2013, **112**, 825-828.

3. J. L. Siqueira-Neto, O. Song, H. Oh, J. Sohn, G. Yang, J. Nam, J. Jang, J. Cechetto, C. B. Lee, S. Moon, A. Genovesio, E. Chatelain, T. Christophe and L. H. Freitas-Junior, *PLoS Negl Trop Dis*, 2010, **4**, e675-e675.

4. D. K. Mangwegape, N. H. Zuma, J. Aucamp and D. D. N’Da, *Archives des Pharmazie*, 2021, **354**, e2000280.

5. E. M. Czekanska, *Mol Biol*, 2011, **740**, 27-32.
